# The Role of Integrin Family in Atherosclerotic Cardiovascular Disease: A Prospective Cohort Study and Mendelian Randomization Analysis

**DOI:** 10.3390/biomedicines14081836

**Published:** 2026-08-15

**Authors:** Mengying Niu, Yuyao Feng, Keqiang Shu, Yixuan Yang, Junye Chen, Zhichao Lai, Bao Liu, Bin Peng

**Affiliations:** 1 Department of Neurology, Peking Union Medical College Hospital, Chinese Academy of Medical Sciences & Peking Union Medical College, Beijing 100730, China; 2 Department of Vascular Surgery, Peking Union Medical College Hospital, Chinese Academy of Medical Sciences & Peking Union Medical College, Beijing 100730, China

**Keywords:** atherosclerotic cardiovascular disease, integrin, UK biobank, biomarker, Mendelian randomization

## Abstract

**Background:** Atherosclerotic cardiovascular disease (ASCVD), including coronary heart disease (CHD), ischemic stroke (IS), and peripheral artery disease (PAD), represents a growing global health burden. Integrins have emerged as potential biomarkers and therapeutic targets. This study aimed to explore the role of integrins as biomarkers for ASCVD and to identify potential drug targets. **Methods:** A total of 33,210 UK Biobank participants were included. Cox proportional hazards models were used to assess associations between circulating integrin levels and ASCVD and its subtypes. Mendelian randomization and colocalization analyses were performed to investigate potential causal relationships and shared genetic variants underlying integrin levels and disease risk. **Results:** During a median follow-up of 14.04 years, 2468 participants developed ASCVD. In subtype-specific analyses, 1541 CHD events, 1050 IS events, and 590 PAD events were identified. In observational analyses, ITGA11, ITGA2, ITGAM, ITGAV, ITGB1 and ITGB2 were associated with lower ASCVD risk, whereas ITGA5 and ITGBL1 were associated with higher risk. For ASCVD mortality, ITGA11, ITGAM, ITGAV, and ITGB2 showed protective associations, while ITGAX and ITGB6 were linked to increased risk. Sex-stratified analyses revealed distinct patterns, including male-specific risk associations for ITGAX and ITGBL1 and a female-specific protective association for ITGB2. Mendelian randomization supported causal associations for five integrins, with ITGA11 showing consistency with observational findings. Colocalization analysis suggested shared causal variants between ITGAV and both CHD and IS. **Conclusions:** This study provides both observational and genetic evidence for the critical role of integrins in ASCVD, implicating their potential for assessing disease risk and serving as candidate therapeutic targets.

## 1. Introduction

Atherosclerosis, classically characterized by lipid accumulation and inflammation in large- and medium-sized arteries, is a systemic condition that can affect multiple vascular beds [1,2]. Its clinical manifestations include coronary heart disease (CHD), ischemic stroke (IS), and peripheral artery disease (PAD) [3,4,5]. Continued expansion of the lesion can cause a reduction in blood flow within the lumen, and the rupture of the unstable plaque can trigger thrombosis [6]. The main complications of atherosclerotic diseases are among the leading causes of death worldwide [7,8]. Studies have shown that the incidence of ischemic heart disease is still on the rise, and the rate of increase has been even more significant during the period from 2010 to 2019 [3]. Moreover, this trend occurs alongside a rising global burden of stroke [9].

Integrins are a widely distributed heterodimeric superfamily present on the cell membrane [10]. Each integrin consists of one α subunit and one β subunit, which are encoded by members of the ITGA and ITGB gene families, respectively. The conventional αβ designation refers to the pairing of specific α and β subunits, such as α2β1, α5β1, or αvβ3. These αβ heterodimers differ in their ligand-binding specificity. For example, α2β1 and α11β1 primarily bind collagen, α5β1 binds fibronectin, αv-containing integrins recognize RGD-containing extracellular matrix ligands, and leukocyte β2 integrins interact with ICAMs, fibrinogen, and complement-related ligands [11,12,13]. Through these ligand interactions, integrins mediate cell–cell and cell–extracellular matrix adhesion and transmit biochemical and mechanical signals between cells and their surrounding environment [14]. This function is achieved through conformational changes. Integrins play a role not only in physiological processes but also in pathological processes, such as atherosclerotic diseases [12]. In the early inflammatory phase, leukocyte integrins, including α4β1, αLβ2, αMβ2, and αXβ2, promote leukocyte adhesion, firm arrest, and transendothelial migration through interactions with endothelial adhesion molecules such as VCAM-1 and ICAM-1 [15,16]. In endothelial cells, matrix-binding integrins such as α5β1 and αvβ3 participate in disturbed flow- and oxidized LDL-induced endothelial activation, NF-κB signaling, fibronectin deposition, and pro-inflammatory gene expression, thereby contributing to endothelial dysfunction and vascular inflammation [15,17,18,19]. In addition, platelet integrin αIIbβ3 plays a central role in platelet activation, fibrinogen binding, platelet aggregation, and thrombus formation, linking integrin signaling to the thrombotic complications of advanced atherosclerotic plaques [20]. Beyond inflammatory and thrombotic responses, integrins also regulate vascular smooth muscle cell migration, phenotypic modulation, extracellular matrix remodeling, and fibrous cap formation, thereby influencing plaque progression and stability [15,21]. Therefore, integrin signaling may represent a mechanistic bridge connecting vascular inflammation, endothelial dysfunction, thrombosis, and plaque remodeling in atherosclerotic cardiovascular disease. Integrins are also established therapeutic targets and diagnostic tools for various diseases [22]. Currently, there are drugs targeting integrin sites that have been applied in clinical practice [23,24]. Nevertheless, systematic investigations into the relationship between the integrin family and atherosclerotic cardiovascular disease (ASCVD) are lacking. ASCVD presents diagnostic challenges due to its heterogeneous nature, and few current therapies can reverse the progression of atherosclerosis.

To fill this knowledge gap, we systematically evaluated the association between plasma integrins and the incidence and mortality of ASCVD in the UK Biobank. We also wanted to know if integrins could serve as potential drug targets for atherosclerotic cardiovascular disease. Our work aims to identify novel integrin-based targets with the goal of facilitating intervention in atherosclerotic diseases.

## 2. Methods

### 2.1. Study Population

**Figure 1:**
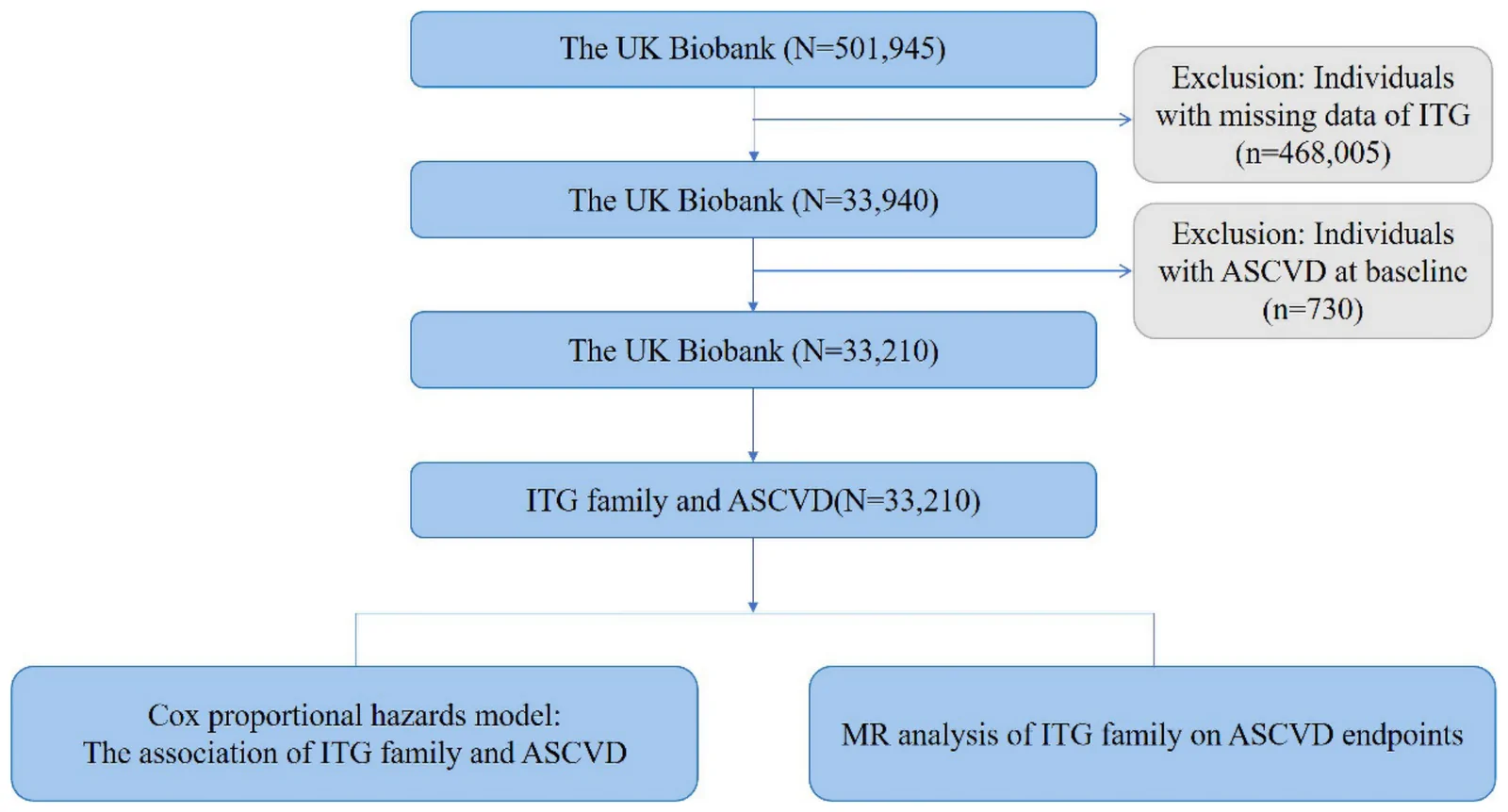
Study flowchart. ITG, integrin; ASCVD, atherosclerotic cardiovascular disease; MR, Mendelian randomization.

The UK Biobank (UKB) is a large prospective cohort study that recruited more than 500,000 participants aged 37 to 73 years from the United Kingdom. It provides extensive information from questionnaires, interviews, physical measurements, biological samples, laboratory assays, and linked health-related records [25]. The UK Biobank study was approved by the North West Multi-centre Research Ethics Committee (MREC) and was conducted in accordance with the ethical principles of the Declaration of Helsinki. All participants provided written informed consent at recruitment. This research was conducted using the UK Biobank Resource, under Application Number 197266. The present study was based on de-identified data obtained from the UK Biobank and conducted in accordance with the approved UK Biobank access procedures; therefore, no additional institutional ethical approval was required for this secondary analysis. From the initial cohort of 501,945 participants, we excluded those with prevalent ASCVD at baseline or incomplete plasma protein data [26]. Consequently, 33,210 individuals were included in the final analysis. The follow-up period ended on 31 October 2022. The study’s end date was defined as the date of death, withdrawal from the study, or the end of the follow-up period, whichever occurred first [27]. The overall research design and workflow are presented in Figure 1.

### 2.2. Blood Proteomics

The plasma protein data of the UKB database were obtained from the Olink Explore 3072 platform. Blood samples were collected from participants in EDTA tubes stored at −80 °C, and plasma proteins were quantified using Olink technology based on the Proximity Extension Assay (PEA) method [28,29]. Protein abundance was reported as Normalized Protein eXpression (NPX) values, which had already undergone Olink’s standard quality control and normalization procedures and are expressed on a log2 scale. The Olink assays quantify circulating protein abundance and do not directly determine integrin heterodimer pairing or cell-surface activation status. Ultimately, 16 integrins were available for analysis, including ITGA11, ITGA2, ITGA5, ITGA6, ITGAL, ITGAM, ITGAV, ITGAX, ITGB1, ITGB1BP1, ITGB1BP2, ITGB2, ITGB5, ITGB6, ITGB7, and ITGBL1.

### 2.3. Covariates

To minimize potential confounding, candidate covariates were initially selected based on previously published literature, clinical relevance, data availability, and statistical considerations [30]. These included baseline demographic factors such as age, race, education level, and the Townsend deprivation index (TDI) [31,32,33]. The TDI serves as a metric for socioeconomic deprivation, with higher values indicating greater deprivation. To ensure statistical robustness, racial groups with small sample sizes (including Asian, Black, and Mixed Race) were combined into an “Other” category. Anthropometric measures included waist circumference. Blood biochemical indices, including triglycerides (TGs) and high-density lipoprotein (HDL) cholesterol, were also adjusted for. The detailed detection methods have been described in previous literature [34]. To address potential bias from missing data, multiple imputation was performed for missing covariates using the Multivariate Imputation by Chained Equations (MICE) package (V 3.18.0).

### 2.4. Outcomes

The primary outcome was the incidence of ASCVD events, defined as a composite of coronary heart disease, ischemic stroke, and peripheral artery disease [35]. The secondary outcome was ASCVD mortality. Diagnostic information was obtained from hospital records, with all conditions coded according to the ICD-10 classification. For the composite ASCVD endpoint, each participant was counted only once according to the earliest occurrence of any ASCVD component event during follow-up. CHD, IS, and PAD were also analyzed separately as subtype-specific outcomes. These component outcomes were not mutually exclusive. Detailed outcome definitions and codes are provided in the Supplementary Materials (Table S1).

### 2.5. Statistical Analysis

Normally distributed baseline demographic variables are presented as mean ± standard deviation, non-normally distributed variables as median (interquartile range), and categorical variables as frequency (percentage). Group comparisons were performed using *t*-tests or Wilcoxon rank-sum tests for continuous variables, as appropriate, and chi-square tests for categorical variables. Integrin levels were reported as log2-scaled Olink NPX values and are presented as mean ± SD. Comparisons of integrin levels between participants with and without incident ASCVD were performed using Welch’s two-sample *t*-tests. Cox proportional hazards models were used to estimate hazard ratios (HRs) and their 95% confidence intervals (CIs) for the association between plasma integrin levels and ASCVD risk. Candidate covariates were considered based on prior literature, clinical relevance, data availability, and proportional hazards diagnostics. Three models were constructed: Model 1 was adjusted for age only; Model 2 additionally included race, education level, TDI, and waist circumference; and Model 3 further incorporated triglyceride and high-density lipoprotein cholesterol levels based on Model 2. A broader range of conventional ASCVD risk factors was evaluated during model construction; variables that did not consistently satisfy the proportional hazards assumption across outcome-specific models or led to model instability were not retained in the final primary models. We assessed the hazard ratios (95% CIs) for ASCVD incidence and mortality by quartiles of integrin levels [30]. Dose–response relationships were examined using restricted cubic spline (RCS) regression. As the variable “sex” violated the proportional hazards assumption, it was not included as a covariate in the primary models; instead, sex-stratified analyses were performed. For all other covariates, the proportional hazards assumption was assessed, and no violations were detected. All analyses were carried out using R software (version 4.4.3). Two-sided statistical tests were applied, and a *p*-value below 0.05 was defined as statistically significant. Cox regression analyses were implemented with the R survival package (version 3.8-3).

For the Mendelian randomization (MR) analysis, we selected cis-acting protein quantitative trait loci (cis-pQTLs) associated with integrin levels from a genome-wide association study (GWAS) in the UK Biobank [28]. Cis-pQTLs were defined as genetic variants located within 1000 kb of the transcription start site of the corresponding protein-coding gene. Genome-wide significant variants associated with integrin levels were selected as instrumental variables (*p* < 5 × 10^−8^), and linkage disequilibrium clumping was performed to retain independent variants (R^2^ < 0.01). Summary-level data for ASCVD outcomes were obtained from the FinnGen consortium (https://www.finngen.fi/en, accessed on 1 October 2025). The three core MR assumptions were addressed as follows. First, for the relevance assumption, only genome-wide significant cis-pQTLs associated with circulating integrin levels were used as instrumental variables. Second, for the independence assumption, instruments were restricted to cis-pQTLs located near the corresponding integrin-encoding genes, which may reduce the likelihood of confounding and horizontal pleiotropy compared with trans-pQTLs. Selected variants were further queried in the GWAS Catalog to identify reported associations with major cardiovascular risk factors, including lipid traits, blood pressure, diabetes-related traits, body mass index, and smoking-related traits, where available. Third, for the exclusion restriction assumption, the use of cis-pQTL-based instruments was intended to minimize potential horizontal pleiotropy, and colocalization analyses were further performed to evaluate whether circulating integrin levels and ASCVD outcomes shared the same causal variants at the corresponding genetic loci. All MR analyses were carried out using the TwoSampleMR package (version 0.6.22) in R (version 4.4.3).

To examine whether ITG levels and ASCVD share a common genetic basis, we performed colocalization analysis using the Coloc R package (version 5.2.3). This approach employs a Bayesian framework to compare five mutually exclusive hypotheses regarding the genetic association of two traits within a specified genomic region: (1) H_0_: Neither trait is genetically associated; (2) H_1_: Only ITG is associated; (3) H_2_: Only ASCVD is associated; (4) H_3_: Both traits are associated but through distinct causal variants; and (5) H_4_: Both traits share a common causal variant. The method computes posterior probabilities (PPs) for each hypothesis. Evidence for colocalization was considered strong if PP.H_4_ > 0.8, and moderate if 0.5 < PP.H_4_ ≤ 0.8 [36].

### 2.6. Use of Generative AI

During the preparation of this manuscript, ChatGPT(version 5.4) by OpenAI was used only for language polishing. The tool was not used for study design, data collection, statistical analysis, figure generation, data interpretation, or generation of scientific conclusions. All AI-assisted text was reviewed and edited by the authors, who take full responsibility for the content of this publication.

### 2.7. Sensitivity Analysis

To assess the robustness of the observed associations, we performed the following sensitivity analyses: (1) excluding participants diagnosed with ASCVD during the initial two years of follow-up to reduce potential reverse causality; (2) performing a complete-case analysis after removing individuals with any missing baseline data [30].

## 3. Results

### 3.1. Participant Characteristics

Demographic and clinical characteristics of the participants are presented in Table 1. A total of 2468 participants experienced ASCVD events, with a median follow-up period of 14.04 years. We further differentiated the ASCVD events into CHD, IS, and PAD. There were 1541 cases of CHD with a median follow-up of 14.04 years, 1050 cases of IS with a median follow-up of 14.12 years, and 590 cases of PAD with a median follow-up of 14.13 years. Compared with participants without ASCVD, participants with ASCVD were typically older and had a higher proportion of males. Additionally, they had a higher TDI, larger waist circumference, lower education levels, higher triglyceride levels, and lower HDL cholesterol levels. There was no statistical difference between the ethnic composition of the two groups, with a higher proportion of whites overall. Significant differences were observed in the circulating levels of several integrins (Table 2).

### 3.2. Association of ITG Family with the Risk of ASCVD Incidence

**Figure 2:**
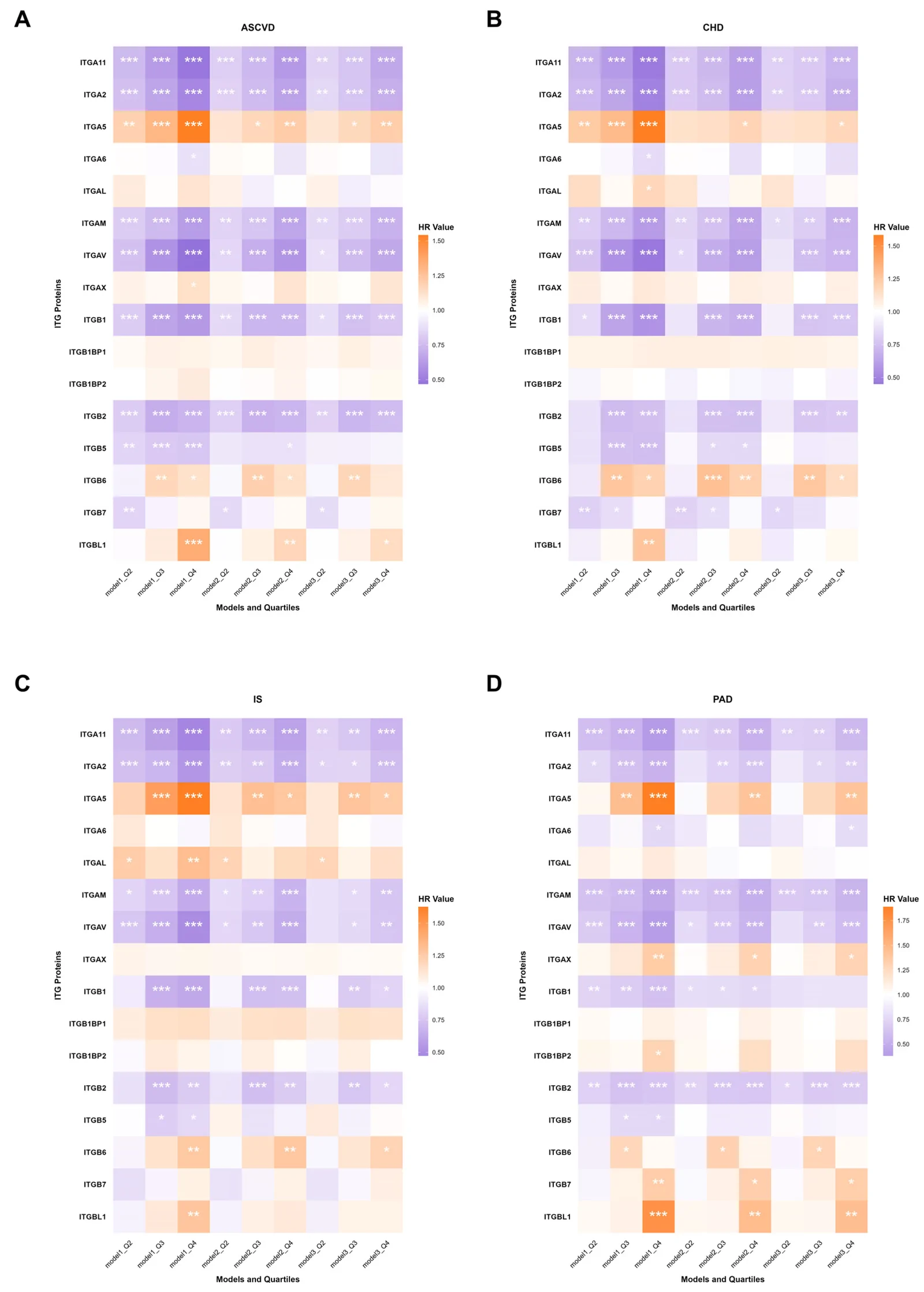
Integrins and atherosclerotic cardiovascular disease risks. (**A**) ASCVD risk associations by model and quartile; (**B**) CHD risk associations by model and quartile; (**C**) IS risk associations by model and quartile; (**D**) PAD risk associations by model and quartile. * *p* < 0.05, ** *p* < 0.01, *** *p* < 0.001. Orange: Hazard factor (HR > 1). Purple: Protective factor (HR < 1). ASCVD, atherosclerotic cardiovascular disease; CHD, coronary heart disease; IS, ischemic stroke; PAD, peripheral artery disease; ITG, Integrin.

**Figure 3:**
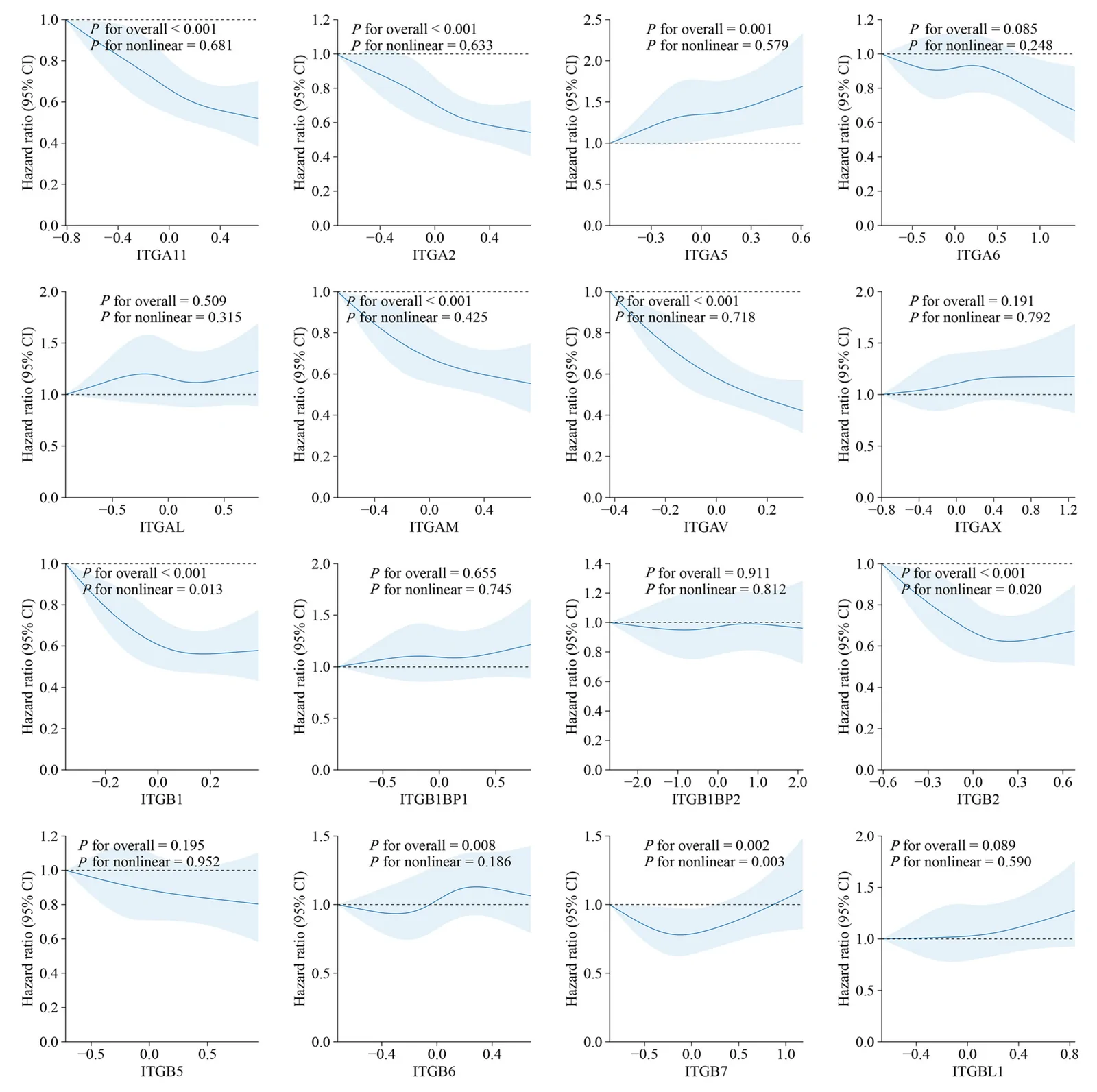
The dose–response relationship between ITG and ASCVD. The solid blue curves represent the estimated dose–response associations, and the blue shaded areas indicate the corresponding 95% confidence intervals. ITG, Integrin; ASCVD, atherosclerotic cardiovascular disease.

Figure 2 presents the relationship between the level of the integrin family and the incidence of ASCVD. In the multivariable-adjusted Cox regression models for ASCVD outcomes (Figure 2A), ITGA11, ITGA2, ITGAM, ITGAV, ITGB1, and ITGB2 were identified as protective factors, while ITGA5 and ITGBL1 were risk factors, consistent with the findings presented in Figure 3. The dose–response relationships illustrated in the figure indicated approximately linear associations for ITGA11, ITGA2, ITGAM, ITGAV and ITGA5, whereas ITGB1 and ITGB2 exhibited a non-linear pattern. Similarly, in the analysis with CHD as the outcome (Figure 2B), the protective proteins remained identical to those in ASCVD, with ITGA5 and ITGB6 emerging as risk factors. The restricted cubic spline (RCS) curves in Figure S1 revealed roughly linear trends for ITGA11, ITGA2, ITGAM, ITGAV, and ITGB1, while ITGB2 plateaued at levels above 0.3. For IS outcomes (Figure 2C), the protective and risk factors aligned with those observed in CHD. Specifically, ITGA11, ITGA2, ITGA5, ITGAV, and ITGB6 demonstrated generally linear associations (Figure S2). In contrast, the PAD analysis (Figure 2D) showed the fewest protective factors (ITGA11, ITGA2, ITGAM, ITGAV, and ITGB2) and the most risk factors (ITGA5, ITGAX, ITGB7, and ITGBL1). Among these, ITGA2 and ITGAV exhibited linear relationships, while ITGA11 displayed a broad, shallow U-shaped trend, ITGAM showed a clear L-shaped pattern, and ITGB2 presented a statistically significant U-shaped association (Figure S3). Detailed hazard ratios (HRs) and their corresponding 95% CIs are provided in the Supplementary Materials (Tables S2–S5).

### 3.3. Subgroup Analyses

**Figure 4:**
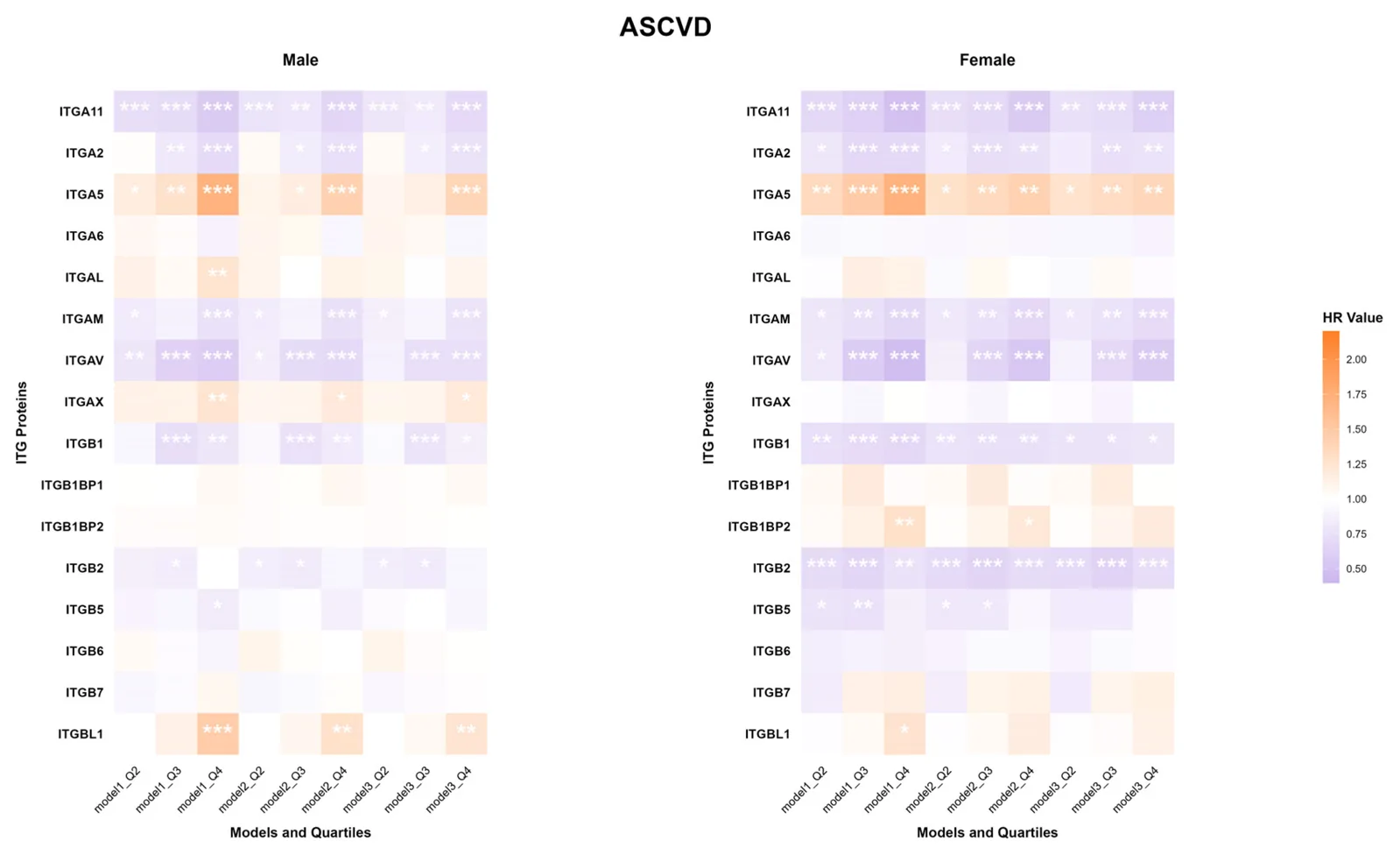
Sex-stratified ASCVD risk associations of integrins. * *p* < 0.05, ** *p* < 0.01, *** *p* < 0.001. Orange: Hazard factor (HR > 1). Purple: Protective factor (HR < 1). ASCVD, atherosclerotic cardiovascular disease.

We performed sex-stratified analyses for ASCVD and its subtype outcomes (Figure 4 and Figures S4–S6). For ASCVD, ITGA11, ITGA2, ITGAM, ITGAV, and ITGB1 were protective factors, while ITGA5 was a risk factor. ITGAX and ITGBL1 were specific risk factors in males. In CHD analysis, ITGA11, ITGA2, ITGAM, and ITGAV were common protective factors, with ITGA5 as a common risk factor. Males exhibited a unique protective factor (ITGA6) and risk factor (ITGAL); in females, ITGB2 emerged as a sex-specific protective factor in Models 2 and 3, while ITGB1 showed a more pronounced protective effect. For IS outcomes, ITGA11 and ITGA2 were shared protective factors, and ITGA5 was a shared risk factor. Regarding sex-specific factors, ITGAL was a risk factor specific to males, while ITGB2 was a protective factor specific to females. Additionally, the protective effects of ITGAM, ITGAV, and ITGB1 were more pronounced in females than in males. The PAD analysis revealed the most pronounced sex-specific patterns. Common protective factors included ITGA11, ITGAM, and ITGAV, and common risk factors included ITGB7. Male-specific factors included risk factors ITGAX, ITGB1BP2, and ITGBL1. A female-specific protective factor was ITGA6. ITGA5 acted as a more significant risk factor in males, while ITGB2 served as a more pronounced protective factor in females; furthermore, the protective effect of ITGA2 was also more significant in males. Detailed hazard ratios with 95% CIs are provided in the Supplementary Materials (Tables S6–S9).

### 3.4. Association of ITG Family with the Risk of ASCVD Mortality

**Figure 5:**
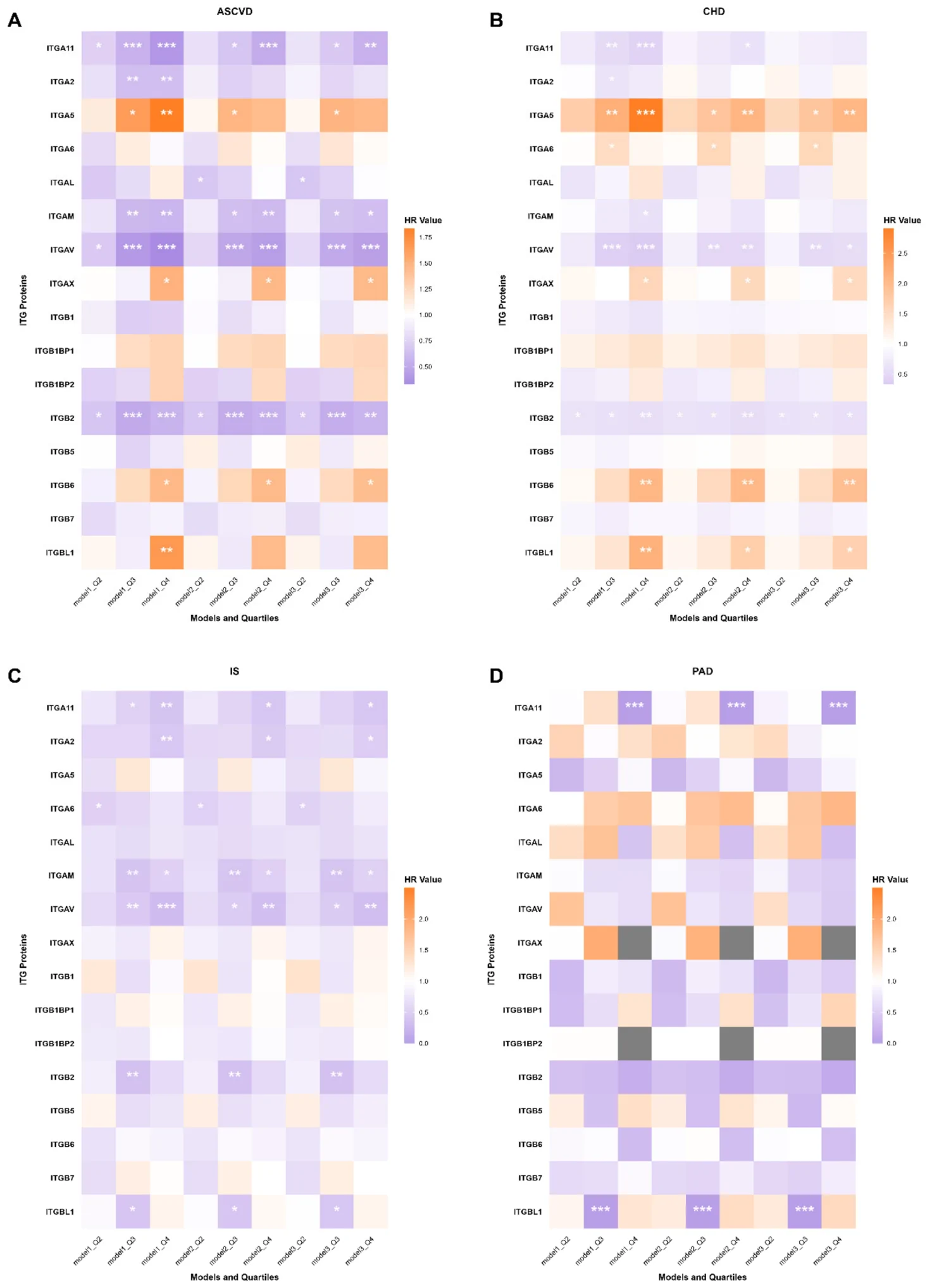
Integrins and atherosclerotic cardiovascular disease mortality risks. (**A**) ASCVD mortality risk associations by model and quartile; (**B**) CHD mortality risk associations by model and quartile; (**C**) IS mortality risk associations by model and quartile; (**D**) PAD mortality risk associations by model and quartile. * *p* < 0.05, ** *p* < 0.01, *** *p* < 0.001. Orange: Hazard factor (HR > 1). Purple: Protective factor (HR < 1). ASCVD, atherosclerotic cardiovascular disease; CHD, coronary heart disease; IS, ischemic stroke; PAD, peripheral artery disease; ITG, integrin.

The Cox regression model with multiple covariate adjustments demonstrated the relationship between ITG levels and the risk of ASCVD and its subtype mortality (Figure 5A–D). Over a median follow-up period of 14.13 years, there were 269 deaths from ASCVD, including 171 due to CHD, 88 from IS, and 10 attributed to PAD. As presented in Figure 5A–C, ITGAV was identified as a common protective factor against ASCVD mortality in analyses of its major components. Higher levels of ITGAV were associated with a lower risk of death from ASCVD, CHD and IS. In Model 3, the highest quartile (Q4) of ITGAV levels was associated with up to a 67.2% reduction in the risk of ischemic stroke (IS) mortality compared with the lowest quartile (Q1) (HR = 0.328, 95% CI: 0.165–0.652). Similar to the results of the previous disease incidence, multiple integrins can serve as risk factors or protective factors for the risk of ASCVD-related mortality. Notably, elevated ITGAX levels predict a higher risk of mortality from ASCVD and CHD, whereas in terms of disease onset, they are only associated with an increased risk of PAD. Although ITGB1 acts as a protective factor against the occurrence of ASCVD, it does not show statistical significance in relation to ASCVD mortality risk. A comprehensive set of hazard ratios with their 95% CIs is available in the Supplementary Materials (Tables S10–S13).

### 3.5. MR Analyses

In this study, we utilized genome-wide association study (GWAS) summary statistics from the FinnGen R12 database. FinnGen is a large-scale public–private partnership that has generated genomic data for over 500,000 participants from Finnish biobanks, integrating this information with longitudinal health record data to identify genotype–phenotype associations. The analysis included 56,650 CHD cases and 443,698 controls; 53,492 stroke cases and 360,342 controls; and 3153 PAD cases and 463,106 controls. We employed two-sample Mendelian randomization to explore the causal relationship between integrins and ASCVD outcomes. Genetic instruments were selected and evaluated according to the three core assumptions of Mendelian randomization. Five integrins showed potential causal relationships (Table 3): ITGB7, ITGAV, and ITGA11 were associated with CHD; ITGAV was linked to stroke; and ITGAM demonstrated a potential causal relationship with PAD. Notably, the effect of ITGA11 (OR = 0.92, 95% CI: 0.86–0.98) was consistent with findings from observational studies.

### 3.6. Colocalization Analyses

Colocalization analysis (Table S14) revealed that one out of the eleven integrins exhibited a high posterior probability (PP > 0.8) for hypothesis H_4_, providing strong genetic evidence for shared causal variants between ITGAV and IS. Furthermore, ITGAV showed moderate evidence (PP between 0.5 and 0.8) for colocalization with CHD.

### 3.7. Sensitivity Analyses

Sensitivity analyses supported the robustness of the primary findings. The associations between integrins and ASCVD incidence remained largely unchanged after excluding participants who developed ASCVD within the first two years of follow-up. Similar results were obtained in a complete case analysis that omitted imputation for missing covariates (Supplementary Tables S15–S22).

## 4. Discussion

In this study, we utilized the proteomics and genomics data from the UK Biobank to investigate the relationship between the integrin family and the atherosclerosis-related endpoint events. Our aim was to explore the potential biomarkers and therapeutic targets of ASCVD. Our results revealed distinct integrin profiles associated with different ASCVD outcomes: eight integrins were associated with ASCVD, eight with CHD, eight with IS, and nine with PAD. Furthermore, sex-stratified analyses revealed notable gender-specific differences in the relationships between integrins and ASCVD outcomes. Through Mendelian randomization and colocalization analyses, we further provided genetic evidence supporting a causal role of specific integrins in ASCVD events. Sensitivity analyses confirmed the robustness of these findings. Our research emphasizes the crucial role of integrins in ASCVD. To our knowledge, this is the first comprehensive investigation of integrins in atherosclerotic cardiovascular disease. Our findings highlight the integrin family as promising biomarkers and therapeutic targets.

Previous studies have confirmed the potential of integrins as biomarkers of diseases. For instance, ITGB5 can serve as a potential biomarker for gastric cancer [37]. And ITGB6 is associated with idiopathic pulmonary fibrosis [38]. ITGB1 can serve as a potential biomarker for the diagnosis of pancreatic cancer, with an area under the curve reaching 0.86 [39]. Integrins have also been studied in neurological diseases. In the field of neurodegenerative diseases, previous studies have shown that ITGAV is negatively correlated with Parkinson’s disease [26]. In agreement with prior studies, our multivariable-adjusted observational models confirmed several integrins as diagnostic biomarkers for ASCVD and its specific endpoints. Key integrins demonstrating consistent significance across overall and endpoint-specific analyses included: ITGA11, ITGA2, ITGA5, ITGAM, ITGAV, and ITGB2. Among these, ITGA5 is established as a risk factor, linking its higher circulating levels to an increased incidence of atherosclerosis-related events, thereby underscoring its potential pathogenic roles.

Beyond ASCVD incidence, integrins also demonstrated significant associations with ASCVD mortality risk. Consistent with our prior findings, ITGA11, ITGAM, ITGAV, and ITGB2 were identified as protective factors against fatal ASCVD events. These findings suggest that modulating circulating levels of these integrins may confer protective effects and could aid in identifying individuals at high risk for atherosclerosis-related events. Atherosclerotic cardiovascular events are heterogeneous in nature. However, the precise identification and diagnosis of distinct atherosclerotic disease types remain challenging. Our findings demonstrate that integrins offer discriminative value for different atherosclerotic outcomes. For instance, among the component outcomes of ASCVD, ITGB6 was associated with increased risks of CHD and IS, while ITGAX, ITGB7, and ITGBL1 were specifically linked to PAD risk. Disease-specific integrins may contribute to the risk stratification of different atherosclerotic diseases. Collectively, these findings suggest that circulating integrin-related proteins may serve as blood-based cardiovascular risk-associated biomarkers for ASCVD risk assessment. In addition, the genetic evidence from colocalization analysis supports ITGAV as a candidate molecular target for further mechanistic investigation and future therapeutic exploration in atherosclerosis.

Sex-stratified Cox regression analyses identified distinct associations between integrins and ASCVD risks. In our results, ITGB2 had a more pronounced protective effect in females, while ITGAX and ITGBL1 indicated higher ASCVD risk in males. The differential susceptibility to atherosclerotic events between sexes has been previously reported [40]. Existing literature indicates that ITGB1 functions as a sex-specific molecule on T cells, implying a potential basis for sex-differentiated integrin expression [41]. Our study now extends this concept by demonstrating for the first time that there is a gender difference in the expression of various integrins in ASCVD events, thereby providing initial evidence for their contribution to sex-specific disease risk. These findings pave the way for the future use of integrins as diagnostic biomarkers for atherosclerotic related events. Sex-specific explanations are necessary when evaluating integrins as diagnostic biomarkers for atherosclerotic disease, as their significance may profoundly vary by sex.

Integrins are involved in mediating signaling pathways for normal cellular physiological processes. In pathological mechanisms such as tumors, they are involved in processes such as invasion and metastasis [42]. At present, CAR-T cell therapy targeting integrins is already undergoing clinical trials. The αvβ6-CAR-T cell therapy has demonstrated therapeutic potential in solid tumors such as cholangiocarcinoma [43]. Previous studies, particularly the review by Finney et al., have highlighted the involvement of integrin signaling in multiple stages of atherosclerosis [15]. These established mechanistic roles provide a biological framework for interpreting our findings. The associations observed in the present study are unlikely to reflect a single pathway, but may capture several integrin-related processes involved in ASCVD development and progression. For example, leukocyte- and endothelial-associated integrin signaling is closely linked to vascular inflammation and endothelial activation, whereas platelet and matrix-binding integrins may contribute to thrombotic events, extracellular matrix turnover, and plaque remodeling [15,16,17,18,19,20,21]. Furthermore, oxidized low-density lipoprotein (Ox-LDL) acts as a key agonist by activating ITGA5 and inducing the ITGA5-dependent signaling pathway, thereby contributing to disease progression [44]. This study is consistent with the role of ITGA5 as a risk factor for ASCVD in our study. Therefore, altered circulating levels of integrin-related proteins may reflect systemic immune activation, endothelial dysfunction, platelet activation, or vascular wall remodeling, which could partly explain their associations with incident ASCVD and ASCVD-related mortality. However, because circulating Olink measurements do not directly determine the cellular source, heterodimer formation, or activation state of cell-surface integrins, these mechanistic interpretations remain inferential and require further experimental validation.

Our MR analysis utilizing GWAS data identified five proteins with putative causal effects on ASCVD. An interesting divergence was observed: while most integrins appeared to be protective factors in the observational analysis, all except ITGA11 were indicated as risk factors in the MR analysis. Since the MR method effectively minimizes confounding and reverse causation issues inherent in observational studies, its results provide more reliable evidence for causal inference. The observed discrepancies are likely attributable to the limited panel of integrins (only eleven) available in the UK Biobank GWAS dataset, alongside potential unmeasured confounding factors. Notably, ITGA11 was consistently identified as a protective factor in both analytical approaches, which significantly strengthens its credibility as a potential protective biomarker and therapeutic target for ASCVD. Currently, research on ITGA11 is focused on fibrosis [45]. Our study suggests that ITGA11 could be a potential research direction for atherosclerosis. In this study, the colocalization analysis provided strong genetic evidence for the common pathogenic variations between ITGAV and CHD, as well as IS, and this was supported by observational analysis. Analysis of the GEO database further revealed that reduced ITGAV gene levels promote atherosclerotic plaque formation in patients with familial hypercholesterolemia [46]. Previous studies have shown that specific deletion of integrin α5 or αv in endothelial cells in vivo can reduce endoplasmic reticulum stress in vulnerable atherosclerotic regions under OxLDL stimulation. Mechanistically, endoplasmic reticulum stress promotes endothelial cell activation via the JNK/c-Jun signaling pathway, thereby amplifying endothelial inflammation [47]. In addition, convallatoxin has been reported to exert an anti-atherosclerotic effect partly by activating the PPARγ-integrin αvβ5 signaling pathway, which promotes the polarization of M2 macrophages [48]. Furthermore, experimental evidence from mouse models identified αvβ3 as the major integrin heterodimer mediating shear stress-induced pro-inflammatory responses, highlighting its role as a key factor in the early inflammatory process of atherosclerosis [18]. Supporting this, a clinical study using 18F-fluciclatide PET to analyze human aortic atherosclerotic plaques suggested that the in vivo αvβ3 integrin expression correlates with plaque burden and is elevated in patients with recent myocardial infarction [49]. Collectively, our findings are consistent with these earlier observations and suggest that ITGAV contributes to atherosclerosis through multiple distinct mechanisms. This study thus provides a rationale for exploring ITGAV as a potential diagnostic and therapeutic target in atherosclerosis onset and progression, offering a theoretical foundation for designing novel drugs and interventions aimed at this molecule. Nevertheless, although cis-pQTL instruments and colocalization analyses were used to reduce potential horizontal pleiotropy, the independence and exclusion restriction assumptions cannot be fully proven. Moreover, the limited number of available instruments for some integrins restricted the use of conventional pleiotropy tests, such as MR-Egger regression. Therefore, the MR findings should be interpreted cautiously as supportive genetic evidence.

Due to the heavy disease burden caused by atherosclerotic diseases, the development of new biomarkers and the search for new effective drug targets have become unmet needs [1]. Blood biomarkers have received considerable attention in clinical practice due to their simplicity and relatively low cost [50]. Novel non-invasive diagnostic biomarkers facilitate the assessment of risk associated with atherosclerotic diseases. Current therapeutic strategies for atherosclerosis primarily focus on targeting systemic risk factors, such as the use of lipid-lowering medications [51]. However, a notable gap remains in the availability of therapeutics that directly address specific molecular targets within the disease pathway. Vedolizumab and natalizumab are antibodies against leukocyte integrins α4β7 and α4β1, and have been used to treat Crohn’s disease and multiple sclerosis [22,23]. It is expected that drugs targeting integrin receptors will be developed in the future for the treatment of atherosclerosis. This article demonstrates the potential of the integrin family as novel blood biomarkers for identifying atherosclerotic cardiovascular events. Identifying the risk of ASCVD and initiating interventions as early as possible can help reduce the burden of atherosclerotic related disease. Our research has provided new insights into the relationship between the integrin family and atherosclerotic cardiovascular disease, and is expected to offer new diagnostic ideas for disease and potential drug targets in the future. Despite this potential clinical relevance, the biological specificity of circulating integrins requires further clarification. Integrins participate in diverse biological processes, including immune regulation, chronic inflammation, fibrosis, cancer progression, thrombosis, and extracellular matrix remodeling. Thus, the observed associations with ASCVD outcomes may capture integrin-related vascular pathology as well as broader systemic inflammatory, immune, or remodeling activity. These findings support the potential value of circulating integrin-related proteins as cardiovascular risk-associated biomarkers, while further validation is needed to determine their disease specificity and incremental predictive value beyond established cardiovascular risk factors and general inflammatory markers. However, the Olink Explore 3072 platform used in this study is a high-throughput proteomic research platform and is not currently a routine clinical test. Before clinical application, candidate integrin-related biomarkers require validation using more accessible targeted assays, such as ELISA-based or targeted multiplex platforms, with further evaluation of their reproducibility and incremental predictive value.

The major strengths of this work include the leveraged UK Biobank resource, which provides a large sample size with extensive phenotypic data. And we use Mendelian randomization to mitigate confounding and reverse causality. The robustness of the results is further bolstered by comprehensive sensitivity analyses. Our study has several limitations. First, both the proteomic and genomic data were derived mainly from European populations, which may limit the generalizability of our findings to other ethnic groups. In the future, a larger and more diverse group is needed to further confirm our findings. Second, the Olink platform measures circulating integrin subunits or integrin-related proteins rather than intact cell-surface αβ heterodimers. Because integrin-mediated mechanisms in atherosclerosis are largely driven by cell-surface integrins on leukocytes, endothelial cells, platelets, and vascular smooth muscle cells, these circulating plasma proteins should be interpreted cautiously as indirect biomarkers. Their plasma abundance may reflect processes such as extracellular domain shedding, extracellular vesicle-associated proteins, inflammatory cell activation, endothelial or platelet activation, and extracellular matrix remodeling; however, they should not be considered direct measurements of cell-surface integrin activity, ligand-binding function, or downstream signaling. Further mechanistic studies are needed to clarify how circulating integrin-related proteins reflect cell-surface integrin biology in ASCVD. Third, statin use and other lipid-lowering therapies were not included in the final primary Cox models. Therefore, residual confounding by unmeasured or incompletely adjusted factors, including the potential lipid-lowering, anti-inflammatory, and immunomodulatory effects of these therapies, cannot be fully excluded.

## 5. Conclusions

In aggregate, our study identified multiple integrins as potential biomarkers for predicting the risk of both incidence and mortality associated with ASCVD. Several of these integrins demonstrated sex-specific associations. Furthermore, colocalization analyses provided strong support for shared genetic variants between ITGAV and both coronary heart disease and stroke. This study underscores the translational potential of integrins as both biomarkers and therapeutic targets in ASCVD. Translating this potential into clinical application calls for dedicated mechanistic studies and subsequent clinical validation.

## Figures and Tables

**Table 1:** Baseline characteristics of participants in the study (N = 33,210).

	Overall	Without Incident ASCVD	Incident ASCVD	
	N = 33,210	n = 30,742	n = 2468	*p*
Age, years	56.7 (8.21)	56.3 (8.20)	61.2 (6.82)	<0.001
Sex				<0.001
Female	18,125 (54.58%)	17,177 (55.87%)	948 (38.41%)	
Male	15,085 (45.42%)	13,565 (44.13%)	1520 (61.59%)	
TDI	−1.18 (3.19)	−1.22 (3.17)	−0.67 (3.37)	<0.001
Waist	90.2 (13.4)	89.8 (13.3)	95.4 (13.9)	<0.001
Ethnicity				0.288
White	31,043 (93.47%)	28,723 (93.43%)	2320 (94.00%)	
Other	2167 (6.53%)	2019 (6.57%)	148 (6.00%)	
Qualification				<0.001
Low	7139 (21.50%)	6471 (21.05%)	668 (27.07%)	
Intermediate	13,158 (39.62%)	12,142 (39.50%)	1016 (41.17%)	
Higher	12,913 (38.88%)	12,129 (39.45%)	784 (31.77%)	
Triglycerides, mmol/L	1.74 (1.03)	1.72 (1.02)	1.96 (1.07)	<0.001
HDL cholesterol, mmol/L	1.45 (0.38)	1.46 (0.38)	1.34 (0.38)	<0.001

Normally distributed variables are presented as mean ± standard deviation, non-normally distributed variables as median (interquartile range), and categorical variables as frequency (percentage). ASCVD, atherosclerotic cardiovascular disease; TDI, Townsend deprivation index; HDL, high-density lipoprotein.

**Table 2:** Integrin levels (N = 33,210).

	Overall	Without Incident ASCVD	Incident ASCVD	
	N = 33,210	n = 30,742	n = 2468	*p*
ITGA11	−0.01 (0.39)	0.00 (0.39)	−0.10 (0.42)	<0.001
ITGA2	0.01 (0.36)	0.01 (0.36)	−0.05 (0.38)	<0.001
ITGA5	0.00 (0.29)	−0.01 (0.29)	0.07 (0.29)	<0.001
ITGA6	0.06 (0.59)	0.07 (0.59)	0.01 (0.55)	<0.001
ITGAL	−0.01 (0.45)	−0.02 (0.45)	0.02 (0.47)	<0.001
ITGAM	0.01 (0.36)	0.01 (0.36)	−0.04 (0.37)	<0.001
ITGAV	−0.01 (0.19)	−0.01 (0.19)	−0.04 (0.20)	<0.001
ITGAX	0.05 (0.54)	0.05 (0.55)	0.05 (0.51)	0.584
ITGB1	0.01 (0.24)	0.01 (0.24)	−0.01 (0.25)	0.002
ITGB1BP1	−0.01 (0.44)	−0.01 (0.44)	0.00 (0.47)	0.076
ITGB1BP2	−0.08 (1.22)	−0.08 (1.22)	−0.07 (1.21)	0.500
ITGB2	0.01 (0.33)	0.01 (0.33)	−0.01 (0.35)	0.016
ITGB5	0.02 (0.42)	0.02 (0.42)	0.03 (0.43)	0.113
ITGB6	−0.01 (0.36)	−0.01 (0.35)	0.02 (0.36)	0.001
ITGB7	0.03 (0.51)	0.03 (0.51)	0.05 (0.53)	0.023
ITGBL1	0.03 (0.40)	0.02 (0.39)	0.15 (0.43)	<0.001

ASCVD, atherosclerotic cardiovascular disease; ITG, integrin. Values are mean ± SD. Integrin levels are reported as Normalized Protein eXpression (NPX) values on a log2 scale. *p*-values were calculated using Welch’s two-sample *t*-tests.

**Table 3:** MR analysis of integrins and ASCVD.

Outcomes	Integrin	Method	*p*	OR (95% CI)
CHD	ITGB7	Wald ratio	<0.01	1.20 (1.08, 1.35)
CHD	ITGAV	Wald ratio	<0.001	1.22 (1.08, 1.37)
CHD	ITGA11	Wald ratio	0.02	0.92 (0.86, 0.98)
Stroke	ITGAV	Wald ratio	<0.001	1.26 (1.11, 1.42)
PAD	ITGAM	Wald ratio	0.04	1.38 (1.01, 1.88)

OR, Odds ratio; CHD, coronary heart disease; PAD, peripheral artery disease; ITG, integrin.

## Data Availability

This research was conducted using the UK Biobank Resource, under Application Number 197266. UK Biobank data are available to approved researchers through the UK Biobank application system. Researchers can apply for access through the UK Biobank website. Summary statistics for ASCVD outcomes were obtained from the FinnGen study.

## Supplementary Materials

The following supporting information can be downloaded at: https://www.mdpi.com/article/10.3390/biomedicines14081836/s1, Figures S1–S3: Dose–response relationships between integrins and incident CHD, IS, and PAD; Figures S4–S6: Sex-stratified risk associations of integrins with incident CHD, IS, and PAD; Table S1: Definition of atherosclerotic cardiovascular disease in this study; Table S2: HR (95% CI) of Incident ASCVD by Integrin Levels; Table S3: HR (95% CI) of Incident CHD by Integrin Levels; Table S4: HR (95% CI) of Incident IS by Integrin Levels; Table S5: HR (95% CI) of Incident PAD by Integrin Levels; Table S6: Sex-Stratified HR (95% CI) of Incident ASCVD by Integrin Levels; Table S7: Sex-Stratified HR (95% CI) of Incident CHD by Integrin Levels; Table S8: Sex-Stratified HR (95% CI) of Incident IS by Integrin Levels; Table S9: Sex-Stratified HR (95% CI) of Incident PAD by Integrin Levels; Table S10: HR (95% CI) of ASCVD Mortality by Integrin Levels; Table S11: HR (95% CI) of CHD Mortality by Integrin Levels; Table S12: HR (95% CI) of IS Mortality by Integrin Levels; Table S13: HR (95% CI) of PAD Mortality by Integrin Levels; Table S14: Colocalization analysis of the association of integrin with ASCVD; Tables S15–S18: Sensitivity analyses excluding participants who developed the corresponding outcome within the first 2 years of follow-up: Table S15: HR (95% CI) of Incident ASCVD by Integrin Levels; Table S16: HR (95% CI) of Incident CHD by Integrin Levels; Table S17: HR (95% CI) of Incident IS by Integrin Levels; Table S18: HR (95% CI) of Incident PAD by Integrin Levels; Tables S19–S22: Complete-case sensitivity analyses without imputation for missing covariates: Table S19: HR (95% CI) of Incident ASCVD by Integrin Levels; Table S20: HR (95% CI) of Incident CHD by Integrin Levels; Table S21: HR (95% CI) of Incident IS by Integrin Levels; Table S22: HR (95% CI) of Incident PAD by Integrin Levels.

## Abbreviations

ASCVD, atherosclerotic cardiovascular disease; CHD, coronary heart disease; IS, ischemic stroke; PAD, peripheral artery disease; UKB, UK Biobank; MREC: North West Multi-centre Research Ethics Committee; MR, Mendelian randomization; ITG, integrin; PEA, Proximity Extension Assay; NPX, Normalized Protein eXpression; TDI, Townsend deprivation index; TG, triglycerides; HDL, high-density lipoprotein; MICE, Multivariate Imputation by Chained Equations; HRs, hazard ratios; CIs, confidence intervals; RCS, restricted cubic spline; cis-pQTLs, cis-acting protein quantitative trait loci; GWAS, genome-wide association study; PP, posterior probabilities; OR, Odds Ratio; Ox-LDL, oxidized low-density lipoprotein.
