# Supplementary material for: The Role of Integrin Family in Atherosclerotic Cardiovascular Disease: A Prospective Cohort Study and Mendelian Randomization Analysis
Source: Biomedicines. 2026 Aug 15;14(8):1836. doi: 10.3390/biomedicines14081836 (PMC13509564; doi:10.3390/biomedicines14081836)
Supplement: Supplementary file 1 [file biomedicines-14-01836-s001.zip › TableS1.pdf]

Table S1. Definition of atherosclerotic cardiovascular disease in this study [35].

**Disease**	**Field Name**	**Code**
Coronary heart disease (CHD)	ICD-10	I21-I24, I25.2
Ischemic stroke (IS)	ICD-10	I63, I64.X
Peripheral arterial disease (PAD)	ICD-10	I70.0, I70.2, I70.8, I70.9, I73.8, I73.9

ICD, The International Classification of disease.
