# Supplementary material for: The Role of Integrin Family in Atherosclerotic Cardiovascular Disease: A Prospective Cohort Study and Mendelian Randomization Analysis
Source: Biomedicines. 2026 Aug 15;14(8):1836. doi: 10.3390/biomedicines14081836 (PMC13509564; doi:10.3390/biomedicines14081836)
Supplement: Supplementary file 1 [file biomedicines-14-01836-s001.zip › TableS10.pdf]

Table S10. HR (95% CI) of ASCVD mortality by integrin levels.

Integrin	Hazard Ratio (95% CI)		
	Model 1	Model 2	Model 3
ITGA11	1 (Reference)	1 (Reference)	1 (Reference)
Q2	**0.737 (0.546, 0.995)**	0.820 (0.608, 1.106)	0.836 (0.620, 1.126)
Q3	**0.555 (0.399, 0.773)**	**0.669 (0.478, 0.935)**	**0.691 (0.495, 0.966)**
Q4	**0.401 (0.279, 0.577)**	**0.526 (0.360, 0.767)**	**0.553 (0.376, 0.814)**
ITGA2	1 (Reference)	1 (Reference)	1 (Reference)
Q2	0.827 (0.603, 1.135)	0.907 (0.658, 1.250)	0.928 (0.673, 1.280)
Q3	**0.624 (0.445, 0.877)**	0.730 (0.519, 1.027)	0.761 (0.538, 1.075)
Q4	**0.627 (0.450, 0.875)**	0.784 (0.556, 1.105)	0.836 (0.588, 1.189)
ITGA5	1 (Reference)	1 (Reference)	1 (Reference)
Q2	1.131 (0.748, 1.711)	1.051 (0.694, 1.591)	1.053 (0.695, 1.596)
Q3	**1.646 (1.119, 2.419)**	**1.479 (1.002, 2.181)**	**1.485 (1.006, 2.193)**
Q4	**1.833 (1.260, 2.666)**	1.435 (0.973, 2.118)	1.457 (0.984, 2.159)
ITGA6	1 (Reference)	1 (Reference)	1 (Reference)
Q2	0.794 (0.562, 1.122)	0.814 (0.576, 1.151)	0.813 (0.576, 1.148)
Q3	1.118 (0.810, 1.543)	1.164 (0.843, 1.608)	1.163 (0.842, 1.606)
Q4	0.966 (0.689, 1.355)	1.021 (0.727, 1.435)	1.026 (0.730, 1.444)
ITGAL	1 (Reference)	1 (Reference)	1 (Reference)
Q2	0.698 (0.482, 1.011)	**0.679 (0.469, 0.983)**	**0.679 (0.469, 0.982)**
Q3	0.805 (0.568, 1.140)	0.752 (0.531, 1.064)	0.752 (0.531, 1.065)
Q4	1.107 (0.807, 1.519)	0.984 (0.719, 1.349)	0.986 (0.718, 1.352)
ITGAM	1 (Reference)	1 (Reference)	1 (Reference)
Q2	0.851 (0.626, 1.156)	0.884 (0.650, 1.203)	0.896 (0.658, 1.220)
Q3	**0.597 (0.424, 0.840)**	**0.638 (0.453, 0.899)**	**0.651 (0.461, 0.917)**
Q4	**0.577 (0.409, 0.813)**	**0.608 (0.428, 0.864)**	**0.633 (0.447, 0.897)**
ITGAV	1 (Reference)	1 (Reference)	1 (Reference)
Q2	**0.701 (0.518, 0.948)**	0.775 (0.571, 1.052)	0.790 (0.581, 1.075)
Q3	**0.413 (0.293, 0.581)**	**0.494 (0.348, 0.702)**	**0.509 (0.358, 0.723)**
Q4	**0.330 (0.233, 0.469)**	**0.435 (0.300, 0.631)**	**0.456 (0.312, 0.667)**
ITGAX	1 (Reference)	1 (Reference)	1 (Reference)
Q2	1.008 (0.706, 1.439)	0.981 (0.687, 1.400)	0.977 (0.684, 1.394)
Q3	0.925 (0.642, 1.331)	0.908 (0.630, 1.307)	0.903 (0.627, 1.301)
Q4	**1.502 (1.085, 2.078)**	**1.462 (1.055, 2.026)**	**1.455 (1.049, 2.017)**
ITGB1	1 (Reference)	1 (Reference)	1 (Reference)
Q2	0.906 (0.653, 1.258)	0.973 (0.700, 1.353)	0.999 (0.718, 1.390)
Q3	0.717 (0.507, 1.014)	0.809 (0.571, 1.146)	0.846 (0.596, 1.202)
Q4	0.736 (0.525, 1.031)	0.893 (0.632, 1.260)	0.961 (0.671, 1.375)
ITGB1BP1	1 (Reference)	1 (Reference)	1 (Reference)
Q2	0.984 (0.687, 1.410)	0.994 (0.693, 1.424)	0.991 (0.691, 1.420)
Q3	1.228 (0.872, 1.728)	1.236 (0.878, 1.740)	1.236 (0.878, 1.741)
Q4	1.283 (0.913, 1.802)	1.271 (0.904, 1.785)	1.268 (0.902, 1.782)

ITGB1BP2	1 (Reference)	1 (Reference)	1 (Reference)
Q2	0.741 (0.522, 1.053)	0.735 (0.517, 1.044)	0.737 (0.519, 1.045)
Q3	0.786 (0.555, 1.112)	0.772 (0.545, 1.092)	0.776 (0.548, 1.098)
Q4	1.289 (0.944, 1.761)	1.235 (0.903, 1.688)	1.244 (0.909, 1.704)
ITGB2	1 (Reference)	1 (Reference)	1 (Reference)
Q2	**0.670 (0.488, 0.921)**	**0.675 (0.491, 0.928)**	**0.681 (0.495, 0.937)**
Q3	**0.510 (0.364, 0.715)**	**0.526 (0.375, 0.738)**	**0.534 (0.380, 0.751)**
Q4	**0.567 (0.409, 0.787)**	**0.560 (0.403, 0.779)**	**0.575 (0.413, 0.800)**
ITGB5	1 (Reference)	1 (Reference)	1 (Reference)
Q2	0.993 (0.707, 1.394)	1.095 (0.778, 1.541)	1.116 (0.792, 1.571)
Q3	0.750 (0.525, 1.071)	0.851 (0.595, 1.218)	0.884 (0.617, 1.267)
Q4	0.871 (0.619, 1.225)	1.009 (0.715, 1.423)	1.065 (0.750, 1.512)
ITGB6	1 (Reference)	1 (Reference)	1 (Reference)
Q2	0.911 (0.627, 1.323)	0.934 (0.643, 1.355)	0.930 (0.641, 1.349)
Q3	1.238 (0.872, 1.757)	1.256 (0.886, 1.782)	1.244 (0.878, 1.763)
Q4	**1.464 (1.047, 2.048)**	**1.454 (1.039, 2.035)**	**1.428 (1.021, 1.998)**
ITGB7	1 (Reference)	1 (Reference)	1 (Reference)
Q2	0.790 (0.561, 1.112)	0.804 (0.570, 1.133)	0.810 (0.575, 1.141)
Q3	0.889 (0.637, 1.242)	0.887 (0.635, 1.238)	0.896 (0.641, 1.252)
Q4	0.929 (0.669, 1.289)	0.907 (0.654, 1.259)	0.912 (0.657, 1.266)
ITGBL1	1 (Reference)	1 (Reference)	1 (Reference)
Q2	1.052 (0.703, 1.575)	1.068 (0.713, 1.600)	1.071 (0.715, 1.603)
Q3	0.893 (0.591, 1.351)	0.869 (0.573, 1.319)	0.870 (0.574, 1.319)
Q4	**1.676 (1.157, 2.427)**	1.446 (0.988, 2.117)	1.442 (0.986, 2.109)

Q1 (lowest, < 25th percentile), Q2 (25th to < 50th percentile), Q3 (50th to < 75th percentile), and Q4 (highest, ⩾ 75th percentile); CI: confidence interval; ITG, Integrin.
