# Supplementary material for: The Role of Integrin Family in Atherosclerotic Cardiovascular Disease: A Prospective Cohort Study and Mendelian Randomization Analysis
Source: Biomedicines. 2026 Aug 15;14(8):1836. doi: 10.3390/biomedicines14081836 (PMC13509564; doi:10.3390/biomedicines14081836)
Supplement: Supplementary file 1 [file biomedicines-14-01836-s001.zip › TableS11.pdf]

Table S11. HR (95% CI) of CHD mortality by integrin levels.

Integrin	Hazard Ratio (95% CI)		
	Model 1	Model 2	Model 3
ITGA11	1 (Reference)	1 (Reference)	1 (Reference)
Q2	0.728 (0.499, 1.062)	0.839 (0.577, 1.219)	0.876 (0.603, 1.272)
Q3	**0.534 (0.351, 0.813)**	0.690 (0.452, 1.055)	0.750 (0.488, 1.151)
Q4	**0.433 (0.278, 0.677)**	**0.630 (0.398, 0.999)**	0.715 (0.447, 1.145)
ITGA2	1 (Reference)	1 (Reference)	1 (Reference)
Q2	0.964 (0.648, 1.432)	1.084 (0.726, 1.620)	1.128 (0.754, 1.687)
Q3	**0.640 (0.412, 0.994)**	0.784 (0.503, 1.223)	0.845 (0.537, 1.328)
Q4	0.732 (0.483, 1.110)	0.991 (0.643, 1.527)	1.108 (0.708, 1.732)
ITGA5	1 (Reference)	1 (Reference)	1 (Reference)
Q2	1.749 (0.995, 3.075)	1.584 (0.899, 2.791)	1.571 (0.891, 2.769)
Q3	**2.184 (1.264, 3.772)**	**1.878 (1.087, 3.246)**	**1.856 (1.072, 3.213)**
Q4	**2.908 (1.725, 4.901)**	**2.094 (1.226, 3.575)**	**2.078 (1.211, 3.564)**
ITGA6	1 (Reference)	1 (Reference)	1 (Reference)
Q2	1.013 (0.648, 1.583)	1.045 (0.668, 1.633)	1.036 (0.664, 1.618)
Q3	**1.527 (1.010, 2.311)**	**1.605 (1.062, 2.427)**	**1.590 (1.052, 2.403)**
Q4	1.122 (0.718, 1.753)	1.201 (0.767, 1.879)	1.191 (0.759, 1.869)
ITGAL	1 (Reference)	1 (Reference)	1 (Reference)
Q2	0.657 (0.402, 1.073)	0.635 (0.389, 1.037)	0.631 (0.387, 1.030)
Q3	0.865 (0.553, 1.353)	0.795 (0.510, 1.239)	0.787 (0.505, 1.227)
Q4	1.392 (0.936, 2.069)	1.203 (0.810, 1.785)	1.186 (0.798, 1.763)
ITGAM	1 (Reference)	1 (Reference)	1 (Reference)
Q2	0.945 (0.640, 1.396)	0.992 (0.671, 1.467)	1.019 (0.689, 1.507)
Q3	0.735 (0.483, 1.120)	0.803 (0.527, 1.222)	0.838 (0.549, 1.278)
Q4	**0.609 (0.392, 0.947)**	0.660 (0.420, 1.039)	0.717 (0.457, 1.125)
ITGAV	1 (Reference)	1 (Reference)	1 (Reference)
Q2	0.711 (0.488, 1.036)	0.816 (0.559, 1.192)	0.857 (0.583, 1.259)
Q3	**0.384 (0.248, 0.595)**	**0.491 (0.315, 0.767)**	**0.532 (0.339, 0.836)**
Q4	**0.336 (0.217, 0.521)**	**0.494 (0.313, 0.779)**	**0.562 (0.351, 0.900)**
ITGAX	1 (Reference)	1 (Reference)	1 (Reference)
Q2	1.104 (0.704, 1.731)	1.071 (0.684, 1.677)	1.061 (0.678, 1.663)
Q3	0.994 (0.626, 1.580)	0.973 (0.613, 1.546)	0.964 (0.607, 1.532)
Q4	**1.612 (1.065, 2.438)**	**1.567 (1.035, 2.374)**	**1.553 (1.025, 2.353)**
ITGB1	1 (Reference)	1 (Reference)	1 (Reference)
Q2	0.804 (0.534, 1.212)	0.878 (0.581, 1.328)	0.912 (0.602, 1.381)
Q3	0.736 (0.484, 1.118)	0.854 (0.560, 1.301)	0.917 (0.601, 1.400)
Q4	0.654 (0.429, 0.999)	0.833 (0.541, 1.282)	0.937 (0.600, 1.463)
ITGB1BP1	1 (Reference)	1 (Reference)	1 (Reference)
Q2	1.199 (0.763, 1.886)	1.213 (0.771, 1.909)	1.204 (0.765, 1.895)
Q3	1.326 (0.852, 2.065)	1.335 (0.856, 2.081)	1.327 (0.851, 2.068)
Q4	1.437 (0.928, 2.226)	1.418 (0.915, 2.198)	1.398 (0.902, 2.167)

ITGB1BP2	1 (Reference)	1 (Reference)	1 (Reference)
Q2	0.713 (0.457, 1.112)	0.706 (0.453, 1.103)	0.703 (0.451, 1.095)
Q3	0.794 (0.515, 1.225)	0.780 (0.506, 1.203)	0.777 (0.504, 1.198)
Q4	1.307 (0.887, 1.927)	1.251 (0.848, 1.845)	1.239 (0.837, 1.833)
ITGB2	1 (Reference)	1 (Reference)	1 (Reference)
Q2	**0.621 (0.411, 0.938)**	**0.623 (0.412, 0.941)**	**0.631 (0.418, 0.954)**
Q3	**0.608 (0.405, 0.913)**	**0.630 (0.419, 0.946)**	**0.645 (0.429, 0.972)**
Q4	**0.574 (0.380, 0.869)**	**0.566 (0.373, 0.858)**	**0.591 (0.390, 0.896)**
ITGB5	1 (Reference)	1 (Reference)	1 (Reference)
Q2	0.910 (0.587, 1.410)	1.029 (0.662, 1.598)	1.066 (0.685, 1.657)
Q3	0.832 (0.536, 1.293)	0.985 (0.632, 1.534)	1.056 (0.677, 1.648)
Q4	0.916 (0.597, 1.404)	1.111 (0.721, 1.710)	1.211 (0.779, 1.881)
ITGB6	1 (Reference)	1 (Reference)	1 (Reference)
Q2	1.082 (0.657, 1.782)	1.115 (0.678, 1.834)	1.101 (0.670, 1.808)
Q3	1.524 (0.956, 2.429)	1.550 (0.973, 2.469)	1.511 (0.950, 2.404)
Q4	**2.077 (1.339, 3.221)**	**2.060 (1.327, 3.197)**	**1.963 (1.268, 3.039)**
ITGB7	1 (Reference)	1 (Reference)	1 (Reference)
Q2	0.870 (0.576, 1.315)	0.890 (0.588, 1.346)	0.898 (0.594, 1.357)
Q3	0.766 (0.498, 1.177)	0.765 (0.498, 1.177)	0.774 (0.503, 1.190)
Q4	0.886 (0.587, 1.337)	0.865 (0.574, 1.303)	0.865 (0.574, 1.304)
ITGBL1	1 (Reference)	1 (Reference)	1 (Reference)
Q2	1.125 (0.651, 1.941)	1.127 (0.653, 1.946)	1.122 (0.650, 1.938)
Q3	1.400 (0.829, 2.364)	1.332 (0.785, 2.261)	1.319 (0.777, 2.240)
Q4	**2.159 (1.323, 3.524)**	**1.738 (1.049, 2.877)**	**1.684 (1.019, 2.784)**

Q1 (lowest, < 25th percentile), Q2 (25th to < 50th percentile), Q3 (50th to < 75th percentile), and Q4 (highest, ⩾ 75th percentile); CI: confidence interval; ITG, Integrin.
