# Supplementary material for: The Role of Integrin Family in Atherosclerotic Cardiovascular Disease: A Prospective Cohort Study and Mendelian Randomization Analysis
Source: Biomedicines. 2026 Aug 15;14(8):1836. doi: 10.3390/biomedicines14081836 (PMC13509564; doi:10.3390/biomedicines14081836)
Supplement: Supplementary file 1 [file biomedicines-14-01836-s001.zip › TableS12.pdf]

Table S12. HR (95% CI) of IS mortality by integrin levels.

Integrin	Hazard Ratio (95% CI)		
	Model 1	Model 2	Model 3
ITGA11	1 (Reference)	1 (Reference)	1 (Reference)
Q2	0.738 (0.439, 1.241)	0.767 (0.456, 1.292)	0.766 (0.453, 1.297)
Q3	**0.528 (0.293, 0.949)**	0.562 (0.309, 1.023)	0.556 (0.310, 0.999)
Q4	**0.382 (0.201, 0.726)**	**0.417 (0.214, 0.814)**	**0.410 (0.208, 0.809)**
ITGA2	1 (Reference)	1 (Reference)	1 (Reference)
Q2	0.581 (0.331, 1.022)	0.607 (0.342, 1.078)	0.618 (0.347, 1.101)
Q3	0.582 (0.334, 1.016)	0.623 (0.359, 1.083)	0.642 (0.368, 1.120)
Q4	**0.432 (0.238, 0.784)**	**0.468 (0.257, 0.851)**	**0.494 (0.270, 0.904)**
ITGA5	1 (Reference)	1 (Reference)	1 (Reference)
Q2	0.669 (0.339, 1.321)	0.646 (0.328, 1.274)	0.660 (0.335, 1.298)
Q3	1.287 (0.719, 2.304)	1.232 (0.680, 2.231)	1.277 (0.706, 2.307)
Q4	0.948 (0.517, 1.739)	0.841 (0.444, 1.595)	0.897 (0.471, 1.711)
ITGA6	1 (Reference)	1 (Reference)	1 (Reference)
Q2	**0.523 (0.291, 0.942)**	**0.533 (0.296, 0.962)**	**0.539 (0.299, 0.970)**
Q3	0.599 (0.337, 1.068)	0.617 (0.346, 1.099)	0.630 (0.353, 1.126)
Q4	0.739 (0.425, 1.286)	0.765 (0.440, 1.332)	0.798 (0.458, 1.388)
ITGAL	1 (Reference)	1 (Reference)	1 (Reference)
Q2	0.705 (0.388, 1.282)	0.695 (0.383, 1.261)	0.702 (0.386, 1.275)
Q3	0.642 (0.353, 1.165)	0.615 (0.337, 1.123)	0.630 (0.345, 1.150)
Q4	0.742 (0.427, 1.288)	0.694 (0.401, 1.200)	0.719 (0.414, 1.246)
ITGAM	1 (Reference)	1 (Reference)	1 (Reference)
Q2	0.697 (0.412, 1.179)	0.706 (0.416, 1.199)	0.713 (0.419, 1.212)
Q3	**0.384 (0.203, 0.729)**	**0.393 (0.206, 0.749)**	**0.393 (0.206, 0.750)**
Q4	**0.524 (0.295, 0.931)**	**0.523 (0.291, 0.940)**	**0.528 (0.296, 0.939)**
ITGAV	1 (Reference)	1 (Reference)	1 (Reference)
Q2	0.623 (0.363, 1.067)	0.646 (0.373, 1.118)	0.645 (0.374, 1.113)
Q3	**0.450 (0.253, 0.801)**	**0.479 (0.263, 0.873)**	**0.474 (0.264, 0.854)**
Q4	**0.305 (0.165, 0.562)**	**0.331 (0.168, 0.653)**	**0.328 (0.165, 0.652)**
ITGAX	1 (Reference)	1 (Reference)	1 (Reference)
Q2	0.857 (0.471, 1.561)	0.840 (0.460, 1.535)	0.838 (0.459, 1.530)
Q3	0.770 (0.416, 1.426)	0.761 (0.410, 1.412)	0.759 (0.409, 1.408)
Q4	1.133 (0.650, 1.977)	1.110 (0.633, 1.945)	1.106 (0.631, 1.939)
ITGB1	1 (Reference)	1 (Reference)	1 (Reference)
Q2	1.253 (0.701, 2.241)	1.304 (0.730, 2.331)	1.340 (0.749, 2.397)
Q3	0.668 (0.343, 1.301)	0.713 (0.367, 1.387)	0.739 (0.380, 1.436)
Q4	0.937 (0.511, 1.716)	1.026 (0.554, 1.904)	1.087 (0.574, 2.056)
ITGB1BP1	1 (Reference)	1 (Reference)	1 (Reference)
Q2	0.745 (0.396, 1.404)	0.751 (0.399, 1.413)	0.755 (0.402, 1.418)
Q3	1.154 (0.654, 2.037)	1.160 (0.658, 2.048)	1.178 (0.669, 2.074)
Q4	1.038 (0.578, 1.863)	1.035 (0.577, 1.857)	1.057 (0.590, 1.893)

ITGB1BP2	1 (Reference)	1 (Reference)	1 (Reference)
Q2	0.784 (0.437, 1.407)	0.781 (0.435, 1.402)	0.794 (0.442, 1.423)
Q3	0.761 (0.420, 1.378)	0.750 (0.414, 1.358)	0.772 (0.426, 1.399)
Q4	0.996 (0.566, 1.752)	0.962 (0.546, 1.696)	1.011 (0.575, 1.779)
ITGB2	1 (Reference)	1 (Reference)	1 (Reference)
Q2	0.825 (0.485, 1.404)	0.830 (0.487, 1.415)	0.842 (0.493, 1.439)
Q3	**0.358 (0.182, 0.702)**	**0.364 (0.185, 0.715)**	**0.369 (0.188, 0.726)**
Q4	0.631 (0.361, 1.104)	0.620 (0.352, 1.090)	0.631 (0.358, 1.114)
ITGB5	1 (Reference)	1 (Reference)	1 (Reference)
Q2	1.126 (0.638, 1.989)	1.185 (0.669, 2.102)	1.188 (0.669, 2.107)
Q3	0.643 (0.341, 1.211)	0.677 (0.360, 1.273)	0.681 (0.362, 1.283)
Q4	0.742 (0.407, 1.355)	0.790 (0.434, 1.437)	0.818 (0.448, 1.493)
ITGB6	1 (Reference)	1 (Reference)	1 (Reference)
Q2	0.700 (0.380, 1.289)	0.712 (0.387, 1.311)	0.715 (0.389, 1.316)
Q3	0.923 (0.521, 1.635)	0.936 (0.529, 1.655)	0.937 (0.530, 1.656)
Q4	0.871 (0.491, 1.545)	0.874 (0.492, 1.554)	0.882 (0.493, 1.577)
ITGB7	1 (Reference)	1 (Reference)	1 (Reference)
Q2	0.645 (0.335, 1.241)	0.649 (0.337, 1.250)	0.658 (0.342, 1.267)
Q3	1.192 (0.679, 2.091)	1.177 (0.670, 2.068)	1.194 (0.679, 2.101)
Q4	1.023 (0.573, 1.826)	0.996 (0.559, 1.776)	1.010 (0.566, 1.803)
ITGBL1	1 (Reference)	1 (Reference)	1 (Reference)
Q2	0.943 (0.500, 1.776)	0.970 (0.514, 1.830)	0.989 (0.524, 1.867)
Q3	**0.407 (0.192, 0.863)**	**0.408 (0.191, 0.869)**	**0.417 (0.196, 0.885)**
Q4	1.127 (0.621, 2.046)	1.074 (0.583, 1.980)	1.122 (0.607, 2.073)

Q1 (lowest, < 25th percentile), Q2 (25th to < 50th percentile), Q3 (50th to < 75th percentile), and Q4 (highest, ⩾ 75th percentile); CI: confidence interval; ITG, Integrin.
