# Supplementary material for: The Role of Integrin Family in Atherosclerotic Cardiovascular Disease: A Prospective Cohort Study and Mendelian Randomization Analysis
Source: Biomedicines. 2026 Aug 15;14(8):1836. doi: 10.3390/biomedicines14081836 (PMC13509564; doi:10.3390/biomedicines14081836)
Supplement: Supplementary file 1 [file biomedicines-14-01836-s001.zip › TableS13.pdf]

**Table S13. HR (95% CI) of PAD mortality by integrin levels.**

| | Hazard Ratio (95% CI) | | |
|---|---|---|---|
| Integrin | Model 1 | Model 2 | Model 3 |
| ITGA11 | 1 (Reference) | 1 (Reference) | 1 (Reference) |
| Q2 | 0.977 (0.198, 4.815) | 0.948 (0.177, 5.075) | 0.861 (0.170, 4.364) |
| Q3 | 1.371 (0.311, 6.042) | 1.312 (0.314, 5.478) | 1.008 (0.234, 4.353) |
| Q4 | **0.000 (0.000, 0.000)** | **0.000 (0.000, 0.000)** | **0.000 (0.000, 0.000)** |
| ITGA2 | 1 (Reference) | 1 (Reference) | 1 (Reference) |
| Q2 | 1.505 (0.250, 9.073) | 1.568 (0.281, 8.755) | 1.425 (0.259, 7.845) |
| Q3 | 0.963 (0.135, 6.886) | 0.989 (0.144, 6.782) | 0.837 (0.120, 5.833) |
| Q4 | 1.352 (0.224, 8.167) | 1.299 (0.199, 8.470) | 0.990 (0.148, 6.618) |
| ITGA5 | 1 (Reference) | 1 (Reference) | 1 (Reference) |
| Q2 | 0.269 (0.027, 2.634) | 0.267 (0.026, 2.751) | 0.267 (0.025, 2.803) |
| Q3 | 0.525 (0.085, 3.228) | 0.542 (0.083, 3.553) | 0.553 (0.087, 3.523) |
| Q4 | 0.919 (0.182, 4.628) | 0.921 (0.149, 5.692) | 0.894 (0.126, 6.336) |
| ITGA6 | 1 (Reference) | 1 (Reference) | 1 (Reference) |
| Q2 | 0.995 (0.142, 6.949) | 1.037 (0.145, 7.418) | 1.056 (0.150, 7.432) |
| Q3 | 1.585 (0.262, 9.571) | 1.692 (0.272, 10.535) | 1.659 (0.256, 10.761) |
| Q4 | 1.677 (0.287, 9.804) | 1.778 (0.284, 11.131) | 1.838 (0.267, 12.631) |
| ITGAL | 1 (Reference) | 1 (Reference) | 1 (Reference) |
| Q2 | 1.404 (0.236, 8.364) | 1.363 (0.223, 8.330) | 1.376 (0.233, 8.110) |
| Q3 | 1.711 (0.320, 9.135) | 1.624 (0.304, 8.661) | 1.653 (0.316, 8.660) |
| Q4 | 0.382 (0.033, 4.347) | 0.355 (0.032, 3.956) | 0.336 (0.030, 3.708) |
| ITGAM | 1 (Reference) | 1 (Reference) | 1 (Reference) |
| Q2 | 0.960 (0.196, 4.712) | 0.946 (0.186, 4.825) | 0.875 (0.171, 4.475) |
| Q3 | 0.651 (0.110, 3.843) | 0.636 (0.110, 3.692) | 0.544 (0.092, 3.222) |
| Q4 | 0.645 (0.109, 3.824) | 0.584 (0.114, 3.001) | 0.478 (0.090, 2.535) |
| ITGAV | 1 (Reference) | 1 (Reference) | 1 (Reference) |
| Q2 | 1.710 (0.311, 9.417) | 1.724 (0.336, 8.848) | 1.386 (0.228, 8.417) |
| Q3 | 0.749 (0.102, 5.494) | 0.739 (0.100, 5.479) | 0.607 (0.084, 4.408) |
| Q4 | 0.649 (0.092, 4.589) | 0.619 (0.078, 4.907) | 0.455 (0.068, 3.039) |
| ITGAX | 1 (Reference) | 1 (Reference) | 1 (Reference) |
| Q2 | 0.988 (0.062, 15.819) | 0.946 (0.060, 14.879) | 0.957 (0.064, 14.394) |
| Q3 | 1.978 (0.179, 21.910) | 1.898 (0.179, 20.131) | 1.943 (0.186, 20.341) |
| Q4 | 5.832 (0.700, 48.588) | 5.563 (0.680, 45.532) | 5.430 (0.680, 43.367) |
| ITGB1 | 1 (Reference) | 1 (Reference) | 1 (Reference) |
| Q2 | 0.285 (0.030, 2.701) | 0.293 (0.032, 2.674) | 0.259 (0.030, 2.225) |
| Q3 | 0.810 (0.155, 4.240) | 0.814 (0.163, 4.058) | 0.657 (0.116, 3.731) |
| Q4 | 0.728 (0.138, 3.830) | 0.694 (0.124, 3.892) | 0.488 (0.072, 3.279) |
| ITGB1BP1 | 1 (Reference) | 1 (Reference) | 1 (Reference) |
| Q2 | 0.320 (0.033, 3.069) | 0.327 (0.035, 3.099) | 0.361 (0.034, 3.857) |
| Q3 | 0.641 (0.106, 3.881) | 0.648 (0.107, 3.937) | 0.712 (0.116, 4.385) |
| Q4 | 1.318 (0.297, 5.851) | 1.357 (0.310, 5.946) | 1.492 (0.302, 7.370) |

| | | | |
|---|---|---|---|
| ITGB1BP2 | 1 (Reference) | 1 (Reference) | 1 (Reference) |
| Q2 | 1.015 (0.064, 16.177) | 1.007 (0.063, 15.965) | 1.014 (0.063, 16.183) |
| Q3 | 1.024 (0.064, 16.427) | 1.003 (0.062, 16.364) | 1.023 (0.063, 16.489) |
| Q4 | 7.916 (0.961, 65.221) | 7.481 (0.869, 64.403) | 7.442 (0.806, 68.754) |
| ITGB2 | 1 (Reference) | 1 (Reference) | 1 (Reference) |
| Q2 | 0.356 (0.069, 1.838) | 0.352 (0.069, 1.798) | 0.328 (0.065, 1.657) |
| Q3 | 0.336 (0.065, 1.744) | 0.335 (0.071, 1.581) | 0.311 (0.066, 1.465) |
| Q4 | 0.163 (0.019, 1.386) | 0.147 (0.018, 1.223) | 0.123 (0.013, 1.189) |
| ITGB5 | 1 (Reference) | 1 (Reference) | 1 (Reference) |
| Q2 | 1.214 (0.205, 7.181) | 1.220 (0.222, 6.715) | 1.123 (0.200, 6.300) |
| Q3 | 0.362 (0.034, 3.866) | 0.344 (0.032, 3.703) | 0.271 (0.027, 2.703) |
| Q4 | 1.380 (0.234, 8.144) | 1.318 (0.220, 7.892) | 1.052 (0.175, 6.336) |
| ITGB6 | 1 (Reference) | 1 (Reference) | 1 (Reference) |
| Q2 | 0.933 (0.192, 4.535) | 0.972 (0.199, 4.753) | 0.974 (0.202, 4.692) |
| Q3 | 0.970 (0.196, 4.799) | 1.025 (0.210, 5.004) | 1.005 (0.209, 4.828) |
| Q4 | 0.304 (0.031, 2.961) | 0.327 (0.034, 3.188) | 0.342 (0.036, 3.280) |
| ITGB7 | 1 (Reference) | 1 (Reference) | 1 (Reference) |
| Q2 | 0.629 (0.105, 3.772) | 0.602 (0.098, 3.678) | 0.598 (0.096, 3.717) |
| Q3 | 0.643 (0.107, 3.879) | 0.584 (0.099, 3.428) | 0.546 (0.086, 3.483) |
| Q4 | 0.942 (0.190, 4.672) | 0.821 (0.164, 4.096) | 0.813 (0.166, 3.998) |
| ITGBL1 | 1 (Reference) | 1 (Reference) | 1 (Reference) |
| Q2 | 1.106 (0.179, 6.828) | 1.235 (0.195, 7.824) | 1.248 (0.193, 8.046) |
| Q3 | **0.000 (0.000, 0.000)** | **0.000 (0.000, 0.000)** | **0.000 (0.000, 0.000)** |
| Q4 | 1.306 (0.203, 8.393) | 1.400 (0.206, 9.504) | 1.438 (0.216, 9.558) |

Q1 (lowest, < 25th percentile), Q2 (25th to < 50th percentile), Q3 (50th to < 75th percentile), and Q4 (highest, ⩾ 75th percentile); CI: confidence interval; ITG, Integrin.
