# Supplementary material for: The Role of Integrin Family in Atherosclerotic Cardiovascular Disease: A Prospective Cohort Study and Mendelian Randomization Analysis
Source: Biomedicines. 2026 Aug 15;14(8):1836. doi: 10.3390/biomedicines14081836 (PMC13509564; doi:10.3390/biomedicines14081836)
Supplement: Supplementary file 1 [file biomedicines-14-01836-s001.zip › TableS14.pdf]

Table S14. Colocalization analysis of the association of integrin with ASCVD.

Disease	Integrin	PP.H0	PP.H1	PP.H2	PP.H3	PP.H4
CHD	ITGA11	< 0.001	0.870	< 0.001	0.034	0.096
	ITGA2	< 0.001	0.963	< 0.001	0.033	0.004
	ITGA6	< 0.001	0.945	< 0.001	0.051	0.004
	ITGAL	< 0.001	0.975	< 0.001	0.022	0.004
	ITGAM	< 0.001	0.955	< 0.001	0.028	0.018
	ITGAV	< 0.001	0.406	< 0.001	0.023	**0.571**
	ITGB2	< 0.001	0.907	< 0.001	0.089	0.004
	ITGB5	< 0.001	0.935	< 0.001	0.049	0.015
	ITGB6	< 0.001	0.952	< 0.001	0.043	0.005
	ITGB7	< 0.001	0.407	< 0.001	0.152	0.441
	ITGBL1	< 0.001	0.959	< 0.001	0.037	0.004
IS	ITGA11	< 0.001	0.975	< 0.001	0.019	0.006
	ITGA2	< 0.001	0.957	< 0.001	0.037	0.007
	ITGA6	< 0.001	0.967	< 0.001	0.029	0.005
	ITGAL	< 0.001	0.951	< 0.001	0.023	0.026
	ITGAM	< 0.001	0.982	< 0.001	0.014	0.004
	ITGAV	< 0.001	0.161	< 0.001	0.019	**0.820**
	ITGB2	< 0.001	0.958	< 0.001	0.034	0.008
	ITGB5	< 0.001	0.792	< 0.001	0.093	0.115
	ITGB6	< 0.001	0.949	< 0.001	0.047	0.004
	ITGB7	< 0.001	0.968	< 0.001	0.026	0.006
	ITGBL1	< 0.001	0.928	< 0.001	0.045	0.027
PAD	ITGA11	< 0.001	0.932	< 0.001	0.045	0.023
	ITGA2	< 0.001	0.830	< 0.001	0.127	0.043
	ITGA6	< 0.001	0.936	< 0.001	0.050	0.014
	ITGAL	< 0.001	0.950	< 0.001	0.037	0.013
	ITGAM	< 0.001	0.881	< 0.001	0.047	0.071
	ITGAV	< 0.001	0.944	< 0.001	0.028	0.028
	ITGB2	< 0.001	0.893	< 0.001	0.079	0.029
	ITGB5	< 0.001	0.810	< 0.001	0.074	0.117
	ITGB6	< 0.001	0.936	< 0.001	0.042	0.021
	ITGB7	< 0.001	0.928	< 0.001	0.038	0.034
	ITGBL1	< 0.001	0.928	< 0.001	0.059	0.014

ASCVD, atherosclerotic cardiovascular disease; CHD, coronary heart disease; IS, ischemic stroke; PAD, peripheral artery disease; ITG, integrin. H_0: neither trait is genetically associated; H_1: only ITG is associated; H_2: only ASCVD is associated; H_3: both traits are associated but through distinct causal variants; H_4: both traits share a common causal variant. Strong support of colocalization was defined if the PP.H4 > 0.8, and medium support of colocalization was defined as 0.5 < PP.H4 ≤ 0.8.
