# Supplementary material for: The Role of Integrin Family in Atherosclerotic Cardiovascular Disease: A Prospective Cohort Study and Mendelian Randomization Analysis
Source: Biomedicines. 2026 Aug 15;14(8):1836. doi: 10.3390/biomedicines14081836 (PMC13509564; doi:10.3390/biomedicines14081836)
Supplement: Supplementary file 1 [file biomedicines-14-01836-s001.zip › TableS15.pdf]

Table S15. HR (95% CI) of incident ASCVD by integrin levels[†].

Integrin	Hazard Ratio (95% CI)		
	Model 1	Model 2	Model 3
ITGA11	1 (Reference)	1 (Reference)	1 (Reference)
Q2	**0.764 (0.686, 0.851)**	**0.841 (0.755, 0.936)**	**0.865 (0.776, 0.964)**
Q3	**0.628 (0.561, 0.702)**	**0.741 (0.661, 0.831)**	**0.789 (0.703, 0.885)**
Q4	**0.501 (0.445, 0.565)**	**0.638 (0.565, 0.721)**	**0.702 (0.620, 0.795)**
ITGA2	1 (Reference)	1 (Reference)	1 (Reference)
Q2	**0.765 (0.686, 0.854)**	**0.827 (0.741, 0.923)**	**0.850 (0.761, 0.949)**
Q3	**0.664 (0.593, 0.743)**	**0.748 (0.667, 0.838)**	**0.785 (0.700, 0.881)**
Q4	**0.558 (0.497, 0.626)**	**0.663 (0.589, 0.746)**	**0.711 (0.631, 0.803)**
ITGA5	1 (Reference)	1 (Reference)	1 (Reference)
Q2	**1.163 (1.020, 1.325)**	1.083 (0.950, 1.235)	1.078 (0.946, 1.229)
Q3	**1.295 (1.141, 1.470)**	**1.159 (1.020, 1.316)**	**1.150 (1.012, 1.307)**
Q4	**1.500 (1.327, 1.696)**	**1.184 (1.044, 1.342)**	**1.176 (1.036, 1.334)**
ITGA6	1 (Reference)	1 (Reference)	1 (Reference)
Q2	1.026 (0.917, 1.149)	1.038 (0.928, 1.162)	1.034 (0.924, 1.157)
Q3	0.982 (0.875, 1.102)	1.004 (0.894, 1.127)	0.991 (0.883, 1.113)
Q4	0.912 (0.810, 1.026)	0.932 (0.828, 1.049)	0.923 (0.820, 1.039)
ITGAL	1 (Reference)	1 (Reference)	1 (Reference)
Q2	1.091 (0.968, 1.230)	1.064 (0.944, 1.199)	1.060 (0.941, 1.195)
Q3	0.991 (0.878, 1.119)	0.929 (0.823, 1.048)	0.921 (0.816, 1.040)
Q4	1.105 (0.983, 1.241)	0.989 (0.880, 1.112)	0.976 (0.868, 1.098)
ITGAM	1 (Reference)	1 (Reference)	1 (Reference)
Q2	**0.807 (0.722, 0.901)**	**0.831 (0.744, 0.928)**	**0.853 (0.764, 0.953)**
Q3	**0.727 (0.649, 0.814)**	**0.765 (0.683, 0.858)**	**0.796 (0.710, 0.892)**
Q4	**0.638 (0.568, 0.717)**	**0.676 (0.601, 0.761)**	**0.725 (0.644, 0.817)**
ITGAV	1 (Reference)	1 (Reference)	1 (Reference)
Q2	**0.763 (0.683, 0.853)**	**0.840 (0.751, 0.939)**	**0.872 (0.780, 0.975)**
Q3	**0.594 (0.530, 0.666)**	**0.698 (0.622, 0.784)**	**0.745 (0.663, 0.838)**
Q4	**0.467 (0.415, 0.526)**	**0.602 (0.533, 0.681)**	**0.670 (0.591, 0.759)**
ITGAX	1 (Reference)	1 (Reference)	1 (Reference)
Q2	1.050 (0.933, 1.181)	1.030 (0.915, 1.159)	1.032 (0.917, 1.161)
Q3	1.037 (0.922, 1.167)	1.022 (0.908, 1.150)	1.018 (0.905, 1.146)
Q4	1.137 (1.013, 1.276)	1.117 (0.995, 1.254)	1.112 (0.990, 1.248)
ITGB1	1 (Reference)	1 (Reference)	1 (Reference)
Q2	**0.818 (0.731, 0.914)**	**0.858 (0.767, 0.960)**	**0.879 (0.786, 0.984)**
Q3	**0.671 (0.597, 0.753)**	**0.736 (0.655, 0.826)**	**0.777 (0.691, 0.874)**
Q4	**0.606 (0.539, 0.681)**	**0.713 (0.634, 0.802)**	**0.778 (0.690, 0.878)**
ITGB1BP1	1 (Reference)	1 (Reference)	1 (Reference)
Q2	1.014 (0.902, 1.140)	1.022 (0.909, 1.149)	1.019 (0.906, 1.145)
Q3	1.035 (0.921, 1.163)	1.044 (0.929, 1.174)	1.038 (0.923, 1.166)

Q4	1.066 (0.949, 1.197)	1.058 (0.942, 1.189)	1.047 (0.932, 1.176)
ITGB1BP2	1 (Reference)	1 (Reference)	1 (Reference)
Q2	1.028 (0.915, 1.155)	1.013 (0.902, 1.139)	1.012 (0.900, 1.137)
Q3	1.075 (0.958, 1.208)	1.050 (0.935, 1.179)	1.052 (0.937, 1.181)
Q4	1.099 (0.978, 1.235)	1.057 (0.941, 1.188)	1.047 (0.931, 1.177)
ITGB2	1 (Reference)	1 (Reference)	1 (Reference)
Q2	**0.815 (0.728, 0.913)**	**0.824 (0.736, 0.923)**	**0.836 (0.746, 0.936)**
Q3	**0.706 (0.628, 0.794)**	**0.724 (0.644, 0.814)**	**0.741 (0.659, 0.833)**
Q4	**0.743 (0.663, 0.833)**	**0.742 (0.662, 0.832)**	**0.774 (0.690, 0.868)**
ITGB5	1 (Reference)	1 (Reference)	1 (Reference)
Q2	**0.852 (0.757, 0.958)**	0.916 (0.814, 1.031)	0.945 (0.840, 1.064)
Q3	**0.796 (0.708, 0.896)**	**0.885 (0.786, 0.996)**	0.936 (0.831, 1.054)
Q4	**0.795 (0.707, 0.893)**	0.897 (0.798, 1.009)	0.960 (0.852, 1.081)
ITGB6	1 (Reference)	1 (Reference)	1 (Reference)
Q2	0.945 (0.838, 1.065)	0.967 (0.857, 1.090)	0.960 (0.852, 1.083)
Q3	**1.162 (1.035, 1.305)**	**1.187 (1.057, 1.333)**	**1.167 (1.039, 1.310)**
Q4	1.107 (0.985, 1.244)	1.115 (0.993, 1.253)	1.080 (0.961, 1.214)
ITGB7	1 (Reference)	1 (Reference)	1 (Reference)
Q2	**0.862 (0.766, 0.972)**	**0.877 (0.778, 0.988)**	**0.886 (0.787, 0.999)**
Q3	1.000 (0.890, 1.122)	1.000 (0.890, 1.123)	1.013 (0.902, 1.137)
Q4	1.045 (0.933, 1.172)	1.041 (0.928, 1.166)	1.049 (0.935, 1.175)
ITGBL1	1 (Reference)	1 (Reference)	1 (Reference)
Q2	0.971 (0.851, 1.108)	0.975 (0.854, 1.113)	0.971 (0.850, 1.108)
Q3	1.048 (0.921, 1.191)	1.028 (0.903, 1.169)	1.019 (0.896, 1.159)
Q4	**1.323 (1.170, 1.496)**	**1.153 (1.018, 1.306)**	1.127 (0.994, 1.277)

Q1 (lowest, < 25th percentile), Q2 (25th to < 50th percentile), Q3 (50th to < 75th percentile), and Q4 (highest, ⩾ 75th percentile); CI: confidence interval; ITG, Integrin. †The analysis excluded participants who developed ASCVD within the first 2 years of follow-up.
