# Supplementary material for: The Role of Integrin Family in Atherosclerotic Cardiovascular Disease: A Prospective Cohort Study and Mendelian Randomization Analysis
Source: Biomedicines. 2026 Aug 15;14(8):1836. doi: 10.3390/biomedicines14081836 (PMC13509564; doi:10.3390/biomedicines14081836)
Supplement: Supplementary file 1 [file biomedicines-14-01836-s001.zip › TableS16.pdf]

Table S16. HR (95% CI) of incident CHD by integrin levels†.

	Hazard Ratio (95% CI)		
Integrin	Model 1	Model 2	Model 3
ITGA11	1 (Reference)	1 (Reference)	1 (Reference)
Q2	**0.714 (0.623, 0.817)**	**0.797 (0.696, 0.914)**	**0.833 (0.727, 0.955)**
Q3	**0.587 (0.509, 0.678)**	**0.711 (0.615, 0.822)**	**0.779 (0.673, 0.902)**
Q4	**0.480 (0.413, 0.557)**	**0.633 (0.542, 0.739)**	**0.728 (0.622, 0.852)**
ITGA2	1 (Reference)	1 (Reference)	1 (Reference)
Q2	**0.731 (0.637, 0.840)**	**0.798 (0.695, 0.916)**	**0.833 (0.724, 0.957)**
Q3	**0.654 (0.568, 0.753)**	**0.749 (0.650, 0.863)**	**0.808 (0.700, 0.932)**
Q4	**0.502 (0.432, 0.583)**	**0.614 (0.527, 0.714)**	**0.686 (0.588, 0.802)**
ITGA5	1 (Reference)	1 (Reference)	1 (Reference)
Q2	**1.204 (1.020, 1.420)**	1.105 (0.936, 1.304)	1.099 (0.932, 1.297)
Q3	**1.286 (1.095, 1.512)**	1.123 (0.955, 1.321)	1.113 (0.946, 1.310)
Q4	**1.528 (1.308, 1.785)**	1.152 (0.983, 1.352)	1.145 (0.975, 1.345)
ITGA6	1 (Reference)	1 (Reference)	1 (Reference)
Q2	1.026 (0.890, 1.182)	1.037 (0.900, 1.196)	1.033 (0.896, 1.190)
Q3	0.975 (0.843, 1.128)	0.998 (0.862, 1.154)	0.984 (0.850, 1.138)
Q4	0.881 (0.758, 1.025)	0.904 (0.777, 1.050)	0.896 (0.770, 1.042)
ITGAL	1 (Reference)	1 (Reference)	1 (Reference)
Q2	1.137 (0.977, 1.324)	1.104 (0.948, 1.285)	1.099 (0.944, 1.280)
Q3	1.009 (0.865, 1.178)	0.938 (0.803, 1.095)	0.929 (0.796, 1.085)
Q4	1.152 (0.993, 1.336)	1.015 (0.875, 1.178)	1.001 (0.862, 1.162)
ITGAM	1 (Reference)	1 (Reference)	1 (Reference)
Q2	**0.787 (0.685, 0.903)**	**0.812 (0.707, 0.932)**	**0.842 (0.733, 0.967)**
Q3	**0.687 (0.595, 0.793)**	**0.728 (0.631, 0.840)**	**0.770 (0.667, 0.890)**
Q4	**0.593 (0.511, 0.688)**	**0.631 (0.543, 0.734)**	**0.697 (0.599, 0.811)**
ITGAV	1 (Reference)	1 (Reference)	1 (Reference)
Q2	**0.765 (0.666, 0.878)**	**0.851 (0.741, 0.978)**	0.902 (0.785, 1.037)
Q3	**0.561 (0.485, 0.649)**	**0.674 (0.581, 0.781)**	**0.743 (0.640, 0.862)**
Q4	**0.454 (0.391, 0.528)**	**0.609 (0.521, 0.711)**	**0.715 (0.610, 0.838)**
ITGAX	1 (Reference)	1 (Reference)	1 (Reference)
Q2	1.088 (0.938, 1.262)	1.068 (0.921, 1.239)	1.069 (0.922, 1.240)
Q3	1.030 (0.887, 1.197)	1.014 (0.873, 1.178)	1.010 (0.870, 1.174)
Q4	1.112 (0.959, 1.288)	1.090 (0.940, 1.263)	1.082 (0.934, 1.254)
ITGB1	1 (Reference)	1 (Reference)	1 (Reference)
Q2	**0.863 (0.751, 0.992)**	0.912 (0.794, 1.048)	0.945 (0.822, 1.087)
Q3	**0.650 (0.561, 0.753)**	**0.721 (0.622, 0.836)**	**0.781 (0.673, 0.906)**
Q4	**0.560 (0.482, 0.651)**	**0.672 (0.577, 0.782)**	**0.764 (0.654, 0.891)**
ITGB1BP1	1 (Reference)	1 (Reference)	1 (Reference)
Q2	1.039 (0.895, 1.205)	1.051 (0.906, 1.219)	1.047 (0.902, 1.214)
Q3	1.029 (0.887, 1.195)	1.041 (0.897, 1.208)	1.033 (0.890, 1.199)

Q4	1.097 (0.947, 1.271)	1.091 (0.942, 1.264)	1.076 (0.929, 1.247)
ITGB1BP2	1 (Reference)	1 (Reference)	1 (Reference)
Q2	0.998 (0.861, 1.157)	0.981 (0.846, 1.137)	0.980 (0.845, 1.136)
Q3	1.080 (0.934, 1.250)	1.053 (0.911, 1.218)	1.057 (0.914, 1.223)
Q4	1.053 (0.908, 1.220)	1.009 (0.870, 1.171)	1.000 (0.861, 1.160)
ITGB2	1 (Reference)	1 (Reference)	1 (Reference)
Q2	**0.866 (0.751, 0.999)**	0.875 (0.759, 1.009)	0.894 (0.775, 1.032)
Q3	**0.729 (0.628, 0.846)**	**0.747 (0.643, 0.867)**	**0.774 (0.666, 0.898)**
Q4	**0.757 (0.655, 0.875)**	**0.753 (0.651, 0.872)**	**0.800 (0.691, 0.926)**
ITGB5	1 (Reference)	1 (Reference)	1 (Reference)
Q2	0.907 (0.784, 1.050)	0.986 (0.851, 1.141)	1.029 (0.889, 1.192)
Q3	**0.763 (0.657, 0.887)**	0.861 (0.740, 1.001)	0.929 (0.799, 1.081)
Q4	**0.759 (0.654, 0.882)**	0.872 (0.750, 1.013)	0.959 (0.824, 1.116)
ITGB6	1 (Reference)	1 (Reference)	1 (Reference)
Q2	0.914 (0.782, 1.067)	0.939 (0.804, 1.097)	0.930 (0.796, 1.087)
Q3	**1.245 (1.075, 1.442)**	**1.275 (1.101, 1.477)**	**1.243 (1.073, 1.439)**
Q4	**1.186 (1.024, 1.375)**	**1.195 (1.031, 1.385)**	1.142 (0.985, 1.324)
ITGB7	1 (Reference)	1 (Reference)	1 (Reference)
Q2	**0.816 (0.703, 0.947)**	**0.831 (0.716, 0.964)**	**0.845 (0.728, 0.981)**
Q3	0.886 (0.765, 1.027)	0.888 (0.766, 1.028)	0.904 (0.780, 1.047)
Q4	0.988 (0.857, 1.138)	0.980 (0.850, 1.130)	0.990 (0.858, 1.141)
ITGBL1	1 (Reference)	1 (Reference)	1 (Reference)
Q2	0.918 (0.778, 1.084)	0.915 (0.775, 1.080)	0.912 (0.772, 1.077)
Q3	0.986 (0.839, 1.158)	0.953 (0.811, 1.120)	0.946 (0.805, 1.112)
Q4	**1.264 (1.084, 1.473)**	1.066 (0.913, 1.246)	1.035 (0.885, 1.211)

Q1 (lowest, < 25th percentile), Q2 (25th to < 50th percentile), Q3 (50th to < 75th percentile), and Q4 (highest, ⩾ 75th percentile); CI: confidence interval; ITG, Integrin. †The analysis excluded participants who developed CHD within the first 2 years of follow-up.
