# Supplementary material for: The Role of Integrin Family in Atherosclerotic Cardiovascular Disease: A Prospective Cohort Study and Mendelian Randomization Analysis
Source: Biomedicines. 2026 Aug 15;14(8):1836. doi: 10.3390/biomedicines14081836 (PMC13509564; doi:10.3390/biomedicines14081836)
Supplement: Supplementary file 1 [file biomedicines-14-01836-s001.zip › TableS17.pdf]

Table S17. HR (95% CI) of incident IS by integrin levels[†].

Integrin	Hazard Ratio (95% CI)		
	Model 1	Model 2	Model 3
ITGA11	1 (Reference)	1 (Reference)	1 (Reference)
Q2	**0.724 (0.614, 0.854)**	**0.793 (0.672, 0.936)**	**0.827 (0.701, 0.977)**
Q3	**0.591 (0.496, 0.703)**	**0.690 (0.578, 0.825)**	**0.754 (0.630, 0.902)**
Q4	**0.497 (0.414, 0.596)**	**0.624 (0.517, 0.753)**	**0.715 (0.591, 0.865)**
ITGA2	1 (Reference)	1 (Reference)	1 (Reference)
Q2	**0.749 (0.631, 0.888)**	**0.806 (0.679, 0.957)**	**0.839 (0.707, 0.997)**
Q3	**0.705 (0.593, 0.837)**	**0.790 (0.664, 0.940)**	0.847 (0.711, 1.010)
Q4	**0.574 (0.480, 0.687)**	**0.680 (0.567, 0.817)**	**0.752 (0.624, 0.907)**
ITGA5	1 (Reference)	1 (Reference)	1 (Reference)
Q2	1.186 (0.967, 1.454)	1.103 (0.899, 1.353)	1.091 (0.889, 1.339)
Q3	**1.454 (1.196, 1.767)**	**1.294 (1.064, 1.574)**	**1.274 (1.047, 1.551)**
Q4	**1.580 (1.305, 1.914)**	**1.245 (1.023, 1.515)**	**1.220 (1.001, 1.487)**
ITGA6	1 (Reference)	1 (Reference)	1 (Reference)
Q2	1.124 (0.944, 1.338)	1.132 (0.951, 1.348)	1.126 (0.946, 1.340)
Q3	0.985 (0.821, 1.182)	1.001 (0.835, 1.201)	0.984 (0.820, 1.181)
Q4	0.990 (0.824, 1.188)	1.007 (0.838, 1.209)	0.991 (0.825, 1.191)
ITGAL	1 (Reference)	1 (Reference)	1 (Reference)
Q2	**1.216 (1.006, 1.468)**	1.185 (0.981, 1.431)	1.179 (0.976, 1.424)
Q3	1.124 (0.929, 1.360)	1.055 (0.872, 1.276)	1.042 (0.861, 1.261)
Q4	**1.298 (1.081, 1.559)**	1.167 (0.971, 1.402)	1.144 (0.952, 1.376)
ITGAM	1 (Reference)	1 (Reference)	1 (Reference)
Q2	**0.805 (0.679, 0.954)**	**0.827 (0.697, 0.980)**	0.857 (0.723, 1.016)
Q3	**0.726 (0.610, 0.865)**	**0.765 (0.642, 0.911)**	**0.810 (0.680, 0.966)**
Q4	**0.638 (0.533, 0.764)**	**0.678 (0.565, 0.814)**	**0.748 (0.623, 0.899)**
ITGAV	1 (Reference)	1 (Reference)	1 (Reference)
Q2	**0.734 (0.618, 0.873)**	**0.801 (0.674, 0.953)**	0.846 (0.711, 1.007)
Q3	**0.659 (0.553, 0.784)**	**0.766 (0.642, 0.914)**	0.842 (0.704, 1.006)
Q4	**0.501 (0.417, 0.602)**	**0.638 (0.528, 0.771)**	**0.745 (0.614, 0.904)**
ITGAX	1 (Reference)	1 (Reference)	1 (Reference)
Q2	1.069 (0.893, 1.281)	1.053 (0.879, 1.261)	1.051 (0.878, 1.259)
Q3	1.048 (0.875, 1.256)	1.034 (0.863, 1.239)	1.030 (0.860, 1.234)
Q4	1.062 (0.887, 1.271)	1.046 (0.873, 1.252)	1.038 (0.867, 1.243)
ITGB1	1 (Reference)	1 (Reference)	1 (Reference)
Q2	0.916 (0.773, 1.086)	0.956 (0.807, 1.134)	0.989 (0.834, 1.172)
Q3	**0.685 (0.571, 0.821)**	**0.745 (0.622, 0.893)**	**0.802 (0.668, 0.963)**
Q4	**0.619 (0.516, 0.744)**	**0.720 (0.599, 0.867)**	**0.811 (0.672, 0.979)**
ITGB1BP1	1 (Reference)	1 (Reference)	1 (Reference)
Q2	1.076 (0.895, 1.293)	1.083 (0.901, 1.302)	1.079 (0.897, 1.297)
Q3	1.115 (0.929, 1.339)	1.121 (0.934, 1.346)	1.111 (0.926, 1.335)

Q4	**1.204 (1.006, 1.442)**	1.196 (0.999, 1.432)	1.177 (0.983, 1.409)
ITGB1BP2	1 (Reference)	1 (Reference)	1 (Reference)
Q2	1.022 (0.851, 1.228)	1.007 (0.839, 1.210)	1.003 (0.835, 1.205)
Q3	1.192 (0.998, 1.423)	1.168 (0.978, 1.395)	1.165 (0.975, 1.391)
Q4	1.120 (0.934, 1.344)	1.079 (0.900, 1.295)	1.059 (0.882, 1.271)
ITGB2	1 (Reference)	1 (Reference)	1 (Reference)
Q2	0.847 (0.712, 1.009)	0.856 (0.719, 1.019)	0.873 (0.733, 1.039)
Q3	**0.719 (0.599, 0.862)**	**0.735 (0.613, 0.883)**	**0.761 (0.634, 0.913)**
Q4	**0.795 (0.667, 0.947)**	**0.796 (0.668, 0.950)**	0.843 (0.707, 1.007)
ITGB5	1 (Reference)	1 (Reference)	1 (Reference)
Q2	1.010 (0.844, 1.208)	1.083 (0.905, 1.295)	1.127 (0.942, 1.349)
Q3	**0.797 (0.661, 0.960)**	0.879 (0.729, 1.061)	0.945 (0.783, 1.141)
Q4	0.868 (0.723, 1.042)	0.972 (0.809, 1.167)	1.060 (0.881, 1.275)
ITGB6	1 (Reference)	1 (Reference)	1 (Reference)
Q2	0.954 (0.790, 1.151)	0.974 (0.807, 1.175)	0.963 (0.798, 1.162)
Q3	1.129 (0.940, 1.355)	1.145 (0.954, 1.375)	1.113 (0.927, 1.337)
Q4	**1.262 (1.057, 1.507)**	**1.266 (1.060, 1.512)**	**1.207 (1.010, 1.443)**
ITGB7	1 (Reference)	1 (Reference)	1 (Reference)
Q2	0.871 (0.724, 1.047)	0.884 (0.735, 1.063)	0.895 (0.745, 1.077)
Q3	0.968 (0.808, 1.160)	0.969 (0.809, 1.161)	0.983 (0.820, 1.178)
Q4	1.110 (0.932, 1.321)	1.105 (0.928, 1.315)	1.113 (0.934, 1.325)
ITGBL1	1 (Reference)	1 (Reference)	1 (Reference)
Q2	0.919 (0.752, 1.124)	0.914 (0.748, 1.119)	0.909 (0.743, 1.113)
Q3	1.057 (0.871, 1.282)	1.028 (0.847, 1.248)	1.017 (0.838, 1.236)
Q4	**1.272 (1.055, 1.533)**	1.103 (0.912, 1.333)	1.065 (0.880, 1.289)

Q1 (lowest, < 25th percentile), Q2 (25th to < 50th percentile), Q3 (50th to < 75th percentile), and Q4 (highest, ⩾ 75th percentile); CI: confidence interval; ITG, Integrin; †The analysis excluded participants who developed IS within the first 2 years of follow-up.
