# Supplementary material for: The Role of Integrin Family in Atherosclerotic Cardiovascular Disease: A Prospective Cohort Study and Mendelian Randomization Analysis
Source: Biomedicines. 2026 Aug 15;14(8):1836. doi: 10.3390/biomedicines14081836 (PMC13509564; doi:10.3390/biomedicines14081836)
Supplement: Supplementary file 1 [file biomedicines-14-01836-s001.zip › TableS18.pdf]

Table S18. HR (95% CI) of incident PAD by integrin levels[†].

	Hazard Ratio (95% CI)		
Integrin	Model 1	Model 2	Model 3
ITGA11	1 (Reference)	1 (Reference)	1 (Reference)
Q2	**0.652 (0.525, 0.809)**	**0.748 (0.601, 0.929)**	**0.772 (0.621, 0.961)**
Q3	**0.547 (0.435, 0.688)**	**0.683 (0.541, 0.861)**	**0.727 (0.575, 0.919)**
Q4	**0.400 (0.312, 0.513)**	**0.553 (0.428, 0.714)**	**0.608 (0.469, 0.790)**
ITGA2	1 (Reference)	1 (Reference)	1 (Reference)
Q2	**0.716 (0.572, 0.895)**	0.801 (0.640, 1.002)	0.823 (0.657, 1.030)
Q3	**0.602 (0.477, 0.759)**	**0.703 (0.556, 0.889)**	**0.735 (0.581, 0.932)**
Q4	**0.541 (0.428, 0.685)**	**0.674 (0.531, 0.856)**	**0.723 (0.566, 0.923)**
ITGA5	1 (Reference)	1 (Reference)	1 (Reference)
Q2	1.013 (0.761, 1.350)	0.927 (0.696, 1.235)	0.920 (0.690, 1.226)
Q3	**1.448 (1.112, 1.885)**	1.268 (0.973, 1.653)	1.255 (0.962, 1.637)
Q4	**1.804 (1.401, 2.323)**	**1.343 (1.037, 1.740)**	**1.332 (1.026, 1.730)**
ITGA6	1 (Reference)	1 (Reference)	1 (Reference)
Q2	0.829 (0.659, 1.043)	0.839 (0.667, 1.055)	0.838 (0.666, 1.055)
Q3	0.914 (0.728, 1.147)	0.946 (0.754, 1.188)	0.933 (0.743, 1.172)
Q4	**0.751 (0.590, 0.957)**	**0.775 (0.609, 0.988)**	**0.766 (0.600, 0.976)**
ITGAL	1 (Reference)	1 (Reference)	1 (Reference)
Q2	1.190 (0.928, 1.525)	1.151 (0.897, 1.475)	1.148 (0.895, 1.472)
Q3	1.046 (0.813, 1.346)	0.957 (0.744, 1.232)	0.952 (0.739, 1.225)
Q4	1.186 (0.931, 1.510)	1.029 (0.808, 1.312)	1.017 (0.797, 1.297)
ITGAM	1 (Reference)	1 (Reference)	1 (Reference)
Q2	**0.620 (0.496, 0.775)**	**0.650 (0.520, 0.812)**	**0.667 (0.533, 0.833)**
Q3	**0.575 (0.458, 0.722)**	**0.624 (0.496, 0.784)**	**0.649 (0.516, 0.817)**
Q4	**0.485 (0.381, 0.615)**	**0.530 (0.416, 0.675)**	**0.567 (0.444, 0.723)**
ITGAV	1 (Reference)	1 (Reference)	1 (Reference)
Q2	**0.708 (0.567, 0.883)**	0.804 (0.643, 1.005)	0.837 (0.669, 1.047)
Q3	**0.544 (0.432, 0.685)**	**0.671 (0.531, 0.848)**	**0.716 (0.565, 0.907)**
Q4	**0.383 (0.299, 0.490)**	**0.529 (0.410, 0.682)**	**0.586 (0.452, 0.761)**
ITGAX	1 (Reference)	1 (Reference)	1 (Reference)
Q2	1.008 (0.785, 1.295)	0.973 (0.757, 1.250)	0.971 (0.756, 1.248)
Q3	1.130 (0.886, 1.440)	1.094 (0.858, 1.394)	1.087 (0.853, 1.386)
Q4	**1.304 (1.030, 1.649)**	1.257 (0.993, 1.591)	1.251 (0.989, 1.583)
ITGB1	1 (Reference)	1 (Reference)	1 (Reference)
Q2	**0.713 (0.565, 0.900)**	**0.767 (0.608, 0.969)**	**0.788 (0.623, 0.995)**
Q3	**0.684 (0.542, 0.863)**	**0.777 (0.615, 0.980)**	0.822 (0.650, 1.040)
Q4	**0.596 (0.470, 0.754)**	**0.736 (0.580, 0.934)**	0.806 (0.632, 1.028)
ITGB1BP1	1 (Reference)	1 (Reference)	1 (Reference)
Q2	1.068 (0.840, 1.357)	1.081 (0.851, 1.374)	1.075 (0.846, 1.367)
Q3	1.021 (0.801, 1.301)	1.032 (0.809, 1.315)	1.023 (0.803, 1.304)

Q4	1.122 (0.885, 1.423)	1.117 (0.881, 1.417)	1.100 (0.867, 1.396)
ITGB1BP2	1 (Reference)	1 (Reference)	1 (Reference)
Q2	1.064 (0.838, 1.351)	1.042 (0.820, 1.323)	1.041 (0.819, 1.322)
Q3	0.994 (0.778, 1.269)	0.964 (0.755, 1.231)	0.966 (0.756, 1.233)
Q4	1.244 (0.984, 1.573)	1.179 (0.932, 1.491)	1.166 (0.921, 1.477)
ITGB2	1 (Reference)	1 (Reference)	1 (Reference)
Q2	**0.762 (0.605, 0.960)**	**0.780 (0.619, 0.982)**	**0.792 (0.628, 0.997)**
Q3	**0.678 (0.536, 0.857)**	**0.710 (0.561, 0.897)**	**0.728 (0.575, 0.921)**
Q4	**0.658 (0.520, 0.831)**	**0.667 (0.527, 0.844)**	**0.695 (0.549, 0.880)**
ITGB5	1 (Reference)	1 (Reference)	1 (Reference)
Q2	0.852 (0.673, 1.078)	0.945 (0.746, 1.197)	0.973 (0.768, 1.233)
Q3	**0.708 (0.556, 0.901)**	0.804 (0.631, 1.025)	0.850 (0.666, 1.084)
Q4	**0.719 (0.567, 0.913)**	0.831 (0.654, 1.057)	0.885 (0.695, 1.128)
ITGB6	1 (Reference)	1 (Reference)	1 (Reference)
Q2	0.931 (0.726, 1.194)	0.971 (0.757, 1.245)	0.961 (0.749, 1.233)
Q3	**1.288 (1.020, 1.627)**	**1.336 (1.058, 1.688)**	**1.307 (1.034, 1.651)**
Q4	1.053 (0.826, 1.343)	1.085 (0.851, 1.384)	1.044 (0.818, 1.333)
ITGB7	1 (Reference)	1 (Reference)	1 (Reference)
Q2	0.964 (0.749, 1.241)	0.994 (0.772, 1.279)	1.005 (0.781, 1.294)
Q3	1.156 (0.905, 1.475)	1.158 (0.907, 1.479)	1.174 (0.920, 1.499)
Q4	**1.308 (1.033, 1.656)**	**1.294 (1.021, 1.639)**	**1.302 (1.027, 1.649)**
ITGBL1	1 (Reference)	1 (Reference)	1 (Reference)
Q2	1.010 (0.760, 1.342)	1.013 (0.762, 1.347)	1.010 (0.759, 1.343)
Q3	1.048 (0.794, 1.383)	1.012 (0.767, 1.336)	1.006 (0.762, 1.329)
Q4	**1.669 (1.293, 2.155)**	**1.389 (1.072, 1.801)**	**1.358 (1.046, 1.763)**

Q1 (lowest, < 25th percentile), Q2 (25th to < 50th percentile), Q3 (50th to < 75th percentile), and Q4 (highest, ⩾ 75th percentile); CI: confidence interval; ITG, Integrin. †The analysis excluded participants who developed PAD within the first 2 years of follow-up.
