# Supplementary material for: The Role of Integrin Family in Atherosclerotic Cardiovascular Disease: A Prospective Cohort Study and Mendelian Randomization Analysis
Source: Biomedicines. 2026 Aug 15;14(8):1836. doi: 10.3390/biomedicines14081836 (PMC13509564; doi:10.3390/biomedicines14081836)
Supplement: Supplementary file 1 [file biomedicines-14-01836-s001.zip › TableS19.pdf]

Table S19. HR (95% CI) of incident ASCVD by integrin levels[†].

Integrin	Hazard Ratio (95% CI)		
	Model 1	Model 2	Model 3
ITGA11	1 (Reference)	1 (Reference)	1 (Reference)
Q2	**0.743 (0.649, 0.851)**	**0.809 (0.705, 0.927)**	**0.836 (0.729, 0.958)**
Q3	**0.628 (0.545, 0.723)**	**0.727 (0.630, 0.839)**	**0.780 (0.675, 0.901)**
Q4	**0.497 (0.429, 0.577)**	**0.625 (0.537, 0.728)**	**0.697 (0.596, 0.814)**
ITGA2	1 (Reference)	1 (Reference)	1 (Reference)
Q2	**0.783 (0.680, 0.900)**	**0.849 (0.737, 0.977)**	0.882 (0.766, 1.016)
Q3	**0.720 (0.625, 0.829)**	**0.810 (0.702, 0.934)**	0.867 (0.750, 1.002)
Q4	**0.620 (0.537, 0.717)**	**0.735 (0.634, 0.851)**	**0.809 (0.696, 0.941)**
ITGA5	1 (Reference)	1 (Reference)	1 (Reference)
Q2	1.140 (0.968, 1.341)	1.064 (0.904, 1.253)	1.056 (0.897, 1.243)
Q3	**1.269 (1.084, 1.485)**	1.121 (0.957, 1.314)	1.111 (0.948, 1.302)
Q4	**1.447 (1.241, 1.686)**	1.141 (0.975, 1.334)	1.128 (0.963, 1.321)
ITGA6	1 (Reference)	1 (Reference)	1 (Reference)
Q2	1.105 (0.959, 1.274)	1.121 (0.973, 1.292)	1.115 (0.967, 1.285)
Q3	1.021 (0.883, 1.181)	1.036 (0.895, 1.198)	1.023 (0.885, 1.184)
Q4	0.918 (0.790, 1.068)	0.932 (0.802, 1.084)	0.929 (0.798, 1.080)
ITGAL	1 (Reference)	1 (Reference)	1 (Reference)
Q2	1.147 (0.986, 1.333)	1.121 (0.964, 1.303)	1.114 (0.958, 1.295)
Q3	1.019 (0.874, 1.187)	0.961 (0.824, 1.120)	0.955 (0.819, 1.113)
Q4	**1.159 (1.001, 1.342)**	1.039 (0.897, 1.204)	1.024 (0.884, 1.187)
ITGAM	1 (Reference)	1 (Reference)	1 (Reference)
Q2	**0.784 (0.681, 0.902)**	**0.803 (0.698, 0.924)**	**0.828 (0.719, 0.952)**
Q3	**0.756 (0.657, 0.870)**	**0.795 (0.691, 0.915)**	**0.832 (0.722, 0.958)**
Q4	**0.628 (0.542, 0.728)**	**0.666 (0.573, 0.772)**	**0.721 (0.620, 0.838)**
ITGAV	1 (Reference)	1 (Reference)	1 (Reference)
Q2	**0.725 (0.629, 0.836)**	**0.789 (0.684, 0.910)**	**0.825 (0.715, 0.952)**
Q3	**0.637 (0.553, 0.735)**	**0.739 (0.640, 0.853)**	**0.797 (0.689, 0.923)**
Q4	**0.465 (0.400, 0.540)**	**0.590 (0.506, 0.689)**	**0.667 (0.569, 0.782)**
ITGAX	1 (Reference)	1 (Reference)	1 (Reference)
Q2	1.041 (0.895, 1.209)	1.041 (0.896, 1.210)	1.046 (0.900, 1.216)
Q3	1.090 (0.939, 1.264)	1.080 (0.931, 1.253)	1.075 (0.927, 1.247)
Q4	**1.202 (1.040, 1.389)**	**1.188 (1.028, 1.374)**	**1.179 (1.020, 1.363)**
ITGB1	1 (Reference)	1 (Reference)	1 (Reference)
Q2	**0.865 (0.750, 0.998)**	0.908 (0.787, 1.048)	0.936 (0.810, 1.080)
Q3	**0.718 (0.620, 0.831)**	**0.788 (0.680, 0.913)**	**0.844 (0.727, 0.979)**
Q4	**0.664 (0.573, 0.769)**	**0.780 (0.672, 0.905)**	0.869 (0.746, 1.011)
ITGB1BP1	1 (Reference)	1 (Reference)	1 (Reference)
Q2	1.043 (0.900, 1.208)	1.048 (0.905, 1.214)	1.047 (0.903, 1.213)
Q3	1.106 (0.956, 1.279)	1.114 (0.964, 1.289)	1.116 (0.965, 1.291)

Q4	1.022 (0.881, 1.185)	1.018 (0.878, 1.181)	1.009 (0.870, 1.171)
ITGB1BP2	1 (Reference)	1 (Reference)	1 (Reference)
Q2	0.910 (0.785, 1.055)	0.892 (0.769, 1.034)	0.894 (0.771, 1.036)
Q3	1.026 (0.888, 1.185)	0.997 (0.863, 1.152)	1.002 (0.867, 1.157)
Q4	1.085 (0.939, 1.253)	1.046 (0.906, 1.209)	1.043 (0.902, 1.206)
ITGB2	1 (Reference)	1 (Reference)	1 (Reference)
Q2	**0.776 (0.673, 0.896)**	**0.779 (0.675, 0.898)**	**0.789 (0.684, 0.911)**
Q3	**0.673 (0.582, 0.780)**	**0.685 (0.592, 0.794)**	**0.706 (0.609, 0.817)**
Q4	**0.741 (0.643, 0.854)**	**0.736 (0.638, 0.848)**	**0.773 (0.670, 0.891)**
ITGB5	1 (Reference)	1 (Reference)	1 (Reference)
Q2	0.863 (0.743, 1.002)	0.924 (0.795, 1.074)	0.957 (0.824, 1.113)
Q3	**0.821 (0.707, 0.952)**	0.911 (0.785, 1.059)	0.975 (0.838, 1.133)
Q4	**0.830 (0.716, 0.961)**	0.937 (0.808, 1.087)	1.020 (0.878, 1.185)
ITGB6	1 (Reference)	1 (Reference)	1 (Reference)
Q2	0.954 (0.819, 1.111)	0.971 (0.834, 1.130)	0.960 (0.825, 1.118)
Q3	**1.164 (1.007, 1.347)**	**1.183 (1.023, 1.369)**	**1.159 (1.002, 1.340)**
Q4	1.137 (0.983, 1.315)	1.129 (0.976, 1.307)	1.091 (0.942, 1.263)
ITGB7	1 (Reference)	1 (Reference)	1 (Reference)
Q2	0.866 (0.745, 1.007)	0.878 (0.756, 1.021)	0.893 (0.768, 1.038)
Q3	0.976 (0.844, 1.130)	0.990 (0.855, 1.146)	1.009 (0.872, 1.169)
Q4	1.060 (0.919, 1.222)	1.070 (0.928, 1.234)	1.079 (0.935, 1.244)
ITGBL1	1 (Reference)	1 (Reference)	1 (Reference)
Q2	0.907 (0.768, 1.071)	0.902 (0.764, 1.066)	0.900 (0.762, 1.063)
Q3	0.993 (0.846, 1.166)	0.972 (0.827, 1.142)	0.962 (0.819, 1.131)
Q4	**1.294 (1.112, 1.506)**	1.134 (0.972, 1.323)	1.105 (0.947, 1.291)

Q1 (lowest, < 25th percentile), Q2 (25th to < 50th percentile), Q3 (50th to < 75th percentile), and Q4 (highest, ⩾ 75th percentile); CI: confidence interval; ITG, Integrin. †Analyses are based on complete cases, without imputation for missing covariates.
