# Supplementary material for: The Role of Integrin Family in Atherosclerotic Cardiovascular Disease: A Prospective Cohort Study and Mendelian Randomization Analysis
Source: Biomedicines. 2026 Aug 15;14(8):1836. doi: 10.3390/biomedicines14081836 (PMC13509564; doi:10.3390/biomedicines14081836)
Supplement: Supplementary file 1 [file biomedicines-14-01836-s001.zip › TableS2.pdf]

**Table S2. HR (95% CI) of incident ASCVD by integrin levels.**

| Integrin | Hazard Ratio (95% CI) | | |
| --- | --- | --- | --- |
| | Model 1 | Model 2 | Model 3 |
| The overall population | | | |
| ITGA11 | 1 (Reference) | 1 (Reference) | 1 (Reference) |
| Q2 | **0.744 (0.671, 0.824)** | **0.819 (0.738, 0.908)** | **0.842 (0.759, 0.935)** |
| Q3 | **0.625 (0.561, 0.697)** | **0.739 (0.662, 0.825)** | **0.786 (0.703, 0.878)** |
| Q4 | **0.485 (0.432, 0.544)** | **0.617 (0.549, 0.695)** | **0.678 (0.601, 0.765)** |
| ITGA2 | 1 (Reference) | 1 (Reference) | 1 (Reference) |
| Q2 | **0.771 (0.693, 0.856)** | **0.833 (0.749, 0.926)** | **0.855 (0.769, 0.951)** |
| Q3 | **0.664 (0.596, 0.740)** | **0.748 (0.671, 0.834)** | **0.785 (0.703, 0.877)** |
| Q4 | **0.551 (0.492, 0.616)** | **0.656 (0.586, 0.736)** | **0.704 (0.627, 0.791)** |
| ITGA5 | 1 (Reference) | 1 (Reference) | 1 (Reference) |
| Q2 | **1.193 (1.051, 1.353)** | 1.110 (0.978, 1.260) | 1.106 (0.974, 1.255) |
| Q3 | **1.307 (1.156, 1.478)** | **1.167 (1.032, 1.320)** | **1.160 (1.025, 1.313)** |
| Q4 | **1.541 (1.369, 1.735)** | **1.214 (1.076, 1.371)** | **1.208 (1.069, 1.365)** |
| ITGA6 | 1 (Reference) | 1 (Reference) | 1 (Reference) |
| Q2 | 1.007 (0.904, 1.123) | 1.020 (0.915, 1.136) | 1.016 (0.911, 1.132) |
| Q3 | 0.987 (0.884, 1.102) | 1.009 (0.903, 1.127) | 0.997 (0.893, 1.114) |
| Q4 | **0.888 (0.792, 0.995)** | 0.909 (0.811, 1.019) | 0.901 (0.803, 1.010) |
| ITGAL | 1 (Reference) | 1 (Reference) | 1 (Reference) |
| Q2 | 1.085 (0.966, 1.218) | 1.057 (0.942, 1.187) | 1.054 (0.939, 1.184) |
| Q3 | 1.007 (0.896, 1.131) | 0.943 (0.839, 1.059) | 0.936 (0.833, 1.052) |
| Q4 | 1.110 (0.992, 1.242) | 0.993 (0.887, 1.112) | 0.981 (0.876, 1.099) |
| ITGAM | 1 (Reference) | 1 (Reference) | 1 (Reference) |
| Q2 | **0.813 (0.731, 0.905)** | **0.838 (0.753, 0.932)** | **0.860 (0.773, 0.956)** |
| Q3 | **0.743 (0.667, 0.829)** | **0.783 (0.702, 0.873)** | **0.814 (0.730, 0.908)** |
| Q4 | **0.630 (0.562, 0.705)** | **0.666 (0.594, 0.747)** | **0.714 (0.636, 0.801)** |
| ITGAV | 1 (Reference) | 1 (Reference) | 1 (Reference) |
| Q2 | **0.772 (0.694, 0.859)** | **0.850 (0.764, 0.946)** | **0.883 (0.793, 0.983)** |
| Q3 | **0.596 (0.534, 0.665)** | **0.701 (0.627, 0.784)** | **0.748 (0.668, 0.838)** |
| Q4 | **0.470 (0.419, 0.527)** | **0.608 (0.540, 0.685)** | **0.676 (0.599, 0.763)** |
| ITGAX | 1 (Reference) | 1 (Reference) | 1 (Reference) |
| Q2 | 1.053 (0.940, 1.180) | 1.033 (0.923, 1.158) | 1.035 (0.924, 1.160) |
| Q3 | 1.030 (0.920, 1.154) | 1.015 (0.906, 1.137) | 1.012 (0.903, 1.134) |
| Q4 | **1.133 (1.014, 1.266)** | 1.112 (0.995, 1.243) | 1.107 (0.990, 1.237) |
| ITGB1 | 1 (Reference) | 1 (Reference) | 1 (Reference) |
| Q2 | **0.814 (0.731, 0.907)** | **0.856 (0.768, 0.953)** | **0.877 (0.787, 0.978)** |
| Q3 | **0.663 (0.593, 0.742)** | **0.729 (0.651, 0.815)** | **0.770 (0.688, 0.862)** |
| Q4 | **0.620 (0.554, 0.693)** | **0.730 (0.652, 0.817)** | **0.797 (0.711, 0.895)** |
| ITGB1BP1 | 1 (Reference) | 1 (Reference) | 1 (Reference) |
| Q2 | 1.025 (0.916, 1.148) | 1.035 (0.925, 1.159) | 1.032 (0.922, 1.155) |
| Q3 | 1.059 (0.946, 1.184) | 1.069 (0.955, 1.196) | 1.063 (0.950, 1.189) |

| | | | |
|---|---|---|---|
| Q4 | 1.057 (0.944, 1.182) | 1.050 (0.938, 1.174) | 1.038 (0.928, 1.162) |
| ITGB1BP2 | 1 (Reference) | 1 (Reference) | 1 (Reference) |
| Q2 | 1.003 (0.896, 1.122) | 0.989 (0.884, 1.106) | 0.988 (0.883, 1.105) |
| Q3 | 1.042 (0.932, 1.165) | 1.017 (0.910, 1.137) | 1.019 (0.912, 1.140) |
| Q4 | 1.081 (0.967, 1.209) | 1.040 (0.930, 1.163) | 1.031 (0.922, 1.154) |
| ITGB2 | 1 (Reference) | 1 (Reference) | 1 (Reference) |
| Q2 | **0.814 (0.730, 0.907)** | **0.823 (0.739, 0.918)** | **0.835 (0.749, 0.931)** |
| Q3 | **0.695 (0.621, 0.778)** | **0.712 (0.636, 0.798)** | **0.729 (0.651, 0.817)** |
| Q4 | **0.727 (0.651, 0.811)** | **0.725 (0.650, 0.810)** | **0.756 (0.676, 0.844)** |
| ITGB5 | 1 (Reference) | 1 (Reference) | 1 (Reference) |
| Q2 | **0.847 (0.756, 0.949)** | 0.911 (0.813, 1.021) | 0.940 (0.839, 1.053) |
| Q3 | **0.802 (0.716, 0.898)** | **0.892 (0.796, 1.000)** | 0.943 (0.841, 1.057) |
| Q4 | **0.789 (0.705, 0.884)** | **0.892 (0.796, 0.999)** | 0.954 (0.851, 1.070) |
| ITGB6 | 1 (Reference) | 1 (Reference) | 1 (Reference) |
| Q2 | 0.948 (0.844, 1.064) | 0.970 (0.863, 1.089) | 0.963 (0.857, 1.081) |
| Q3 | **1.172 (1.048, 1.310)** | **1.197 (1.070, 1.338)** | **1.176 (1.051, 1.315)** |
| Q4 | **1.126 (1.007, 1.260)** | **1.134 (1.013, 1.268)** | 1.098 (0.981, 1.229) |
| ITGB7 | 1 (Reference) | 1 (Reference) | 1 (Reference) |
| Q2 | **0.847 (0.755, 0.949)** | **0.862 (0.769, 0.966)** | **0.871 (0.777, 0.977)** |
| Q3 | 0.956 (0.855, 1.069) | 0.957 (0.855, 1.070) | 0.969 (0.867, 1.084) |
| Q4 | 1.032 (0.925, 1.151) | 1.027 (0.920, 1.145) | 1.035 (0.928, 1.154) |
| ITGBL1 | 1 (Reference) | 1 (Reference) | 1 (Reference) |
| Q2 | 0.987 (0.868, 1.121) | 0.991 (0.872, 1.126) | 0.988 (0.869, 1.123) |
| Q3 | 1.087 (0.960, 1.230) | 1.065 (0.940, 1.205) | 1.057 (0.934, 1.197) |
| Q4 | **1.350 (1.198, 1.520)** | **1.174 (1.041, 1.325)** | **1.149 (1.018, 1.297)** |

Q1 (lowest, < 25th percentile), Q2 (25th to < 50th percentile), Q3 (50th to < 75th percentile), and Q4 (highest, ⩾ 75th percentile); CI: confidence interval; ITG, Integrin.
