# Supplementary material for: The Role of Integrin Family in Atherosclerotic Cardiovascular Disease: A Prospective Cohort Study and Mendelian Randomization Analysis
Source: Biomedicines. 2026 Aug 15;14(8):1836. doi: 10.3390/biomedicines14081836 (PMC13509564; doi:10.3390/biomedicines14081836)
Supplement: Supplementary file 1 [file biomedicines-14-01836-s001.zip › TableS20.pdf]

Table S20. HR (95% CI) of incident CHD by integrin levels[†].

	Hazard Ratio (95% CI)		
Integrin	Model 1	Model 2	Model 3
ITGA11	1 (Reference)	1 (Reference)	1 (Reference)
Q2	**0.717 (0.603, 0.851)**	**0.789 (0.664, 0.938)**	**0.827 (0.696, 0.984)**
Q3	**0.605 (0.506, 0.724)**	**0.721 (0.601, 0.864)**	**0.795 (0.662, 0.955)**
Q4	**0.503 (0.418, 0.606)**	**0.656 (0.542, 0.794)**	**0.765 (0.629, 0.930)**
ITGA2	1 (Reference)	1 (Reference)	1 (Reference)
Q2	**0.732 (0.612, 0.876)**	**0.801 (0.669, 0.959)**	0.844 (0.705, 1.011)
Q3	**0.733 (0.614, 0.875)**	0.840 (0.703, 1.004)	0.924 (0.771, 1.106)
Q4	**0.610 (0.508, 0.732)**	**0.743 (0.617, 0.894)**	0.850 (0.702, 1.029)
ITGA5	1 (Reference)	1 (Reference)	1 (Reference)
Q2	1.229 (0.997, 1.517)	1.132 (0.918, 1.397)	1.120 (0.907, 1.382)
Q3	**1.350 (1.101, 1.656)**	1.166 (0.950, 1.431)	1.149 (0.935, 1.411)
Q4	**1.631 (1.340, 1.985)**	**1.230 (1.006, 1.504)**	1.208 (0.986, 1.480)
ITGA6	1 (Reference)	1 (Reference)	1 (Reference)
Q2	1.119 (0.937, 1.338)	1.138 (0.952, 1.360)	1.129 (0.944, 1.349)
Q3	1.005 (0.836, 1.208)	1.021 (0.850, 1.228)	1.006 (0.837, 1.210)
Q4	0.875 (0.722, 1.060)	0.890 (0.734, 1.078)	0.884 (0.729, 1.073)
ITGAL	1 (Reference)	1 (Reference)	1 (Reference)
Q2	**1.296 (1.066, 1.576)**	**1.260 (1.036, 1.533)**	**1.247 (1.025, 1.516)**
Q3	1.174 (0.963, 1.430)	1.094 (0.898, 1.334)	1.083 (0.888, 1.319)
Q4	**1.348 (1.115, 1.630)**	1.185 (0.979, 1.434)	1.162 (0.959, 1.406)
ITGAM	1 (Reference)	1 (Reference)	1 (Reference)
Q2	**0.800 (0.671, 0.953)**	**0.820 (0.688, 0.977)**	0.855 (0.717, 1.020)
Q3	**0.732 (0.613, 0.876)**	**0.774 (0.647, 0.926)**	**0.825 (0.689, 0.988)**
Q4	**0.616 (0.511, 0.743)**	**0.651 (0.539, 0.787)**	**0.727 (0.601, 0.880)**
ITGAV	1 (Reference)	1 (Reference)	1 (Reference)
Q2	**0.713 (0.597, 0.852)**	**0.789 (0.660, 0.944)**	0.842 (0.704, 1.008)
Q3	**0.603 (0.503, 0.722)**	**0.719 (0.599, 0.864)**	**0.801 (0.665, 0.965)**
Q4	**0.464 (0.384, 0.560)**	**0.617 (0.508, 0.749)**	**0.735 (0.602, 0.897)**
ITGAX	1 (Reference)	1 (Reference)	1 (Reference)
Q2	1.118 (0.926, 1.349)	1.119 (0.927, 1.351)	1.124 (0.931, 1.356)
Q3	1.086 (0.899, 1.312)	1.076 (0.891, 1.300)	1.070 (0.886, 1.293)
Q4	1.174 (0.975, 1.413)	1.159 (0.963, 1.396)	1.145 (0.951, 1.379)
ITGB1	1 (Reference)	1 (Reference)	1 (Reference)
Q2	0.932 (0.780, 1.115)	0.984 (0.823, 1.177)	1.023 (0.855, 1.224)
Q3	**0.748 (0.622, 0.901)**	0.832 (0.691, 1.002)	0.911 (0.755, 1.099)
Q4	**0.632 (0.523, 0.765)**	**0.755 (0.624, 0.915)**	0.871 (0.716, 1.059)
ITGB1BP1	1 (Reference)	1 (Reference)	1 (Reference)
Q2	1.067 (0.886, 1.285)	1.079 (0.896, 1.299)	1.077 (0.894, 1.297)
Q3	1.063 (0.882, 1.281)	1.070 (0.888, 1.290)	1.073 (0.890, 1.293)

Q4	1.085 (0.900, 1.307)	1.085 (0.900, 1.307)	1.070 (0.888, 1.290)
ITGB1BP2	1 (Reference)	1 (Reference)	1 (Reference)
Q2	0.873 (0.723, 1.054)	0.854 (0.707, 1.031)	0.856 (0.709, 1.034)
Q3	1.078 (0.901, 1.290)	1.045 (0.873, 1.250)	1.049 (0.876, 1.255)
Q4	1.029 (0.856, 1.236)	0.988 (0.822, 1.187)	0.980 (0.814, 1.179)
ITGB2	1 (Reference)	1 (Reference)	1 (Reference)
Q2	**0.831 (0.693, 0.996)**	**0.829 (0.692, 0.994)**	0.846 (0.705, 1.014)
Q3	**0.718 (0.596, 0.864)**	**0.726 (0.603, 0.875)**	**0.757 (0.628, 0.912)**
Q4	**0.775 (0.647, 0.929)**	**0.758 (0.632, 0.909)**	**0.811 (0.676, 0.973)**
ITGB5	1 (Reference)	1 (Reference)	1 (Reference)
Q2	0.955 (0.792, 1.150)	1.031 (0.855, 1.242)	1.079 (0.895, 1.301)
Q3	**0.785 (0.648, 0.951)**	0.888 (0.732, 1.077)	0.970 (0.799, 1.178)
Q4	0.848 (0.703, 1.022)	0.974 (0.807, 1.176)	1.083 (0.895, 1.310)
ITGB6	1 (Reference)	1 (Reference)	1 (Reference)
Q2	0.908 (0.744, 1.108)	0.928 (0.760, 1.132)	0.912 (0.747, 1.113)
Q3	**1.308 (1.089, 1.571)**	**1.333 (1.110, 1.602)**	**1.293 (1.076, 1.553)**
Q4	**1.226 (1.018, 1.476)**	**1.214 (1.009, 1.462)**	1.154 (0.958, 1.390)
ITGB7	1 (Reference)	1 (Reference)	1 (Reference)
Q2	**0.812 (0.674, 0.979)**	**0.826 (0.685, 0.995)**	0.844 (0.700, 1.018)
Q3	0.843 (0.701, 1.014)	0.854 (0.710, 1.028)	0.876 (0.728, 1.054)
Q4	0.967 (0.810, 1.155)	0.973 (0.814, 1.162)	0.981 (0.821, 1.172)
ITGBL1	1 (Reference)	1 (Reference)	1 (Reference)
Q2	0.831 (0.674, 1.023)	0.822 (0.667, 1.013)	0.818 (0.664, 1.009)
Q3	0.938 (0.768, 1.145)	0.911 (0.745, 1.113)	0.898 (0.735, 1.098)
Q4	**1.222 (1.012, 1.476)**	1.037 (0.856, 1.257)	0.999 (0.823, 1.212)

Q1 (lowest, < 25th percentile), Q2 (25th to < 50th percentile), Q3 (50th to < 75th percentile), and Q4 (highest, ⩾ 75th percentile); CI: confidence interval; ITG, Integrin. †Analyses are based on complete cases, without imputation for missing covariates.
