# Supplementary material for: The Role of Integrin Family in Atherosclerotic Cardiovascular Disease: A Prospective Cohort Study and Mendelian Randomization Analysis
Source: Biomedicines. 2026 Aug 15;14(8):1836. doi: 10.3390/biomedicines14081836 (PMC13509564; doi:10.3390/biomedicines14081836)
Supplement: Supplementary file 1 [file biomedicines-14-01836-s001.zip › TableS21.pdf]

Table S21. HR (95% CI) of incident IS by integrin levels†.

	Hazard Ratio (95% CI)		
Integrin	Model 1	Model 2	Model 3
ITGA11	1 (Reference)	1 (Reference)	1 (Reference)
Q2	**0.678 (0.551, 0.835)**	**0.733 (0.595, 0.903)**	**0.768 (0.623, 0.947)**
Q3	**0.610 (0.493, 0.755)**	**0.701 (0.565, 0.870)**	**0.771 (0.620, 0.959)**
Q4	**0.499 (0.400, 0.624)**	**0.617 (0.491, 0.776)**	**0.717 (0.568, 0.907)**
ITGA2	1 (Reference)	1 (Reference)	1 (Reference)
Q2	**0.747 (0.602, 0.928)**	**0.804 (0.647, 0.999)**	0.844 (0.679, 1.050)
Q3	**0.788 (0.638, 0.973)**	0.880 (0.711, 1.088)	0.958 (0.772, 1.188)
Q4	**0.646 (0.518, 0.805)**	**0.758 (0.606, 0.947)**	0.853 (0.678, 1.073)
ITGA5	1 (Reference)	1 (Reference)	1 (Reference)
Q2	1.140 (0.883, 1.470)	1.062 (0.823, 1.371)	1.045 (0.810, 1.349)
Q3	**1.444 (1.135, 1.838)**	1.272 (0.998, 1.620)	1.243 (0.975, 1.585)
Q4	**1.649 (1.304, 2.086)**	**1.300 (1.022, 1.654)**	1.258 (0.987, 1.604)
ITGA6	1 (Reference)	1 (Reference)	1 (Reference)
Q2	1.215 (0.980, 1.507)	1.230 (0.992, 1.525)	1.217 (0.982, 1.509)
Q3	1.015 (0.810, 1.271)	1.024 (0.817, 1.282)	1.006 (0.803, 1.261)
Q4	0.968 (0.770, 1.218)	0.977 (0.776, 1.229)	0.963 (0.764, 1.213)
ITGAL	1 (Reference)	1 (Reference)	1 (Reference)
Q2	**1.374 (1.080, 1.747)**	**1.338 (1.052, 1.702)**	**1.323 (1.040, 1.682)**
Q3	**1.285 (1.010, 1.636)**	1.209 (0.950, 1.540)	1.191 (0.935, 1.517)
Q4	**1.547 (1.228, 1.950)**	**1.388 (1.100, 1.751)**	**1.353 (1.073, 1.708)**
ITGAM	1 (Reference)	1 (Reference)	1 (Reference)
Q2	0.828 (0.670, 1.024)	0.846 (0.684, 1.046)	0.882 (0.713, 1.092)
Q3	0.810 (0.655, 1.002)	0.849 (0.687, 1.051)	0.907 (0.732, 1.124)
Q4	**0.633 (0.505, 0.795)**	**0.665 (0.529, 0.836)**	**0.742 (0.588, 0.935)**
ITGAV	1 (Reference)	1 (Reference)	1 (Reference)
Q2	**0.705 (0.567, 0.877)**	**0.764 (0.614, 0.951)**	0.814 (0.653, 1.014)
Q3	**0.683 (0.551, 0.847)**	**0.787 (0.633, 0.978)**	0.874 (0.701, 1.090)
Q4	**0.524 (0.419, 0.657)**	**0.661 (0.524, 0.834)**	**0.785 (0.618, 0.996)**
ITGAX	1 (Reference)	1 (Reference)	1 (Reference)
Q2	0.990 (0.792, 1.236)	0.990 (0.792, 1.237)	0.991 (0.793, 1.238)
Q3	0.980 (0.784, 1.225)	0.973 (0.778, 1.216)	0.966 (0.773, 1.207)
Q4	1.059 (0.851, 1.318)	1.049 (0.843, 1.306)	1.035 (0.832, 1.288)
ITGB1	1 (Reference)	1 (Reference)	1 (Reference)
Q2	0.950 (0.767, 1.178)	0.988 (0.797, 1.225)	1.023 (0.825, 1.269)
Q3	0.802 (0.643, 1.000)	0.872 (0.699, 1.087)	0.946 (0.757, 1.182)
Q4	**0.638 (0.506, 0.804)**	**0.734 (0.582, 0.927)**	0.835 (0.658, 1.059)
ITGB1BP1	1 (Reference)	1 (Reference)	1 (Reference)
Q2	1.093 (0.872, 1.370)	1.099 (0.877, 1.378)	1.095 (0.874, 1.373)
Q3	1.138 (0.910, 1.425)	1.140 (0.911, 1.427)	1.138 (0.909, 1.424)

Q4	1.146 (0.916, 1.435)	1.144 (0.914, 1.432)	1.123 (0.897, 1.406)
ITGB1BP2	1 (Reference)	1 (Reference)	1 (Reference)
Q2	0.857 (0.680, 1.079)	0.840 (0.667, 1.058)	0.840 (0.667, 1.058)
Q3	1.193 (0.964, 1.476)	1.162 (0.939, 1.438)	1.159 (0.936, 1.435)
Q4	1.053 (0.843, 1.314)	1.014 (0.812, 1.266)	0.995 (0.796, 1.244)
ITGB2	1 (Reference)	1 (Reference)	1 (Reference)
Q2	0.867 (0.697, 1.077)	0.867 (0.697, 1.078)	0.884 (0.711, 1.099)
Q3	**0.737 (0.589, 0.923)**	**0.745 (0.595, 0.933)**	**0.776 (0.620, 0.972)**
Q4	0.822 (0.662, 1.021)	0.810 (0.651, 1.007)	0.865 (0.695, 1.076)
ITGB5	1 (Reference)	1 (Reference)	1 (Reference)
Q2	1.055 (0.843, 1.320)	1.122 (0.896, 1.405)	1.171 (0.935, 1.468)
Q3	0.853 (0.675, 1.077)	0.939 (0.743, 1.187)	1.023 (0.808, 1.294)
Q4	0.942 (0.751, 1.182)	1.050 (0.836, 1.319)	1.156 (0.918, 1.456)
ITGB6	1 (Reference)	1 (Reference)	1 (Reference)
Q2	0.946 (0.747, 1.197)	0.960 (0.759, 1.215)	0.945 (0.747, 1.196)
Q3	1.194 (0.955, 1.493)	1.206 (0.964, 1.508)	1.169 (0.934, 1.462)
Q4	**1.266 (1.016, 1.577)**	**1.250 (1.003, 1.558)**	1.185 (0.950, 1.478)
ITGB7	1 (Reference)	1 (Reference)	1 (Reference)
Q2	0.861 (0.688, 1.078)	0.873 (0.697, 1.092)	0.887 (0.709, 1.111)
Q3	0.880 (0.704, 1.100)	0.891 (0.713, 1.114)	0.909 (0.727, 1.136)
Q4	1.024 (0.826, 1.268)	1.031 (0.832, 1.278)	1.036 (0.836, 1.285)
ITGBL1	1 (Reference)	1 (Reference)	1 (Reference)
Q2	0.932 (0.729, 1.191)	0.924 (0.723, 1.181)	0.918 (0.718, 1.174)
Q3	0.996 (0.784, 1.265)	0.974 (0.766, 1.237)	0.957 (0.752, 1.217)
Q4	1.234 (0.982, 1.552)	1.078 (0.854, 1.360)	1.032 (0.816, 1.305)

Q1 (lowest, < 25th percentile), Q2 (25th to < 50th percentile), Q3 (50th to < 75th percentile), and Q4 (highest, ⩾ 75th percentile); CI: confidence interval; ITG, Integrin. †Analyses are based on complete cases, without imputation for missing covariates.
