# Supplementary material for: The Role of Integrin Family in Atherosclerotic Cardiovascular Disease: A Prospective Cohort Study and Mendelian Randomization Analysis
Source: Biomedicines. 2026 Aug 15;14(8):1836. doi: 10.3390/biomedicines14081836 (PMC13509564; doi:10.3390/biomedicines14081836)
Supplement: Supplementary file 1 [file biomedicines-14-01836-s001.zip › TableS22.pdf]

Table S22. HR (95% CI) of incident PAD by integrin levels†.

Integrin	Hazard Ratio (95% CI)		
	Model 1	Model 2	Model 3
ITGA11	1 (Reference)	1 (Reference)	1 (Reference)
Q2	**0.591 (0.442, 0.789)**	**0.660 (0.494, 0.883)**	**0.682 (0.509, 0.913)**
Q3	**0.569 (0.426, 0.762)**	**0.679 (0.506, 0.913)**	**0.724 (0.538, 0.976)**
Q4	**0.371 (0.267, 0.514)**	**0.487 (0.348, 0.682)**	**0.540 (0.383, 0.760)**
ITGA2	1 (Reference)	1 (Reference)	1 (Reference)
Q2	**0.713 (0.530, 0.958)**	0.795 (0.590, 1.069)	0.828 (0.615, 1.116)
Q3	**0.669 (0.497, 0.900)**	0.769 (0.570, 1.037)	0.825 (0.609, 1.118)
Q4	**0.523 (0.383, 0.714)**	**0.631 (0.460, 0.867)**	**0.699 (0.505, 0.969)**
ITGA5	1 (Reference)	1 (Reference)	1 (Reference)
Q2	0.960 (0.671, 1.374)	0.889 (0.621, 1.274)	0.885 (0.618, 1.268)
Q3	1.133 (0.806, 1.594)	0.990 (0.703, 1.395)	0.987 (0.700, 1.391)
Q4	**1.611 (1.172, 2.213)**	1.224 (0.884, 1.695)	1.228 (0.884, 1.705)
ITGA6	1 (Reference)	1 (Reference)	1 (Reference)
Q2	0.900 (0.661, 1.225)	0.922 (0.677, 1.256)	0.921 (0.677, 1.254)
Q3	1.016 (0.752, 1.373)	1.045 (0.773, 1.411)	1.037 (0.767, 1.401)
Q4	0.898 (0.656, 1.229)	0.922 (0.674, 1.263)	0.925 (0.675, 1.269)
ITGAL	1 (Reference)	1 (Reference)	1 (Reference)
Q2	0.979 (0.713, 1.344)	0.953 (0.694, 1.309)	0.948 (0.690, 1.301)
Q3	0.865 (0.627, 1.193)	0.805 (0.583, 1.111)	0.805 (0.583, 1.110)
Q4	1.023 (0.755, 1.386)	0.890 (0.656, 1.208)	0.882 (0.650, 1.198)
ITGAM	1 (Reference)	1 (Reference)	1 (Reference)
Q2	**0.624 (0.465, 0.838)**	**0.645 (0.480, 0.866)**	**0.664 (0.494, 0.893)**
Q3	**0.628 (0.469, 0.840)**	**0.664 (0.495, 0.889)**	**0.693 (0.517, 0.930)**
Q4	**0.472 (0.343, 0.649)**	**0.504 (0.366, 0.696)**	**0.544 (0.393, 0.753)**
ITGAV	1 (Reference)	1 (Reference)	1 (Reference)
Q2	**0.711 (0.531, 0.953)**	0.784 (0.584, 1.053)	0.821 (0.611, 1.105)
Q3	**0.543 (0.401, 0.736)**	**0.642 (0.472, 0.874)**	**0.691 (0.506, 0.945)**
Q4	**0.384 (0.279, 0.530)**	**0.503 (0.360, 0.701)**	**0.567 (0.402, 0.798)**
ITGAX	1 (Reference)	1 (Reference)	1 (Reference)
Q2	0.974 (0.698, 1.360)	0.963 (0.689, 1.344)	0.968 (0.693, 1.352)
Q3	1.207 (0.879, 1.656)	1.181 (0.861, 1.621)	1.175 (0.856, 1.612)
Q4	1.354 (0.995, 1.842)	1.314 (0.965, 1.788)	1.307 (0.960, 1.779)
ITGB1	1 (Reference)	1 (Reference)	1 (Reference)
Q2	**0.713 (0.522, 0.974)**	0.761 (0.557, 1.039)	0.787 (0.576, 1.076)
Q3	**0.707 (0.521, 0.960)**	0.794 (0.584, 1.078)	0.858 (0.630, 1.169)
Q4	**0.633 (0.465, 0.861)**	0.763 (0.560, 1.041)	0.861 (0.627, 1.183)
ITGB1BP1	1 (Reference)	1 (Reference)	1 (Reference)
Q2	1.060 (0.777, 1.447)	1.071 (0.785, 1.462)	1.070 (0.784, 1.461)
Q3	1.056 (0.773, 1.443)	1.072 (0.785, 1.465)	1.077 (0.788, 1.471)

Q4	1.026 (0.748, 1.407)	1.033 (0.753, 1.417)	1.027 (0.749, 1.410)
ITGB1BP2	1 (Reference)	1 (Reference)	1 (Reference)
Q2	0.964 (0.702, 1.324)	0.932 (0.679, 1.280)	0.941 (0.685, 1.293)
Q3	0.943 (0.684, 1.299)	0.906 (0.658, 1.249)	0.916 (0.665, 1.263)
Q4	1.316 (0.975, 1.778)	1.258 (0.932, 1.699)	1.272 (0.940, 1.721)
ITGB2	1 (Reference)	1 (Reference)	1 (Reference)
Q2	**0.690 (0.510, 0.934)**	**0.700 (0.518, 0.948)**	**0.713 (0.527, 0.966)**
Q3	**0.624 (0.459, 0.847)**	**0.639 (0.470, 0.868)**	**0.659 (0.485, 0.897)**
Q4	**0.631 (0.466, 0.853)**	**0.628 (0.463, 0.850)**	**0.662 (0.488, 0.897)**
ITGB5	1 (Reference)	1 (Reference)	1 (Reference)
Q2	0.878 (0.640, 1.205)	0.965 (0.703, 1.325)	1.000 (0.728, 1.373)
Q3	0.729 (0.528, 1.007)	0.816 (0.589, 1.130)	0.877 (0.632, 1.217)
Q4	0.828 (0.607, 1.129)	0.947 (0.693, 1.295)	1.040 (0.758, 1.427)
ITGB6	1 (Reference)	1 (Reference)	1 (Reference)
Q2	0.799 (0.578, 1.105)	0.818 (0.592, 1.131)	0.807 (0.584, 1.116)
Q3	1.160 (0.863, 1.560)	1.185 (0.882, 1.594)	1.157 (0.860, 1.556)
Q4	0.882 (0.644, 1.208)	0.883 (0.645, 1.210)	0.852 (0.621, 1.168)
ITGB7	1 (Reference)	1 (Reference)	1 (Reference)
Q2	0.987 (0.706, 1.380)	1.009 (0.722, 1.411)	1.030 (0.736, 1.440)
Q3	1.151 (0.833, 1.591)	1.169 (0.845, 1.616)	1.200 (0.867, 1.659)
Q4	**1.387 (1.018, 1.889)**	**1.387 (1.018, 1.890)**	**1.405 (1.031, 1.915)**
ITGBL1	1 (Reference)	1 (Reference)	1 (Reference)
Q2	0.814 (0.562, 1.178)	0.810 (0.559, 1.173)	0.811 (0.560, 1.176)
Q3	0.816 (0.568, 1.171)	0.794 (0.552, 1.141)	0.792 (0.551, 1.139)
Q4	**1.561 (1.135, 2.147)**	1.336 (0.967, 1.847)	1.316 (0.950, 1.823)

Q1 (lowest, < 25th percentile), Q2 (25th to < 50th percentile), Q3 (50th to < 75th percentile), and Q4 (highest, ⩾ 75th percentile); CI: confidence interval; ITG, Integrin. †Analyses are based on complete cases, without imputation for missing covariates.
