# Supplementary material for: The Role of Integrin Family in Atherosclerotic Cardiovascular Disease: A Prospective Cohort Study and Mendelian Randomization Analysis
Source: Biomedicines. 2026 Aug 15;14(8):1836. doi: 10.3390/biomedicines14081836 (PMC13509564; doi:10.3390/biomedicines14081836)
Supplement: Supplementary file 1 [file biomedicines-14-01836-s001.zip › TableS3.pdf]

Table S3. HR (95% CI) of incident CHD by integrin levels.

	Hazard Ratio (95% CI)		
Integrin	Model 1	Model 2	Model 3
The overall population			
ITGA11	1 (Reference)	1 (Reference)	1 (Reference)
Q2	**0.703 (0.617, 0.800)**	**0.786 (0.689, 0.896)**	**0.820 (0.719, 0.935)**
Q3	**0.593 (0.517, 0.680)**	**0.719 (0.625, 0.826)**	**0.787 (0.684, 0.905)**
Q4	**0.467 (0.404, 0.540)**	**0.617 (0.531, 0.716)**	**0.708 (0.608, 0.824)**
ITGA2	1 (Reference)	1 (Reference)	1 (Reference)
Q2	**0.724 (0.634, 0.826)**	**0.790 (0.691, 0.902)**	**0.823 (0.720, 0.940)**
Q3	**0.639 (0.558, 0.732)**	**0.732 (0.638, 0.839)**	**0.789 (0.687, 0.905)**
Q4	**0.498 (0.432, 0.575)**	**0.610 (0.527, 0.706)**	**0.681 (0.587, 0.791)**
ITGA5	1 (Reference)	1 (Reference)	1 (Reference)
Q2	**1.238 (1.055, 1.452)**	1.134 (0.967, 1.331)	1.128 (0.961, 1.323)
Q3	**1.316 (1.126, 1.539)**	1.149 (0.982, 1.344)	1.138 (0.972, 1.333)
Q4	**1.581 (1.360, 1.838)**	**1.192 (1.022, 1.391)**	**1.184 (1.014, 1.384)**
ITGA6	1 (Reference)	1 (Reference)	1 (Reference)
Q2	0.997 (0.869, 1.142)	1.008 (0.879, 1.155)	1.003 (0.875, 1.150)
Q3	0.966 (0.840, 1.111)	0.989 (0.860, 1.137)	0.975 (0.848, 1.122)
Q4	**0.858 (0.742, 0.991)**	0.879 (0.760, 1.016)	0.872 (0.754, 1.008)
ITGAL	1 (Reference)	1 (Reference)	1 (Reference)
Q2	1.158 (1.000, 1.341)	1.123 (0.969, 1.300)	1.118 (0.965, 1.295)
Q3	1.027 (0.885, 1.193)	0.954 (0.822, 1.108)	0.946 (0.814, 1.098)
Q4	**1.177 (1.020, 1.358)**	1.036 (0.898, 1.196)	1.022 (0.885, 1.180)
ITGAM	1 (Reference)	1 (Reference)	1 (Reference)
Q2	**0.808 (0.707, 0.923)**	**0.833 (0.729, 0.952)**	**0.864 (0.756, 0.988)**
Q3	**0.717 (0.625, 0.822)**	**0.760 (0.662, 0.872)**	**0.803 (0.699, 0.922)**
Q4	**0.597 (0.517, 0.690)**	**0.636 (0.550, 0.736)**	**0.702 (0.606, 0.812)**
ITGAV	1 (Reference)	1 (Reference)	1 (Reference)
Q2	**0.765 (0.670, 0.874)**	**0.852 (0.746, 0.974)**	0.902 (0.789, 1.031)
Q3	**0.558 (0.485, 0.643)**	**0.671 (0.582, 0.774)**	**0.740 (0.641, 0.854)**
Q4	**0.452 (0.391, 0.522)**	**0.607 (0.523, 0.705)**	**0.711 (0.610, 0.829)**
ITGAX	1 (Reference)	1 (Reference)	1 (Reference)
Q2	1.082 (0.938, 1.247)	1.062 (0.921, 1.225)	1.063 (0.921, 1.226)
Q3	1.024 (0.886, 1.182)	1.008 (0.872, 1.164)	1.003 (0.869, 1.159)
Q4	1.102 (0.956, 1.269)	1.079 (0.937, 1.244)	1.072 (0.931, 1.235)
ITGB1	1 (Reference)	1 (Reference)	1 (Reference)
Q2	**0.855 (0.748, 0.977)**	0.904 (0.791, 1.033)	0.937 (0.819, 1.071)
Q3	**0.638 (0.553, 0.735)**	**0.708 (0.614, 0.817)**	**0.767 (0.664, 0.885)**
Q4	**0.568 (0.492, 0.656)**	**0.682 (0.590, 0.788)**	**0.774 (0.668, 0.898)**
ITGB1BP1	1 (Reference)	1 (Reference)	1 (Reference)
Q2	1.060 (0.919, 1.222)	1.072 (0.930, 1.237)	1.068 (0.926, 1.232)
Q3	1.060 (0.919, 1.223)	1.072 (0.929, 1.237)	1.064 (0.923, 1.228)

Q4	1.072 (0.930, 1.236)	1.066 (0.924, 1.229)	1.051 (0.911, 1.212)
ITGB1BP2	1 (Reference)	1 (Reference)	1 (Reference)
Q2	0.959 (0.833, 1.104)	0.942 (0.818, 1.085)	0.941 (0.817, 1.084)
Q3	1.021 (0.888, 1.173)	0.995 (0.865, 1.143)	0.997 (0.867, 1.147)
Q4	1.002 (0.870, 1.154)	0.961 (0.834, 1.108)	0.952 (0.825, 1.097)
ITGB2	1 (Reference)	1 (Reference)	1 (Reference)
Q2	0.883 (0.770, 1.012)	0.891 (0.777, 1.022)	0.911 (0.794, 1.044)
Q3	**0.731 (0.633, 0.844)**	**0.749 (0.649, 0.865)**	**0.776 (0.672, 0.896)**
Q4	**0.751 (0.653, 0.864)**	**0.747 (0.648, 0.860)**	**0.792 (0.687, 0.912)**
ITGB5	1 (Reference)	1 (Reference)	1 (Reference)
Q2	0.890 (0.773, 1.024)	0.966 (0.839, 1.111)	1.008 (0.875, 1.160)
Q3	**0.753 (0.652, 0.869)**	**0.849 (0.735, 0.981)**	0.916 (0.793, 1.059)
Q4	**0.737 (0.639, 0.851)**	**0.846 (0.733, 0.978)**	0.929 (0.804, 1.075)
ITGB6	1 (Reference)	1 (Reference)	1 (Reference)
Q2	0.914 (0.787, 1.063)	0.939 (0.808, 1.092)	0.930 (0.800, 1.080)
Q3	**1.259 (1.093, 1.450)**	**1.288 (1.119, 1.484)**	**1.255 (1.090, 1.446)**
Q4	**1.202 (1.043, 1.386)**	**1.210 (1.050, 1.395)**	**1.156 (1.002, 1.333)**
ITGB7	1 (Reference)	1 (Reference)	1 (Reference)
Q2	**0.813 (0.704, 0.937)**	**0.828 (0.718, 0.955)**	**0.842 (0.730, 0.971)**
Q3	**0.866 (0.751, 0.997)**	**0.867 (0.753, 0.999)**	0.883 (0.766, 1.017)
Q4	0.981 (0.856, 1.124)	0.974 (0.849, 1.116)	0.982 (0.857, 1.126)
ITGBL1	1 (Reference)	1 (Reference)	1 (Reference)
Q2	0.928 (0.791, 1.090)	0.925 (0.788, 1.086)	0.922 (0.785, 1.083)
Q3	1.029 (0.882, 1.201)	0.994 (0.851, 1.160)	0.986 (0.845, 1.152)
Q4	**1.265 (1.091, 1.467)**	1.065 (0.916, 1.238)	1.033 (0.888, 1.202)

Q1 (lowest, < 25th percentile), Q2 (25th to < 50th percentile), Q3 (50th to < 75th percentile), and Q4 (highest, ⩾ 75th percentile); CI: confidence interval; ITG, Integrin.
