# Supplementary material for: The Role of Integrin Family in Atherosclerotic Cardiovascular Disease: A Prospective Cohort Study and Mendelian Randomization Analysis
Source: Biomedicines. 2026 Aug 15;14(8):1836. doi: 10.3390/biomedicines14081836 (PMC13509564; doi:10.3390/biomedicines14081836)
Supplement: Supplementary file 1 [file biomedicines-14-01836-s001.zip › TableS4.pdf]

Table S4. HR (95% CI) of incident IS by integrin levels.

	Hazard Ratio (95% CI)		
Integrin	Model 1	Model 2	Model 3
The overall population			
ITGA11	1 (Reference)	1 (Reference)	1 (Reference)
Q2	**0.706 (0.603, 0.827)**	**0.775 (0.661, 0.908)**	**0.808 (0.689, 0.946)**
Q3	**0.591 (0.501, 0.698)**	**0.693 (0.585, 0.820)**	**0.756 (0.638, 0.897)**
Q4	**0.474 (0.398, 0.565)**	**0.597 (0.498, 0.716)**	**0.685 (0.570, 0.823)**
ITGA2	1 (Reference)	1 (Reference)	1 (Reference)
Q2	**0.729 (0.620, 0.858)**	**0.785 (0.667, 0.924)**	**0.816 (0.693, 0.961)**
Q3	**0.683 (0.580, 0.805)**	**0.767 (0.650, 0.904)**	**0.820 (0.694, 0.970)**
Q4	**0.545 (0.459, 0.647)**	**0.647 (0.543, 0.770)**	**0.712 (0.595, 0.852)**
ITGA5	1 (Reference)	1 (Reference)	1 (Reference)
Q2	1.212 (0.997, 1.475)	1.126 (0.925, 1.371)	1.114 (0.915, 1.356)
Q3	**1.476 (1.223, 1.780)**	**1.311 (1.085, 1.583)**	**1.289 (1.066, 1.557)**
Q4	**1.626 (1.353, 1.955)**	**1.277 (1.058, 1.542)**	**1.250 (1.034, 1.512)**
ITGA6	1 (Reference)	1 (Reference)	1 (Reference)
Q2	1.112 (0.941, 1.313)	1.120 (0.948, 1.323)	1.113 (0.942, 1.314)
Q3	1.007 (0.847, 1.197)	1.024 (0.861, 1.217)	1.006 (0.846, 1.196)
Q4	0.963 (0.808, 1.148)	0.980 (0.822, 1.168)	0.964 (0.808, 1.150)
ITGAL	1 (Reference)	1 (Reference)	1 (Reference)
Q2	**1.244 (1.038, 1.491)**	**1.212 (1.011, 1.452)**	**1.205 (1.006, 1.445)**
Q3	1.146 (0.955, 1.376)	1.075 (0.895, 1.290)	1.061 (0.884, 1.274)
Q4	**1.313 (1.101, 1.566)**	1.178 (0.987, 1.406)	1.155 (0.967, 1.379)
ITGAM	1 (Reference)	1 (Reference)	1 (Reference)
Q2	**0.824 (0.700, 0.969)**	**0.847 (0.719, 0.996)**	0.878 (0.746, 1.033)
Q3	**0.754 (0.638, 0.891)**	**0.795 (0.673, 0.940)**	**0.843 (0.713, 0.997)**
Q4	**0.640 (0.538, 0.761)**	**0.681 (0.571, 0.811)**	**0.751 (0.630, 0.897)**
ITGAV	1 (Reference)	1 (Reference)	1 (Reference)
Q2	**0.757 (0.643, 0.893)**	**0.828 (0.702, 0.977)**	0.875 (0.741, 1.033)
Q3	**0.650 (0.550, 0.769)**	**0.758 (0.640, 0.899)**	**0.834 (0.702, 0.990)**
Q4	**0.503 (0.422, 0.600)**	**0.644 (0.537, 0.772)**	**0.753 (0.625, 0.906)**
ITGAX	1 (Reference)	1 (Reference)	1 (Reference)
Q2	1.058 (0.891, 1.257)	1.043 (0.877, 1.239)	1.041 (0.876, 1.236)
Q3	1.042 (0.877, 1.238)	1.028 (0.865, 1.221)	1.023 (0.861, 1.216)
Q4	1.051 (0.885, 1.249)	1.034 (0.871, 1.229)	1.026 (0.864, 1.219)
ITGB1	1 (Reference)	1 (Reference)	1 (Reference)
Q2	0.911 (0.775, 1.072)	0.953 (0.810, 1.120)	0.985 (0.837, 1.159)
Q3	**0.663 (0.557, 0.789)**	**0.723 (0.607, 0.860)**	**0.778 (0.652, 0.927)**
Q4	**0.627 (0.526, 0.746)**	**0.731 (0.613, 0.872)**	**0.823 (0.688, 0.984)**
ITGB1BP1	1 (Reference)	1 (Reference)	1 (Reference)
Q2	1.090 (0.915, 1.300)	1.098 (0.921, 1.309)	1.094 (0.918, 1.304)
Q3	1.148 (0.965, 1.366)	1.154 (0.970, 1.373)	1.144 (0.962, 1.362)

Q4	1.164 (0.979, 1.384)	1.156 (0.972, 1.375)	1.137 (0.955, 1.352)
ITGB1BP2	1 (Reference)	1 (Reference)	1 (Reference)
Q2	0.973 (0.818, 1.157)	0.958 (0.806, 1.140)	0.953 (0.801, 1.134)
Q3	1.105 (0.933, 1.308)	1.083 (0.915, 1.282)	1.078 (0.911, 1.277)
Q4	1.053 (0.886, 1.251)	1.014 (0.854, 1.205)	0.994 (0.836, 1.182)
ITGB2	1 (Reference)	1 (Reference)	1 (Reference)
Q2	0.871 (0.738, 1.028)	0.880 (0.745, 1.038)	0.897 (0.760, 1.059)
Q3	**0.711 (0.597, 0.847)**	**0.727 (0.610, 0.866)**	**0.752 (0.631, 0.896)**
Q4	**0.780 (0.659, 0.923)**	**0.781 (0.659, 0.925)**	**0.827 (0.698, 0.980)**
ITGB5	1 (Reference)	1 (Reference)	1 (Reference)
Q2	0.989 (0.834, 1.173)	1.060 (0.894, 1.258)	1.103 (0.930, 1.309)
Q3	**0.793 (0.664, 0.947)**	0.875 (0.733, 1.046)	0.941 (0.787, 1.125)
Q4	**0.834 (0.700, 0.993)**	0.935 (0.784, 1.114)	1.018 (0.853, 1.216)
ITGB6	1 (Reference)	1 (Reference)	1 (Reference)
Q2	0.949 (0.793, 1.137)	0.969 (0.809, 1.161)	0.958 (0.800, 1.147)
Q3	1.140 (0.958, 1.357)	1.156 (0.971, 1.376)	1.123 (0.943, 1.337)
Q4	**1.268 (1.070, 1.502)**	**1.271 (1.072, 1.506)**	**1.210 (1.020, 1.434)**
ITGB7	1 (Reference)	1 (Reference)	1 (Reference)
Q2	0.862 (0.724, 1.028)	0.876 (0.735, 1.044)	0.888 (0.745, 1.058)
Q3	0.949 (0.798, 1.127)	0.951 (0.800, 1.130)	0.964 (0.811, 1.145)
Q4	1.075 (0.910, 1.270)	1.071 (0.906, 1.265)	1.078 (0.912, 1.273)
ITGBL1	1 (Reference)	1 (Reference)	1 (Reference)
Q2	0.940 (0.775, 1.141)	0.935 (0.770, 1.135)	0.929 (0.765, 1.128)
Q3	1.109 (0.922, 1.335)	1.077 (0.895, 1.296)	**1.065 (0.885, 1.283)**
Q4	**1.280 (1.069, 1.534)**	1.106 (0.921, 1.328)	**1.065 (0.886, 1.281)**

Q1 (lowest, < 25th percentile), Q2 (25th to < 50th percentile), Q3 (50th to < 75th percentile), and Q4 (highest, ⩾ 75th percentile); CI: confidence interval; ITG, Integrin.
