# Supplementary material for: The Role of Integrin Family in Atherosclerotic Cardiovascular Disease: A Prospective Cohort Study and Mendelian Randomization Analysis
Source: Biomedicines. 2026 Aug 15;14(8):1836. doi: 10.3390/biomedicines14081836 (PMC13509564; doi:10.3390/biomedicines14081836)
Supplement: Supplementary file 1 [file biomedicines-14-01836-s001.zip › TableS5.pdf]

Table S5. HR (95% CI) of incident PAD by integrin levels.

Integrin	Hazard Ratio (95% CI)		
	Model 1	Model 2	Model 3
The overall population			
ITGA11	1 (Reference)	1 (Reference)	1 (Reference)
Q2	**0.608 (0.493, 0.750)**	**0.697 (0.564, 0.860)**	**0.717 (0.581, 0.886)**
Q3	**0.536 (0.431, 0.666)**	**0.667 (0.534, 0.833)**	**0.706 (0.564, 0.884)**
Q4	**0.384 (0.302, 0.487)**	**0.527 (0.412, 0.675)**	**0.576 (0.448, 0.740)**
ITGA2	1 (Reference)	1 (Reference)	1 (Reference)
Q2	**0.758 (0.612, 0.938)**	0.846 (0.683, 1.048)	0.869 (0.701, 1.078)
Q3	**0.618 (0.495, 0.772)**	**0.723 (0.577, 0.905)**	**0.757 (0.603, 0.949)**
Q4	**0.526 (0.418, 0.661)**	**0.655 (0.519, 0.827)**	**0.704 (0.555, 0.894)**
ITGA5	1 (Reference)	1 (Reference)	1 (Reference)
Q2	1.053 (0.799, 1.389)	0.965 (0.732, 1.273)	0.961 (0.729, 1.268)
Q3	**1.453 (1.124, 1.878)**	1.273 (0.983, 1.647)	1.266 (0.977, 1.639)
Q4	**1.888 (1.478, 2.412)**	**1.412 (1.099, 1.814)**	**1.414 (1.099, 1.820)**
ITGA6	1 (Reference)	1 (Reference)	1 (Reference)
Q2	0.852 (0.683, 1.063)	0.865 (0.693, 1.080)	0.865 (0.693, 1.080)
Q3	0.952 (0.765, 1.185)	0.988 (0.794, 1.229)	0.977 (0.785, 1.217)
Q4	**0.765 (0.606, 0.967)**	0.794 (0.628, 1.003)	**0.788 (0.623, 0.997)**
ITGAL	1 (Reference)	1 (Reference)	1 (Reference)
Q2	1.097 (0.863, 1.394)	1.060 (0.834, 1.347)	1.058 (0.833, 1.345)
Q3	1.044 (0.822, 1.326)	0.956 (0.753, 1.214)	0.953 (0.750, 1.211)
Q4	1.146 (0.910, 1.443)	0.994 (0.788, 1.253)	0.986 (0.782, 1.244)
ITGAM	1 (Reference)	1 (Reference)	1 (Reference)
Q2	**0.632 (0.511, 0.782)**	**0.661 (0.534, 0.818)**	**0.677 (0.547, 0.838)**
Q3	**0.584 (0.470, 0.727)**	**0.632 (0.507, 0.787)**	**0.656 (0.526, 0.818)**
Q4	**0.471 (0.373, 0.594)**	**0.511 (0.404, 0.647)**	**0.545 (0.430, 0.690)**
ITGAV	1 (Reference)	1 (Reference)	1 (Reference)
Q2	**0.688 (0.556, 0.853)**	**0.782 (0.630, 0.970)**	0.813 (0.655, 1.009)
Q3	**0.538 (0.431, 0.672)**	**0.664 (0.530, 0.832)**	**0.707 (0.563, 0.888)**
Q4	**0.391 (0.309, 0.495)**	**0.541 (0.424, 0.691)**	**0.598 (0.466, 0.768)**
ITGAX	1 (Reference)	1 (Reference)	1 (Reference)
Q2	1.055 (0.828, 1.343)	1.018 (0.799, 1.296)	1.017 (0.798, 1.295)
Q3	1.162 (0.918, 1.470)	1.125 (0.889, 1.424)	1.119 (0.884, 1.416)
Q4	**1.359 (1.082, 1.706)**	**1.310 (1.043, 1.645)**	**1.304 (1.038, 1.638)**
ITGB1	1 (Reference)	1 (Reference)	1 (Reference)
Q2	**0.743 (0.594, 0.930)**	**0.799 (0.638, 0.999)**	0.821 (0.656, 1.028)
Q3	**0.698 (0.557, 0.874)**	**0.792 (0.632, 0.992)**	0.839 (0.669, 1.054)
Q4	**0.619 (0.493, 0.777)**	**0.764 (0.607, 0.961)**	0.838 (0.663, 1.058)
ITGB1BP1	1(Reference)	1 (Reference)	1 (Reference)
Q2	1.039 (0.825, 1.307)	1.053 (0.836, 1.325)	1.048 (0.832, 1.318)
Q3	0.989 (0.784, 1.249)	1.000 (0.792, 1.263)	0.994 (0.787, 1.254)

Q4	1.102 (0.878, 1.383)	1.097 (0.874, 1.377)	1.084 (0.863, 1.361)
ITGB1BP2	1 (Reference)	1 (Reference)	1 (Reference)
Q2	1.071 (0.849, 1.351)	1.049 (0.831, 1.323)	1.050 (0.832, 1.325)
Q3	1.042 (0.824, 1.319)	1.012 (0.800, 1.281)	1.017 (0.804, 1.287)
Q4	**1.296 (1.034, 1.625)**	1.229 (0.980, 1.541)	1.225 (0.975, 1.538)
ITGB2	1 (Reference)	1 (Reference)	1 (Reference)
Q2	**0.724 (0.580, 0.904)**	**0.739 (0.592, 0.923)**	**0.751 (0.602, 0.938)**
Q3	**0.633 (0.504, 0.794)**	**0.661 (0.527, 0.829)**	**0.677 (0.540, 0.850)**
Q4	**0.652 (0.522, 0.814)**	**0.658 (0.526, 0.822)**	**0.685 (0.547, 0.857)**
ITGB5	1 (Reference)	1 (Reference)	1 (Reference)
Q2	0.891 (0.709, 1.121)	0.989 (0.786, 1.244)	1.018 (0.808, 1.281)
Q3	**0.766 (0.607, 0.967)**	0.872 (0.690, 1.102)	0.921 (0.728, 1.165)
Q4	**0.765 (0.607, 0.964)**	0.886 (0.702, 1.118)	0.946 (0.748, 1.197)
ITGB6	1 (Reference)	1 (Reference)	1 (Reference)
Q2	0.900 (0.708, 1.144)	0.937 (0.737, 1.192)	0.929 (0.730, 1.181)
Q3	**1.273 (1.018, 1.592)**	**1.319 (1.055, 1.650)**	**1.293 (1.034, 1.617)**
Q4	1.042 (0.825, 1.315)	1.071 (0.848, 1.352)	1.035 (0.819, 1.308)
ITGB7	1 (Reference)	1 (Reference)	1 (Reference)
Q2	0.933 (0.731, 1.190)	0.960 (0.752, 1.225)	0.971 (0.761, 1.239)
Q3	1.093 (0.863, 1.385)	1.095 (0.864, 1.387)	1.110 (0.876, 1.407)
Q4	**1.352 (1.081, 1.693)**	**1.333 (1.065, 1.669)**	**1.343 (1.073, 1.681)**
ITGBL1	1 (Reference)	1 (Reference)	1 (Reference)
Q2	1.050 (0.797, 1.384)	1.056 (0.801, 1.393)	1.055 (0.800, 1.391)
Q3	1.097 (0.838, 1.435)	1.063 (0.811, 1.391)	1.059 (0.809, 1.388)
Q4	**1.759 (1.372, 2.254)**	**1.470 (1.142, 1.891)**	**1.446 (1.122, 1.863)**

Q1 (lowest, < 25th percentile), Q2 (25th to < 50th percentile), Q3 (50th to < 75th percentile), and Q4 (highest, ⩾ 75th percentile); CI: confidence interval; ITG, Integrin.
