# Supplementary material for: The Role of Integrin Family in Atherosclerotic Cardiovascular Disease: A Prospective Cohort Study and Mendelian Randomization Analysis
Source: Biomedicines. 2026 Aug 15;14(8):1836. doi: 10.3390/biomedicines14081836 (PMC13509564; doi:10.3390/biomedicines14081836)
Supplement: Supplementary file 1 [file biomedicines-14-01836-s001.zip › TableS6.pdf]

Table S6. Sex-stratified HR (95% CI) of incident ASCVD by integrin levels.

Integrin	Hazard Ratio (95% CI)		
	Model 1	Model 2	Model 3
Male			
ITGA11	1 (Reference)	1 (Reference)	1 (Reference)
Q2	**0.729 (0.638, 0.833)**	**0.780 (0.682, 0.892)**	**0.795 (0.695, 0.910)**
Q3	**0.717 (0.626, 0.822)**	**0.800 (0.697, 0.917)**	**0.832 (0.725, 0.956)**
Q4	**0.577 (0.499, 0.667)**	**0.664 (0.572, 0.770)**	**0.702 (0.604, 0.816)**
ITGA2	1 (Reference)	1 (Reference)	1 (Reference)
Q2	1.021 (0.893, 1.166)	1.047 (0.916, 1.197)	1.058 (0.925, 1.209)
Q3	**0.811 (0.705, 0.935)**	**0.845 (0.733, 0.973)**	**0.862 (0.748, 0.993)**
Q4	**0.700 (0.605, 0.810)**	**0.740 (0.639, 0.856)**	**0.763 (0.658, 0.885)**
ITGA5	1 (Reference)	1 (Reference)	1 (Reference)
Q2	**1.170 (1.001, 1.368)**	1.104 (0.944, 1.291)	1.088 (0.930, 1.272)
Q3	**1.273 (1.093, 1.484)**	**1.168 (1.001, 1.363)**	1.149 (0.984, 1.341)
Q4	**1.705 (1.474, 1.971)**	**1.426 (1.226, 1.658)**	**1.390 (1.193, 1.619)**
ITGA6	1 (Reference)	1 (Reference)	1 (Reference)
Q2	1.071 (0.933, 1.229)	1.090 (0.950, 1.251)	1.090 (0.950, 1.252)
Q3	1.033 (0.898, 1.189)	1.070 (0.930, 1.231)	1.064 (0.925, 1.224)
Q4	0.889 (0.767, 1.030)	0.922 (0.795, 1.069)	0.916 (0.790, 1.063)
ITGAL	1 (Reference)	1 (Reference)	1 (Reference)
Q2	1.130 (0.975, 1.310)	1.104 (0.952, 1.279)	1.094 (0.944, 1.268)
Q3	1.054 (0.909, 1.223)	0.997 (0.859, 1.156)	0.979 (0.843, 1.136)
Q4	**1.255 (1.089, 1.447)**	1.126 (0.976, 1.300)	1.100 (0.953, 1.270)
ITGAM	1 (Reference)	1 (Reference)	1 (Reference)
Q2	**0.848 (0.738, 0.975)**	**0.850 (0.740, 0.978)**	**0.863 (0.751, 0.993)**
Q3	0.883 (0.769, 1.013)	0.892 (0.777, 1.024)	0.909 (0.792, 1.044)
Q4	**0.767 (0.665, 0.884)**	**0.749 (0.648, 0.865)**	**0.772 (0.668, 0.892)**
ITGAV	1 (Reference)	1 (Reference)	1 (Reference)
Q2	**0.796 (0.695, 0.913)**	**0.856 (0.746, 0.981)**	0.880 (0.767, 1.010)
Q3	**0.645 (0.560, 0.743)**	**0.713 (0.618, 0.822)**	**0.741 (0.642, 0.856)**
Q4	**0.603 (0.523, 0.694)**	**0.697 (0.604, 0.805)**	**0.743 (0.641, 0.861)**
ITGAX	1 (Reference)	1 (Reference)	1 (Reference)
Q2	1.123 (0.970, 1.300)	1.086 (0.938, 1.258)	1.084 (0.936, 1.255)
Q3	1.120 (0.967, 1.297)	1.096 (0.946, 1.270)	1.095 (0.945, 1.268)
Q4	**1.246 (1.080, 1.437)**	**1.203 (1.043, 1.388)**	**1.200 (1.040, 1.384)**
ITGB1	1 (Reference)	1 (Reference)	1 (Reference)
Q2	0.920 (0.802, 1.054)	0.939 (0.819, 1.077)	0.954 (0.832, 1.094)
Q3	**0.733 (0.635, 0.847)**	**0.760 (0.658, 0.879)**	**0.781 (0.676, 0.903)**
Q4	**0.796 (0.692, 0.916)**	**0.827 (0.719, 0.952)**	**0.863 (0.749, 0.995)**
ITGB1BP1	1 (Reference)	1 (Reference)	1 (Reference)
Q2	1.010 (0.876, 1.164)	1.036 (0.899, 1.194)	1.033 (0.896, 1.190)
Q3	0.999 (0.865, 1.153)	1.025 (0.888, 1.183)	1.021 (0.884, 1.179)

Q4	1.066 (0.925, 1.229)	1.072 (0.930, 1.235)	1.065 (0.924, 1.227)
ITGB1BP2	1 (Reference)	1 (Reference)	1 (Reference)
Q2	1.041 (0.904, 1.199)	1.039 (0.902, 1.197)	1.038 (0.901, 1.196)
Q3	1.035 (0.898, 1.193)	1.016 (0.882, 1.171)	1.019 (0.884, 1.175)
Q4	1.044 (0.904, 1.204)	1.018 (0.882, 1.175)	1.011 (0.875, 1.167)
ITGB2	1 (Reference)	1 (Reference)	1 (Reference)
Q2	0.871 (0.754, 1.006)	**0.861 (0.746, 0.995)**	**0.858 (0.743, 0.991)**
Q3	**0.846 (0.733, 0.977)**	**0.829 (0.718, 0.958)**	**0.832 (0.720, 0.961)**
Q4	0.990 (0.862, 1.136)	0.921 (0.801, 1.059)	0.918 (0.799, 1.055)
ITGB5	1 (Reference)	1 (Reference)	1 (Reference)
Q2	0.907 (0.785, 1.048)	0.946 (0.818, 1.093)	0.962 (0.832, 1.112)
Q3	0.918 (0.796, 1.058)	0.972 (0.843, 1.121)	1.006 (0.872, 1.161)
Q4	**0.835 (0.722, 0.965)**	0.875 (0.756, 1.012)	0.908 (0.784, 1.051)
ITGB6	1 (Reference)	1 (Reference)	1 (Reference)
Q2	1.053 (0.916, 1.211)	1.101 (0.958, 1.266)	1.104 (0.960, 1.269)
Q3	0.970 (0.841, 1.118)	1.031 (0.894, 1.189)	1.037 (0.899, 1.196)
Q4	0.908 (0.786, 1.048)	1.003 (0.868, 1.160)	1.009 (0.872, 1.166)
ITGB7	1 (Reference)	1 (Reference)	1 (Reference)
Q2	0.921 (0.798, 1.062)	0.921 (0.799, 1.063)	0.926 (0.803, 1.069)
Q3	0.964 (0.836, 1.112)	0.956 (0.829, 1.103)	0.959 (0.831, 1.106)
Q4	1.072 (0.933, 1.231)	1.029 (0.895, 1.183)	1.018 (0.886, 1.171)
ITGBL1	1 (Reference)	1 (Reference)	1 (Reference)
Q2	0.998 (0.846, 1.178)	1.005 (0.851, 1.186)	0.996 (0.843, 1.176)
Q3	1.132 (0.965, 1.328)	1.085 (0.924, 1.274)	1.064 (0.905, 1.250)
Q4	**1.467 (1.259, 1.710)**	**1.281 (1.094, 1.500)**	**1.240 (1.058, 1.454)**

	Hazard Ratio (95% CI)		
Integrin	Model 1	Model 2	Model 3
Female			
ITGA11	1 (Reference)	1 (Reference)	1 (Reference)
Q2	**0.692 (0.582, 0.822)**	**0.736 (0.619, 0.875)**	**0.753 (0.632, 0.896)**
Q3	**0.623 (0.523, 0.741)**	**0.692 (0.580, 0.825)**	**0.725 (0.607, 0.867)**
Q4	**0.495 (0.413, 0.594)**	**0.576 (0.477, 0.695)**	**0.615 (0.508, 0.745)**
ITGA2	1 (Reference)	1 (Reference)	1 (Reference)
Q2	**0.795 (0.666, 0.949)**	**0.831 (0.696, 0.993)**	0.844 (0.707, 1.009)
Q3	**0.683 (0.570, 0.819)**	**0.729 (0.608, 0.875)**	**0.745 (0.620, 0.895)**
Q4	**0.698 (0.585, 0.833)**	**0.756 (0.633, 0.903)**	**0.774 (0.646, 0.928)**
ITGA5	1 (Reference)	1 (Reference)	1 (Reference)
Q2	**1.352 (1.087, 1.681)**	**1.277 (1.026, 1.589)**	**1.266 (1.017, 1.576)**
Q3	**1.478 (1.195, 1.827)**	**1.350 (1.090, 1.672)**	**1.326 (1.070, 1.643)**
Q4	**1.722 (1.401, 2.116)**	**1.424 (1.150, 1.764)**	**1.369 (1.104, 1.697)**
ITGA6	1 (Reference)	1 (Reference)	1 (Reference)
Q2	0.934 (0.782, 1.116)	0.931 (0.779, 1.112)	0.919 (0.769, 1.099)

Q3	0.941 (0.787, 1.126)	0.939 (0.785, 1.123)	0.917 (0.766, 1.097)
Q4	0.936 (0.782, 1.121)	0.927 (0.774, 1.110)	0.906 (0.756, 1.086)
ITGAL	1 (Reference)	1 (Reference)	1 (Reference)
Q2	0.979 (0.804, 1.191)	0.945 (0.777, 1.150)	0.940 (0.772, 1.144)
Q3	1.149 (0.954, 1.384)	1.069 (0.886, 1.288)	1.050 (0.870, 1.266)
Q4	1.119 (0.931, 1.345)	1.003 (0.833, 1.208)	0.969 (0.804, 1.167)
ITGAM	1 (Reference)	1 (Reference)	1 (Reference)
Q2	**0.799 (0.671, 0.952)**	**0.807 (0.677, 0.962)**	**0.814 (0.683, 0.970)**
Q3	**0.750 (0.629, 0.894)**	**0.758 (0.636, 0.904)**	**0.777 (0.651, 0.926)**
Q4	**0.671 (0.560, 0.804)**	**0.680 (0.567, 0.816)**	**0.710 (0.591, 0.852)**
ITGAV	1 (Reference)	1 (Reference)	1 (Reference)
Q2	**0.815 (0.684, 0.971)**	0.863 (0.724, 1.029)	0.877 (0.735, 1.047)
Q3	**0.591 (0.492, 0.709)**	**0.660 (0.548, 0.795)**	**0.685 (0.568, 0.826)**
Q4	**0.465 (0.385, 0.562)**	**0.539 (0.444, 0.654)**	**0.573 (0.471, 0.698)**
ITGAX	1 (Reference)	1 (Reference)	1 (Reference)
Q2	0.972 (0.812, 1.163)	0.965 (0.806, 1.155)	0.965 (0.806, 1.155)
Q3	0.919 (0.766, 1.102)	0.910 (0.759, 1.091)	0.900 (0.751, 1.080)
Q4	1.014 (0.849, 1.211)	1.006 (0.842, 1.201)	0.993 (0.831, 1.186)
ITGB1	1 (Reference)	1 (Reference)	1 (Reference)
Q2	**0.739 (0.612, 0.891)**	**0.772 (0.639, 0.931)**	**0.784 (0.650, 0.947)**
Q3	**0.712 (0.592, 0.856)**	**0.755 (0.627, 0.909)**	**0.784 (0.651, 0.945)**
Q4	**0.686 (0.570, 0.824)**	**0.753 (0.626, 0.907)**	**0.795 (0.659, 0.960)**
ITGB1BP1	1 (Reference)	1 (Reference)	1 (Reference)
Q2	1.066 (0.886, 1.282)	1.060 (0.881, 1.275)	1.056 (0.877, 1.270)
Q3	1.193 (0.997, 1.428)	1.180 (0.986, 1.413)	1.170 (0.977, 1.401)
Q4	1.035 (0.860, 1.246)	1.027 (0.853, 1.237)	1.010 (0.839, 1.217)
ITGB1BP2	1 (Reference)	1 (Reference)	1 (Reference)
Q2	1.043 (0.865, 1.257)	1.022 (0.848, 1.232)	1.015 (0.843, 1.224)
Q3	1.136 (0.946, 1.364)	1.103 (0.918, 1.324)	1.090 (0.908, 1.310)
Q4	**1.286 (1.074, 1.540)**	**1.218 (1.017, 1.458)**	1.185 (0.988, 1.420)
ITGB2	1 (Reference)	1 (Reference)	1 (Reference)
Q2	**0.714 (0.595, 0.856)**	**0.700 (0.583, 0.840)**	**0.701 (0.584, 0.841)**
Q3	**0.665 (0.554, 0.798)**	**0.647 (0.539, 0.777)**	**0.649 (0.541, 0.779)**
Q4	**0.762 (0.640, 0.908)**	**0.717 (0.602, 0.856)**	**0.724 (0.607, 0.864)**
ITGB5	1 (Reference)	1 (Reference)	1 (Reference)
Q2	**0.784 (0.649, 0.948)**	**0.818 (0.677, 0.990)**	0.844 (0.698, 1.021)
Q3	**0.761 (0.631, 0.917)**	**0.813 (0.674, 0.981)**	0.848 (0.703, 1.025)
Q4	0.867 (0.724, 1.038)	0.927 (0.773, 1.111)	0.969 (0.807, 1.164)
ITGB6	1 (Reference)	1 (Reference)	1 (Reference)
Q2	0.860 (0.719, 1.029)	0.902 (0.754, 1.079)	0.903 (0.754, 1.080)
Q3	0.879 (0.736, 1.049)	0.951 (0.795, 1.136)	0.953 (0.797, 1.139)
Q4	0.863 (0.723, 1.030)	0.954 (0.797, 1.141)	0.965 (0.806, 1.156)
ITGB7	1 (Reference)	1 (Reference)	1 (Reference)
Q2	0.843 (0.693, 1.025)	0.844 (0.694, 1.026)	0.842 (0.692, 1.024)

Q3	1.128 (0.941, 1.352)	1.104 (0.921, 1.324)	1.102 (0.919, 1.321)
Q4	1.158 (0.967, 1.387)	1.137 (0.949, 1.361)	1.139 (0.951, 1.364)
ITGBL1	1 (Reference)	1 (Reference)	1 (Reference)
Q2	0.972 (0.792, 1.193)	0.979 (0.798, 1.202)	0.974 (0.793, 1.196)
Q3	1.053 (0.865, 1.284)	1.046 (0.858, 1.275)	1.037 (0.851, 1.265)
Q4	**1.257 (1.040, 1.520)**	1.167 (0.964, 1.413)	1.130 (0.933, 1.369)

Q1 (lowest, < 25th percentile), Q2 (25th to < 50th percentile), Q3 (50th to < 75th percentile), and Q4 (highest, ⩾ 75th percentile); CI: confidence interval; ITG, Integrin.
