# Supplementary material for: The Role of Integrin Family in Atherosclerotic Cardiovascular Disease: A Prospective Cohort Study and Mendelian Randomization Analysis
Source: Biomedicines. 2026 Aug 15;14(8):1836. doi: 10.3390/biomedicines14081836 (PMC13509564; doi:10.3390/biomedicines14081836)
Supplement: Supplementary file 1 [file biomedicines-14-01836-s001.zip › TableS7.pdf]

Table S7. Sex-stratified HR (95% CI) of incident CHD by integrin levels.

	Hazard Ratio (95% CI)		
Integrin	Model 1	Model 2	Model 3
Male			
ITGA11	1 (Reference)	1 (Reference)	1 (Reference)
Q2	**0.694 (0.589, 0.817)**	**0.742 (0.630, 0.875)**	**0.768 (0.651, 0.906)**
Q3	**0.690 (0.584, 0.814)**	**0.773 (0.653, 0.914)**	**0.831 (0.701, 0.984)**
Q4	**0.579 (0.486, 0.690)**	**0.670 (0.560, 0.802)**	**0.741 (0.617, 0.889)**
ITGA2	1 (Reference)	1 (Reference)	1 (Reference)
Q2	0.969 (0.824, 1.139)	0.994 (0.845, 1.170)	1.015 (0.863, 1.194)
Q3	**0.782 (0.659, 0.928)**	**0.814 (0.686, 0.967)**	0.850 (0.716, 1.010)
Q4	**0.658 (0.550, 0.787)**	**0.693 (0.579, 0.829)**	**0.741 (0.618, 0.889)**
ITGA5	1 (Reference)	1 (Reference)	1 (Reference)
Q2	1.159 (0.957, 1.404)	1.082 (0.893, 1.311)	1.057 (0.872, 1.281)
Q3	**1.305 (1.083, 1.572)**	1.176 (0.974, 1.420)	1.146 (0.949, 1.385)
Q4	**1.698 (1.421, 2.029)**	**1.384 (1.151, 1.666)**	**1.330 (1.103, 1.603)**
ITGA6	1 (Reference)	1 (Reference)	1 (Reference)
Q2	0.994 (0.841, 1.174)	1.006 (0.851, 1.189)	1.007 (0.852, 1.190)
Q3	0.981 (0.829, 1.161)	1.013 (0.855, 1.199)	1.008 (0.851, 1.193)
Q4	**0.795 (0.663, 0.952)**	**0.818 (0.682, 0.981)**	**0.815 (0.680, 0.978)**
ITGAL	1 (Reference)	1 (Reference)	1 (Reference)
Q2	1.196 (0.996, 1.437)	1.159 (0.965, 1.392)	1.142 (0.951, 1.371)
Q3	1.106 (0.920, 1.330)	1.040 (0.865, 1.251)	1.012 (0.841, 1.218)
Q4	**1.399 (1.175, 1.666)**	**1.241 (1.041, 1.480)**	**1.199 (1.005, 1.431)**
ITGAM	1 (Reference)	1 (Reference)	1 (Reference)
Q2	0.890 (0.751, 1.056)	0.885 (0.747, 1.050)	0.910 (0.767, 1.080)
Q3	0.959 (0.811, 1.133)	0.958 (0.810, 1.132)	0.989 (0.836, 1.169)
Q4	**0.757 (0.634, 0.904)**	**0.729 (0.610, 0.873)**	**0.768 (0.642, 0.920)**
ITGAV	1 (Reference)	1 (Reference)	1 (Reference)
Q2	**0.811 (0.687, 0.958)**	0.873 (0.739, 1.032)	0.923 (0.781, 1.092)
Q3	**0.622 (0.522, 0.741)**	**0.692 (0.580, 0.826)**	**0.747 (0.626, 0.893)**
Q4	**0.624 (0.526, 0.741)**	**0.730 (0.613, 0.869)**	**0.823 (0.688, 0.984)**
ITGAX	1 (Reference)	1 (Reference)	1 (Reference)
Q2	1.155 (0.966, 1.381)	1.118 (0.935, 1.337)	1.113 (0.931, 1.331)
Q3	1.146 (0.957, 1.371)	1.120 (0.936, 1.341)	1.119 (0.935, 1.339)
Q4	**1.235 (1.036, 1.473)**	1.191 (0.999, 1.420)	1.187 (0.995, 1.415)
ITGB1	1 (Reference)	1 (Reference)	1 (Reference)
Q2	0.879 (0.743, 1.038)	0.899 (0.761, 1.063)	0.926 (0.783, 1.095)
Q3	**0.751 (0.631, 0.894)**	**0.781 (0.656, 0.929)**	**0.821 (0.690, 0.978)**
Q4	**0.765 (0.644, 0.909)**	**0.793 (0.667, 0.942)**	0.857 (0.720, 1.021)
ITGB1BP1	1 (Reference)	1 (Reference)	1 (Reference)
Q2	1.082 (0.911, 1.286)	1.112 (0.936, 1.322)	1.109 (0.933, 1.318)
Q3	0.974 (0.815, 1.164)	0.997 (0.834, 1.192)	0.993 (0.831, 1.187)

Q4	1.103 (0.927, 1.312)	1.109 (0.933, 1.320)	1.102 (0.927, 1.311)
ITGB1BP2	1 (Reference)	1 (Reference)	1 (Reference)
Q2	0.892 (0.751, 1.060)	0.887 (0.747, 1.054)	0.889 (0.748, 1.055)
Q3	0.973 (0.822, 1.151)	0.952 (0.804, 1.127)	0.959 (0.810, 1.135)
Q4	0.900 (0.756, 1.072)	0.875 (0.735, 1.042)	0.871 (0.731, 1.037)
ITGB2	1 (Reference)	1 (Reference)	1 (Reference)
Q2	0.904 (0.757, 1.080)	0.887 (0.742, 1.060)	0.885 (0.740, 1.057)
Q3	0.954 (0.801, 1.136)	0.925 (0.777, 1.102)	0.931 (0.782, 1.109)
Q4	1.024 (0.863, 1.216)	0.937 (0.789, 1.114)	0.936 (0.787, 1.112)
ITGB5	1 (Reference)	1 (Reference)	1 (Reference)
Q2	0.975 (0.820, 1.160)	1.018 (0.855, 1.211)	1.048 (0.880, 1.247)
Q3	0.909 (0.764, 1.083)	0.967 (0.812, 1.152)	1.026 (0.860, 1.223)
Q4	**0.816 (0.682, 0.976)**	0.855 (0.715, 1.024)	0.914 (0.763, 1.095)
ITGB6	1 (Reference)	1 (Reference)	1 (Reference)
Q2	0.945 (0.795, 1.123)	0.990 (0.833, 1.177)	0.994 (0.836, 1.181)
Q3	1.011 (0.853, 1.199)	1.084 (0.914, 1.286)	1.097 (0.925, 1.301)
Q4	0.873 (0.733, 1.041)	0.970 (0.813, 1.158)	0.983 (0.823, 1.174)
ITGB7	1 (Reference)	1 (Reference)	1 (Reference)
Q2	0.905 (0.761, 1.075)	0.908 (0.764, 1.079)	0.918 (0.773, 1.092)
Q3	0.905 (0.760, 1.077)	0.895 (0.752, 1.066)	0.900 (0.756, 1.071)
Q4	1.003 (0.847, 1.189)	0.964 (0.814, 1.143)	0.947 (0.799, 1.123)
ITGBL1	1 (Reference)	1 (Reference)	1 (Reference)
Q2	0.992 (0.813, 1.212)	0.989 (0.810, 1.209)	0.977 (0.799, 1.194)
Q3	1.095 (0.902, 1.328)	1.037 (0.854, 1.260)	1.006 (0.828, 1.223)
Q4	**1.405 (1.167, 1.692)**	1.197 (0.988, 1.450)	1.138 (0.937, 1.380)

	Hazard Ratio (95% CI)		
Integrin	Model 1	Model 2	Model 3
Female			
ITGA11	1 (Reference)	1 (Reference)	1 (Reference)
Q2	**0.609 (0.483, 0.768)**	**0.654 (0.518, 0.826)**	**0.671 (0.531, 0.848)**
Q3	**0.590 (0.469, 0.742)**	**0.664 (0.526, 0.839)**	**0.701 (0.554, 0.887)**
Q4	**0.437 (0.342, 0.560)**	**0.519 (0.403, 0.670)**	**0.559 (0.432, 0.724)**
ITGA2	1 (Reference)	1 (Reference)	1 (Reference)
Q2	**0.725 (0.572, 0.921)**	**0.763 (0.601, 0.968)**	**0.772 (0.608, 0.981)**
Q3	**0.677 (0.533, 0.859)**	**0.731 (0.575, 0.929)**	**0.742 (0.583, 0.945)**
Q4	**0.631 (0.497, 0.800)**	**0.691 (0.544, 0.879)**	**0.700 (0.548, 0.894)**
ITGA5	1 (Reference)	1 (Reference)	1 (Reference)
Q2	**1.538 (1.137, 2.079)**	**1.442 (1.066, 1.951)**	**1.422 (1.051, 1.924)**
Q3	**1.610 (1.198, 2.165)**	**1.453 (1.078, 1.958)**	**1.415 (1.049, 1.908)**
Q4	**1.955 (1.468, 2.603)**	**1.577 (1.172, 2.122)**	**1.490 (1.105, 2.008)**
ITGA6	1 (Reference)	1 (Reference)	1 (Reference)
Q2	1.011 (0.793, 1.288)	1.007 (0.790, 1.283)	0.993 (0.779, 1.265)

Q3	0.996 (0.779, 1.273)	0.993 (0.777, 1.270)	0.963 (0.753, 1.232)
Q4	1.047 (0.821, 1.336)	1.036 (0.812, 1.322)	1.003 (0.786, 1.280)
ITGAL	1 (Reference)	1 (Reference)	1 (Reference)
Q2	1.090 (0.839, 1.414)	1.048 (0.807, 1.361)	1.040 (0.801, 1.350)
Q3	1.116 (0.865, 1.440)	1.029 (0.797, 1.329)	1.003 (0.776, 1.296)
Q4	1.146 (0.892, 1.470)	1.011 (0.786, 1.301)	0.966 (0.750, 1.244)
ITGAM	1 (Reference)	1 (Reference)	1 (Reference)
Q2	**0.765 (0.606, 0.966)**	**0.775 (0.614, 0.979)**	**0.784 (0.620, 0.990)**
Q3	**0.674 (0.531, 0.856)**	**0.686 (0.541, 0.871)**	**0.708 (0.557, 0.899)**
Q4	**0.649 (0.511, 0.826)**	**0.666 (0.523, 0.849)**	**0.700 (0.549, 0.893)**
ITGAV	1 (Reference)	1 (Reference)	1 (Reference)
Q2	**0.765 (0.606, 0.966)**	0.814 (0.644, 1.029)	0.826 (0.653, 1.045)
Q3	**0.580 (0.455, 0.738)**	**0.657 (0.514, 0.839)**	**0.684 (0.534, 0.876)**
Q4	**0.410 (0.317, 0.530)**	**0.483 (0.371, 0.628)**	**0.516 (0.395, 0.674)**
ITGAX	1 (Reference)	1 (Reference)	1 (Reference)
Q2	0.970 (0.765, 1.231)	0.962 (0.758, 1.220)	0.961 (0.758, 1.220)
Q3	0.850 (0.665, 1.086)	0.840 (0.657, 1.073)	0.829 (0.649, 1.060)
Q4	0.952 (0.750, 1.209)	0.941 (0.741, 1.195)	0.926 (0.729, 1.177)
ITGB1	1 (Reference)	1 (Reference)	1 (Reference)
Q2	0.784 (0.613, 1.001)	0.825 (0.645, 1.054)	0.837 (0.655, 1.070)
Q3	**0.655 (0.511, 0.840)**	**0.701 (0.546, 0.899)**	**0.727 (0.565, 0.935)**
Q4	**0.612 (0.477, 0.786)**	**0.682 (0.530, 0.877)**	**0.718 (0.556, 0.926)**
ITGB1BP1	1 (Reference)	1 (Reference)	1 (Reference)
Q2	1.029 (0.802, 1.321)	1.024 (0.798, 1.314)	1.019 (0.794, 1.308)
Q3	1.197 (0.941, 1.522)	1.185 (0.931, 1.508)	1.169 (0.919, 1.488)
Q4	1.020 (0.795, 1.308)	1.014 (0.790, 1.301)	0.990 (0.772, 1.271)
ITGB1BP2	1 (Reference)	1 (Reference)	1 (Reference)
Q2	1.186 (0.921, 1.527)	1.161 (0.901, 1.495)	1.147 (0.890, 1.477)
Q3	1.206 (0.937, 1.552)	1.170 (0.909, 1.506)	1.147 (0.891, 1.477)
Q4	**1.412 (1.104, 1.806)**	**1.328 (1.038, 1.700)**	1.275 (0.995, 1.633)
ITGB2	1 (Reference)	1 (Reference)	1 (Reference)
Q2	**0.669 (0.520, 0.861)**	**0.658 (0.511, 0.847)**	**0.659 (0.512, 0.848)**
Q3	**0.742 (0.583, 0.944)**	**0.725 (0.570, 0.923)**	**0.728 (0.572, 0.927)**
Q4	0.796 (0.630, 1.007)	**0.752 (0.593, 0.953)**	**0.759 (0.599, 0.963)**
ITGB5	1 (Reference)	1 (Reference)	1 (Reference)
Q2	**0.776 (0.604, 0.996)**	0.815 (0.635, 1.047)	0.842 (0.655, 1.082)
Q3	**0.722 (0.563, 0.925)**	**0.778 (0.606, 0.998)**	0.815 (0.634, 1.047)
Q4	**0.756 (0.592, 0.964)**	0.813 (0.636, 1.038)	0.846 (0.661, 1.083)
ITGB6	1 (Reference)	1 (Reference)	1 (Reference)
Q2	0.812 (0.637, 1.035)	0.856 (0.671, 1.091)	0.854 (0.670, 1.090)
Q3	0.854 (0.673, 1.084)	0.933 (0.734, 1.186)	0.932 (0.733, 1.185)
Q4	0.880 (0.695, 1.114)	0.983 (0.775, 1.248)	0.987 (0.777, 1.254)
ITGB7	1 (Reference)	1 (Reference)	1 (Reference)
Q2	0.785 (0.603, 1.023)	0.788 (0.605, 1.026)	0.784 (0.602, 1.021)

Q3	1.033 (0.809, 1.319)	1.011 (0.792, 1.291)	1.007 (0.789, 1.286)
Q4	1.174 (0.926, 1.489)	1.150 (0.907, 1.459)	1.150 (0.906, 1.458)
ITGBL1	1 (Reference)	1 (Reference)	1 (Reference)
Q2	1.038 (0.787, 1.368)	1.041 (0.790, 1.372)	1.034 (0.784, 1.363)
Q3	1.086 (0.830, 1.420)	1.074 (0.821, 1.405)	1.059 (0.809, 1.386)
Q4	1.289 (0.996, 1.669)	1.178 (0.908, 1.528)	1.126 (0.867, 1.463)

Q1 (lowest, < 25th percentile), Q2 (25th to < 50th percentile), Q3 (50th to < 75th percentile), and Q4 (highest, ⩾ 75th percentile); CI: confidence interval; ITG, Integrin.
