# Supplementary material for: The Role of Integrin Family in Atherosclerotic Cardiovascular Disease: A Prospective Cohort Study and Mendelian Randomization Analysis
Source: Biomedicines. 2026 Aug 15;14(8):1836. doi: 10.3390/biomedicines14081836 (PMC13509564; doi:10.3390/biomedicines14081836)
Supplement: Supplementary file 1 [file biomedicines-14-01836-s001.zip › TableS8.pdf]

**Table S8. Sex-stratified HR (95% CI) of incident IS by integrin levels.**

| | Hazard Ratio (95% CI) | | |
|---|---|---|---|
| Integrin | Model 1 | Model 2 | Model 3 |
| Male | | | |
| ITGA11 | 1 (Reference) | 1 (Reference) | 1 (Reference) |
| Q2 | **0.702 (0.574, 0.858)** | **0.740 (0.605, 0.906)** | **0.773 (0.632, 0.947)** |
| Q3 | **0.678 (0.553, 0.832)** | **0.739 (0.601, 0.909)** | **0.810 (0.657, 0.998)** |
| Q4 | **0.581 (0.469, 0.721)** | **0.650 (0.522, 0.810)** | **0.739 (0.591, 0.925)** |
| ITGA2 | 1 (Reference) | 1 (Reference) | 1 (Reference) |
| Q2 | 0.921 (0.754, 1.126) | 0.941 (0.770, 1.150) | 0.965 (0.789, 1.179) |
| Q3 | **0.794 (0.644, 0.978)** | 0.821 (0.666, 1.011) | 0.865 (0.701, 1.067) |
| Q4 | **0.664 (0.534, 0.826)** | **0.691 (0.555, 0.860)** | **0.746 (0.598, 0.932)** |
| ITGA5 | 1 (Reference) | 1 (Reference) | 1 (Reference) |
| Q2 | 1.119 (0.881, 1.422) | 1.065 (0.838, 1.355) | 1.035 (0.813, 1.316) |
| Q3 | **1.534 (1.226, 1.920)** | **1.419 (1.131, 1.779)** | **1.369 (1.090, 1.718)** |
| Q4 | **1.648 (1.320, 2.058)** | **1.409 (1.119, 1.775)** | **1.328 (1.052, 1.677)** |
| ITGA6 | 1 (Reference) | 1 (Reference) | 1 (Reference) |
| Q2 | 1.066 (0.866, 1.312) | 1.075 (0.873, 1.324) | 1.076 (0.874, 1.325) |
| Q3 | 1.040 (0.843, 1.283) | 1.063 (0.862, 1.312) | 1.056 (0.855, 1.303) |
| Q4 | 0.899 (0.721, 1.121) | 0.918 (0.736, 1.146) | 0.911 (0.730, 1.138) |
| ITGAL | 1 (Reference) | 1 (Reference) | 1 (Reference) |
| Q2 | 1.237 (0.985, 1.555) | 1.208 (0.961, 1.517) | 1.186 (0.944, 1.490) |
| Q3 | 1.251 (0.998, 1.568) | 1.194 (0.952, 1.498) | 1.156 (0.921, 1.450) |
| Q4 | **1.475 (1.186, 1.834)** | **1.343 (1.078, 1.673)** | **1.286 (1.032, 1.603)** |
| ITGAM | 1 (Reference) | 1 (Reference) | 1 (Reference) |
| Q2 | 0.883 (0.715, 1.090) | 0.879 (0.712, 1.086) | 0.908 (0.735, 1.122) |
| Q3 | 0.983 (0.801, 1.207) | 0.982 (0.800, 1.206) | 1.024 (0.833, 1.257) |
| Q4 | **0.791 (0.637, 0.984)** | **0.771 (0.619, 0.960)** | 0.824 (0.661, 1.026) |
| ITGAV | 1 (Reference) | 1 (Reference) | 1 (Reference) |
| Q2 | **0.792 (0.642, 0.976)** | 0.837 (0.679, 1.033) | 0.897 (0.727, 1.108) |
| Q3 | **0.716 (0.579, 0.884)** | **0.778 (0.628, 0.963)** | 0.859 (0.692, 1.065) |
| Q4 | **0.709 (0.575, 0.873)** | **0.800 (0.646, 0.990)** | 0.932 (0.749, 1.159) |
| ITGAX | 1 (Reference) | 1 (Reference) | 1 (Reference) |
| Q2 | 1.138 (0.914, 1.418) | 1.111 (0.891, 1.384) | 1.102 (0.884, 1.373) |
| Q3 | 1.179 (0.947, 1.467) | 1.160 (0.932, 1.443) | 1.155 (0.928, 1.438) |
| Q4 | 1.222 (0.984, 1.517) | 1.190 (0.959, 1.478) | 1.182 (0.952, 1.468) |
| ITGB1 | 1 (Reference) | 1 (Reference) | 1 (Reference) |
| Q2 | 0.937 (0.765, 1.147) | 0.953 (0.778, 1.167) | 0.987 (0.806, 1.210) |
| Q3 | **0.742 (0.599, 0.921)** | **0.764 (0.616, 0.948)** | 0.810 (0.653, 1.006) |
| Q4 | **0.774 (0.626, 0.957)** | **0.794 (0.642, 0.982)** | 0.870 (0.702, 1.078) |
| ITGB1BP1 | 1 (Reference) | 1 (Reference) | 1 (Reference) |
| Q2 | 1.105 (0.893, 1.368) | 1.126 (0.910, 1.394) | 1.123 (0.908, 1.390) |
| Q3 | 1.018 (0.818, 1.267) | 1.035 (0.831, 1.288) | 1.032 (0.829, 1.285) |

| | | | |
|---|---|---|---|
| Q4 | 1.138 (0.919, 1.409) | 1.143 (0.923, 1.415) | 1.133 (0.914, 1.403) |
| ITGB1BP2 | 1 (Reference) | 1 (Reference) | 1 (Reference) |
| Q2 | 0.947 (0.765, 1.173) | 0.944 (0.762, 1.170) | 0.944 (0.762, 1.169) |
| Q3 | 1.066 (0.866, 1.314) | 1.051 (0.853, 1.294) | 1.054 (0.856, 1.299) |
| Q4 | 0.984 (0.794, 1.220) | 0.962 (0.776, 1.193) | 0.949 (0.765, 1.178) |
| ITGB2 | 1 (Reference) | 1 (Reference) | 1 (Reference) |
| Q2 | 0.885 (0.710, 1.104) | 0.872 (0.699, 1.088) | 0.872 (0.699, 1.087) |
| Q3 | 0.976 (0.788, 1.209) | 0.954 (0.770, 1.183) | 0.963 (0.777, 1.193) |
| Q4 | 1.081 (0.877, 1.332) | 1.010 (0.818, 1.247) | 1.010 (0.818, 1.247) |
| ITGB5 | 1 (Reference) | 1 (Reference) | 1 (Reference) |
| Q2 | 1.025 (0.827, 1.271) | 1.059 (0.854, 1.313) | 1.098 (0.885, 1.363) |
| Q3 | 0.972 (0.783, 1.207) | 1.018 (0.819, 1.264) | 1.092 (0.878, 1.357) |
| Q4 | 0.917 (0.736, 1.142) | 0.951 (0.763, 1.186) | 1.027 (0.823, 1.282) |
| ITGB6 | 1 (Reference) | 1 (Reference) | 1 (Reference) |
| Q2 | 0.873 (0.703, 1.084) | 0.903 (0.727, 1.121) | 0.908 (0.731, 1.127) |
| Q3 | 1.043 (0.848, 1.283) | 1.101 (0.895, 1.356) | 1.116 (0.906, 1.374) |
| Q4 | 0.926 (0.749, 1.146) | 1.008 (0.813, 1.249) | 1.023 (0.825, 1.268) |
| ITGB7 | 1 (Reference) | 1 (Reference) | 1 (Reference) |
| Q2 | 0.909 (0.732, 1.128) | 0.911 (0.733, 1.131) | 0.920 (0.741, 1.142) |
| Q3 | 0.977 (0.788, 1.210) | 0.968 (0.782, 1.200) | 0.972 (0.784, 1.204) |
| Q4 | 1.107 (0.900, 1.363) | 1.073 (0.872, 1.322) | 1.051 (0.853, 1.295) |
| ITGBL1 | 1 (Reference) | 1 (Reference) | 1 (Reference) |
| Q2 | 1.056 (0.833, 1.338) | 1.048 (0.826, 1.329) | 1.031 (0.813, 1.308) |
| Q3 | 1.088 (0.861, 1.375) | 1.040 (0.822, 1.317) | 1.000 (0.789, 1.267) |
| Q4 | **1.336 (1.064, 1.677)** | 1.170 (0.925, 1.479) | 1.092 (0.861, 1.384) |

| | Hazard Ratio (95% CI) | | |
|---|---|---|---|
| Integrin | Model 1 | Model 2 | Model 3 |
| Female | | | |
| ITGA11 | 1 (Reference) | 1 (Reference) | 1 (Reference) |
| Q2 | **0.629 (0.478, 0.828)** | **0.666 (0.505, 0.878)** | **0.676 (0.512, 0.892)** |
| Q3 | **0.599 (0.456, 0.787)** | **0.658 (0.499, 0.869)** | **0.680 (0.514, 0.900)** |
| Q4 | **0.463 (0.346, 0.619)** | **0.532 (0.395, 0.718)** | **0.555 (0.410, 0.753)** |
| ITGA2 | 1 (Reference) | 1 (Reference) | 1 (Reference) |
| Q2 | **0.743 (0.560, 0.986)** | 0.774 (0.583, 1.028) | 0.773 (0.582, 1.027) |
| Q3 | **0.722 (0.545, 0.956)** | 0.766 (0.578, 1.016) | 0.761 (0.573, 1.012) |
| Q4 | **0.644 (0.485, 0.855)** | **0.691 (0.519, 0.920)** | **0.675 (0.505, 0.903)** |
| ITGA5 | 1 (Reference) | 1 (Reference) | 1 (Reference) |
| Q2 | **1.515 (1.060, 2.163)** | **1.440 (1.007, 2.059)** | 1.417 (0.991, 2.026) |
| Q3 | **1.667 (1.177, 2.360)** | **1.537 (1.082, 2.182)** | **1.494 (1.052, 2.122)** |
| Q4 | **1.958 (1.396, 2.747)** | **1.657 (1.167, 2.352)** | **1.560 (1.096, 2.219)** |
| ITGA6 | 1 (Reference) | 1 (Reference) | 1 (Reference) |
| Q2 | 1.181 (0.887, 1.573) | 1.176 (0.884, 1.566) | 1.161 (0.872, 1.546) |

| | | | |
|---|---|---|---|
| Q3 | 1.011 (0.750, 1.364) | 1.011 (0.750, 1.364) | 0.979 (0.726, 1.321) |
| Q4 | 1.144 (0.855, 1.531) | 1.132 (0.846, 1.516) | 1.088 (0.812, 1.458) |
| ITGAL | 1 (Reference) | 1 (Reference) | 1 (Reference) |
| Q2 | 1.138 (0.832, 1.556) | 1.103 (0.807, 1.509) | 1.094 (0.800, 1.497) |
| Q3 | 1.232 (0.910, 1.668) | 1.150 (0.848, 1.559) | 1.118 (0.825, 1.517) |
| Q4 | 1.213 (0.899, 1.637) | 1.097 (0.811, 1.486) | 1.049 (0.774, 1.421) |
| ITGAM | 1 (Reference) | 1 (Reference) | 1 (Reference) |
| Q2 | 0.764 (0.581, 1.005) | 0.776 (0.590, 1.021) | 0.780 (0.593, 1.026) |
| Q3 | **0.641 (0.483, 0.852)** | **0.654 (0.492, 0.869)** | **0.670 (0.504, 0.890)** |
| Q4 | **0.647 (0.487, 0.858)** | **0.671 (0.505, 0.891)** | **0.695 (0.522, 0.925)** |
| ITGAV | 1 (Reference) | 1 (Reference) | 1 (Reference) |
| Q2 | 0.847 (0.642, 1.119) | 0.887 (0.671, 1.173) | 0.889 (0.672, 1.176) |
| Q3 | **0.663 (0.498, 0.883)** | **0.728 (0.544, 0.973)** | **0.741 (0.553, 0.993)** |
| Q4 | **0.433 (0.318, 0.591)** | **0.490 (0.357, 0.674)** | **0.504 (0.365, 0.697)** |
| ITGAX | 1 (Reference) | 1 (Reference) | 1 (Reference) |
| Q2 | 0.985 (0.746, 1.301) | 0.979 (0.741, 1.292) | 0.978 (0.740, 1.291) |
| Q3 | 0.838 (0.628, 1.118) | 0.826 (0.619, 1.102) | 0.818 (0.613, 1.092) |
| Q4 | 0.881 (0.662, 1.172) | 0.871 (0.654, 1.158) | 0.860 (0.646, 1.145) |
| ITGB1 | 1 (Reference) | 1 (Reference) | 1 (Reference) |
| Q2 | 0.784 (0.585, 1.051) | 0.817 (0.609, 1.095) | 0.819 (0.610, 1.098) |
| Q3 | **0.704 (0.525, 0.944)** | **0.745 (0.555, 1.000)** | 0.756 (0.562, 1.017) |
| Q4 | **0.644 (0.479, 0.866)** | **0.703 (0.522, 0.948)** | **0.715 (0.528, 0.968)** |
| ITGB1BP1 | 1 (Reference) | 1 (Reference) | 1 (Reference) |
| Q2 | 1.099 (0.812, 1.488) | 1.092 (0.807, 1.479) | 1.088 (0.803, 1.472) |
| Q3 | 1.317 (0.985, 1.762) | 1.300 (0.972, 1.739) | 1.281 (0.958, 1.714) |
| Q4 | 1.203 (0.895, 1.618) | 1.198 (0.891, 1.611) | 1.166 (0.867, 1.569) |
| ITGB1BP2 | 1 (Reference) | 1 (Reference) | 1 (Reference) |
| Q2 | 1.104 (0.818, 1.489) | 1.081 (0.801, 1.458) | 1.064 (0.788, 1.435) |
| Q3 | 1.257 (0.940, 1.682) | 1.227 (0.917, 1.641) | 1.195 (0.893, 1.600) |
| Q4 | 1.290 (0.963, 1.728) | 1.224 (0.914, 1.641) | 1.165 (0.868, 1.564) |
| ITGB2 | 1 (Reference) | 1 (Reference) | 1 (Reference) |
| Q2 | **0.665 (0.496, 0.892)** | **0.658 (0.491, 0.883)** | **0.658 (0.490, 0.883)** |
| Q3 | **0.716 (0.540, 0.950)** | **0.707 (0.532, 0.938)** | **0.708 (0.533, 0.940)** |
| Q4 | **0.735 (0.556, 0.971)** | **0.710 (0.536, 0.941)** | **0.714 (0.539, 0.946)** |
| ITGB5 | 1 (Reference) | 1 (Reference) | 1 (Reference) |
| Q2 | 0.827 (0.617, 1.110) | 0.860 (0.641, 1.155) | 0.877 (0.653, 1.178) |
| Q3 | **0.733 (0.545, 0.986)** | 0.776 (0.576, 1.046) | 0.799 (0.593, 1.078) |
| Q4 | 0.787 (0.589, 1.052) | 0.833 (0.623, 1.115) | 0.844 (0.629, 1.131) |
| ITGB6 | 1 (Reference) | 1 (Reference) | 1 (Reference) |
| Q2 | 0.893 (0.669, 1.192) | 0.932 (0.698, 1.244) | 0.925 (0.693, 1.235) |
| Q3 | 0.931 (0.700, 1.237) | 0.998 (0.750, 1.328) | 0.987 (0.741, 1.315) |
| Q4 | 0.947 (0.714, 1.256) | 1.032 (0.776, 1.373) | 1.016 (0.762, 1.353) |
| ITGB7 | 1 (Reference) | 1 (Reference) | 1 (Reference) |
| Q2 | 0.791 (0.578, 1.083) | 0.797 (0.582, 1.091) | 0.789 (0.577, 1.080) |

| | | | |
|---|---|---|---|
| Q3 | 1.076 (0.806, 1.435) | 1.057 (0.792, 1.411) | 1.048 (0.785, 1.399) |
| Q4 | 1.191 (0.898, 1.579) | 1.173 (0.884, 1.556) | 1.168 (0.880, 1.549) |
| ITGBL1 | 1 (Reference) | 1 (Reference) | 1 (Reference) |
| Q2 | 1.089 (0.775, 1.531) | 1.091 (0.776, 1.533) | 1.083 (0.770, 1.523) |
| Q3 | 1.332 (0.965, 1.840) | 1.319 (0.955, 1.823) | 1.298 (0.939, 1.793) |
| Q4 | **1.521 (1.111, 2.082)** | **1.414 (1.031, 1.941)** | 1.355 (0.986, 1.862) |

Q1 (lowest, < 25th percentile), Q2 (25th to < 50th percentile), Q3 (50th to < 75th percentile), and Q4 (highest, ⩾ 75th percentile); CI: confidence interval; ITG, Integrin.
