# Supplementary material for: The Role of Integrin Family in Atherosclerotic Cardiovascular Disease: A Prospective Cohort Study and Mendelian Randomization Analysis
Source: Biomedicines. 2026 Aug 15;14(8):1836. doi: 10.3390/biomedicines14081836 (PMC13509564; doi:10.3390/biomedicines14081836)
Supplement: Supplementary file 1 [file biomedicines-14-01836-s001.zip › TableS9.pdf]

Table S9. Sex-stratified HR (95% CI) of incident PAD by integrin levels.

	Hazard Ratio (95% CI)		
Integrin	Model 1	Model 2	Model 3
Male			
ITGA11	1 (Reference)	1 (Reference)	1 (Reference)
Q2	**0.584 (0.445, 0.768)**	**0.675 (0.513, 0.889)**	**0.682 (0.518, 0.899)**
Q3	**0.552 (0.416, 0.731)**	**0.669 (0.503, 0.889)**	**0.680 (0.510, 0.907)**
Q4	**0.483 (0.359, 0.651)**	**0.635 (0.469, 0.860)**	**0.651 (0.478, 0.886)**
ITGA2	1 (Reference)	1 (Reference)	1 (Reference)
Q2	0.971 (0.740, 1.274)	1.004 (0.765, 1.317)	1.006 (0.767, 1.321)
Q3	0.769 (0.577, 1.025)	0.834 (0.625, 1.112)	0.840 (0.629, 1.121)
Q4	**0.626 (0.462, 0.848)**	**0.692 (0.511, 0.939)**	**0.698 (0.513, 0.951)**
ITGA5	1 (Reference)	1 (Reference)	1 (Reference)
Q2	1.130 (0.798, 1.601)	1.039 (0.732, 1.473)	1.031 (0.726, 1.463)
Q3	**1.512 (1.089, 2.099)**	1.350 (0.969, 1.880)	1.339 (0.961, 1.867)
Q4	**2.287 (1.683, 3.109)**	**1.747 (1.270, 2.404)**	**1.728 (1.252, 2.386)**
ITGA6	1 (Reference)	1 (Reference)	1 (Reference)
Q2	1.006 (0.757, 1.337)	1.028 (0.773, 1.366)	1.030 (0.775, 1.369)
Q3	1.008 (0.757, 1.343)	1.072 (0.804, 1.429)	1.068 (0.801, 1.424)
Q4	0.921 (0.683, 1.243)	0.981 (0.727, 1.325)	0.975 (0.721, 1.317)
ITGAL	1 (Reference)	1 (Reference)	1 (Reference)
Q2	1.258 (0.916, 1.726)	1.213 (0.883, 1.666)	1.207 (0.879, 1.657)
Q3	1.273 (0.932, 1.739)	1.176 (0.860, 1.607)	1.164 (0.851, 1.592)
Q4	**1.477 (1.093, 1.996)**	1.274 (0.941, 1.726)	1.256 (0.926, 1.702)
ITGAM	1 (Reference)	1 (Reference)	1 (Reference)
Q2	**0.612 (0.460, 0.814)**	**0.638 (0.479, 0.849)**	**0.642 (0.482, 0.855)**
Q3	**0.669 (0.507, 0.884)**	**0.707 (0.535, 0.934)**	**0.714 (0.540, 0.943)**
Q4	**0.613 (0.460, 0.816)**	**0.633 (0.474, 0.845)**	**0.642 (0.481, 0.859)**
ITGAV	1 (Reference)	1 (Reference)	1 (Reference)
Q2	**0.752 (0.572, 0.989)**	0.858 (0.652, 1.130)	0.868 (0.658, 1.145)
Q3	**0.569 (0.426, 0.759)**	**0.681 (0.509, 0.912)**	**0.692 (0.516, 0.929)**
Q4	**0.487 (0.363, 0.654)**	**0.625 (0.463, 0.844)**	**0.640 (0.471, 0.871)**
ITGAX	1 (Reference)	1 (Reference)	1 (Reference)
Q2	1.222 (0.893, 1.672)	1.147 (0.838, 1.570)	1.146 (0.837, 1.568)
Q3	1.257 (0.920, 1.717)	1.197 (0.876, 1.636)	1.196 (0.875, 1.634)
Q4	**1.550 (1.152, 2.087)**	**1.458 (1.082, 1.964)**	**1.457 (1.081, 1.962)**
ITGB1	1 (Reference)	1 (Reference)	1 (Reference)
Q2	0.877 (0.659, 1.168)	0.934 (0.701, 1.244)	0.942 (0.707, 1.255)
Q3	0.836 (0.626, 1.117)	0.896 (0.670, 1.197)	0.910 (0.680, 1.217)
Q4	0.806 (0.603, 1.078)	0.883 (0.660, 1.182)	0.903 (0.673, 1.212)
ITGB1BP1	1 (Reference)	1 (Reference)	1 (Reference)
Q2	0.967 (0.717, 1.305)	1.000 (0.740, 1.349)	0.997 (0.738, 1.346)
Q3	1.002 (0.742, 1.352)	1.047 (0.776, 1.414)	1.043 (0.773, 1.408)

Q4	1.242 (0.933, 1.654)	1.243 (0.933, 1.655)	1.236 (0.928, 1.646)
ITGB1BP2	1 (Reference)	1 (Reference)	1 (Reference)
Q2	1.234 (0.912, 1.669)	1.222 (0.904, 1.653)	1.222 (0.903, 1.653)
Q3	1.129 (0.829, 1.538)	1.099 (0.807, 1.498)	1.102 (0.809, 1.502)
Q4	**1.518 (1.131, 2.038)**	**1.454 (1.083, 1.952)**	**1.453 (1.080, 1.953)**
ITGB2	1 (Reference)	1 (Reference)	1 (Reference)
Q2	0.848 (0.634, 1.134)	0.858 (0.641, 1.148)	0.854 (0.638, 1.144)
Q3	**0.678 (0.499, 0.921)**	**0.688 (0.506, 0.934)**	**0.689 (0.507, 0.936)**
Q4	0.985 (0.746, 1.299)	0.937 (0.708, 1.238)	0.932 (0.705, 1.233)
ITGB5	1 (Reference)	1 (Reference)	1 (Reference)
Q2	0.958 (0.711, 1.290)	1.051 (0.780, 1.417)	1.062 (0.787, 1.432)
Q3	0.906 (0.673, 1.219)	1.001 (0.743, 1.349)	1.023 (0.758, 1.380)
Q4	0.886 (0.657, 1.193)	0.965 (0.716, 1.302)	0.985 (0.728, 1.332)
ITGB6	1 (Reference)	1 (Reference)	1 (Reference)
Q2	0.967 (0.728, 1.283)	1.047 (0.789, 1.391)	1.047 (0.788, 1.390)
Q3	0.885 (0.663, 1.183)	0.973 (0.728, 1.301)	0.973 (0.728, 1.302)
Q4	0.820 (0.611, 1.101)	0.960 (0.714, 1.292)	0.959 (0.712, 1.291)
ITGB7	1 (Reference)	1 (Reference)	1 (Reference)
Q2	1.086 (0.804, 1.466)	1.097 (0.813, 1.481)	1.099 (0.814, 1.484)
Q3	0.975 (0.714, 1.331)	0.979 (0.717, 1.337)	0.980 (0.717, 1.338)
Q4	**1.435 (1.081, 1.907)**	**1.363 (1.025, 1.811)**	**1.352 (1.016, 1.797)**
ITGBL1	1 (Reference)	1 (Reference)	1 (Reference)
Q2	1.346 (0.921, 1.968)	1.340 (0.916, 1.960)	1.335 (0.912, 1.954)
Q3	1.323 (0.909, 1.925)	1.226 (0.841, 1.787)	1.216 (0.834, 1.774)
Q4	**2.458 (1.740, 3.474)**	**1.985 (1.392, 2.829)**	**1.959 (1.372, 2.798)**

	Hazard Ratio (95% CI)		
Integrin	Model 1	Model 2	Model 3
Female			
ITGA11	1 (Reference)	1 (Reference)	1 (Reference)
Q2	**0.663 (0.470, 0.936)**	0.716 (0.506, 1.013)	0.750 (0.529, 1.063)
Q3	**0.582 (0.410, 0.827)**	**0.665 (0.465, 0.950)**	0.725 (0.506, 1.039)
Q4	**0.457 (0.316, 0.663)**	**0.551 (0.376, 0.808)**	**0.623 (0.422, 0.919)**
ITGA2	1 (Reference)	1 (Reference)	1 (Reference)
Q2	0.780 (0.543, 1.120)	0.829 (0.577, 1.192)	0.865 (0.601, 1.245)
Q3	**0.601 (0.409, 0.881)**	**0.653 (0.444, 0.960)**	0.693 (0.470, 1.021)
Q4	0.825 (0.584, 1.165)	0.917 (0.647, 1.300)	0.994 (0.697, 1.419)
ITGA5	1 (Reference)	1 (Reference)	1 (Reference)
Q2	1.065 (0.681, 1.667)	0.987 (0.630, 1.546)	0.975 (0.622, 1.528)
Q3	1.373 (0.902, 2.091)	1.228 (0.804, 1.877)	1.198 (0.783, 1.832)
Q4	**1.723 (1.151, 2.581)**	1.359 (0.893, 2.067)	1.292 (0.847, 1.971)
ITGA6	1 (Reference)	1 (Reference)	1 (Reference)
Q2	**0.667 (0.467, 0.954)**	**0.669 (0.468, 0.957)**	**0.657 (0.459, 0.940)**

Q3	0.824 (0.587, 1.158)	0.827 (0.589, 1.162)	0.801 (0.570, 1.127)
Q4	**0.633 (0.437, 0.915)**	**0.631 (0.436, 0.914)**	**0.614 (0.424, 0.890)**
ITGAL	1 (Reference)	1 (Reference)	1 (Reference)
Q2	0.793 (0.539, 1.169)	0.761 (0.516, 1.121)	0.755 (0.512, 1.113)
Q3	0.930 (0.645, 1.341)	0.845 (0.586, 1.220)	0.829 (0.574, 1.197)
Q4	0.915 (0.639, 1.311)	0.789 (0.549, 1.135)	0.756 (0.525, 1.089)
ITGAM	1 (Reference)	1 (Reference)	1 (Reference)
Q2	**0.685 (0.486, 0.966)**	**0.691 (0.490, 0.974)**	**0.702 (0.498, 0.991)**
Q3	**0.616 (0.434, 0.874)**	**0.623 (0.439, 0.884)**	**0.649 (0.457, 0.921)**
Q4	**0.502 (0.346, 0.729)**	**0.501 (0.344, 0.728)**	**0.536 (0.368, 0.781)**
ITGAV	1 (Reference)	1 (Reference)	1 (Reference)
Q2	0.713 (0.503, 1.010)	0.767 (0.540, 1.088)	0.796 (0.560, 1.132)
Q3	**0.529 (0.368, 0.761)**	**0.608 (0.420, 0.879)**	**0.654 (0.451, 0.948)**
Q4	**0.399 (0.273, 0.583)**	**0.482 (0.326, 0.712)**	**0.545 (0.366, 0.811)**
ITGAX	1 (Reference)	1 (Reference)	1 (Reference)
Q2	0.872 (0.594, 1.279)	0.866 (0.590, 1.271)	0.868 (0.592, 1.274)
Q3	1.044 (0.726, 1.503)	1.027 (0.713, 1.478)	1.011 (0.702, 1.456)
Q4	1.173 (0.822, 1.675)	1.159 (0.812, 1.655)	1.141 (0.799, 1.629)
ITGB1	1 (Reference)	1 (Reference)	1 (Reference)
Q2	**0.573 (0.389, 0.845)**	**0.605 (0.410, 0.893)**	**0.628 (0.425, 0.927)**
Q3	**0.624 (0.431, 0.903)**	**0.673 (0.464, 0.975)**	0.725 (0.499, 1.053)
Q4	**0.697 (0.488, 0.996)**	0.784 (0.547, 1.122)	0.873 (0.606, 1.257)
ITGB1BP1	1 (Reference)	1 (Reference)	1 (Reference)
Q2	1.192 (0.832, 1.709)	1.188 (0.829, 1.702)	1.181 (0.824, 1.692)
Q3	1.056 (0.730, 1.528)	1.041 (0.720, 1.506)	1.032 (0.713, 1.493)
Q4	0.923 (0.631, 1.351)	0.915 (0.625, 1.340)	0.895 (0.611, 1.311)
ITGB1BP2	1 (Reference)	1 (Reference)	1 (Reference)
Q2	0.939 (0.650, 1.356)	0.913 (0.632, 1.318)	0.906 (0.627, 1.308)
Q3	0.933 (0.645, 1.349)	0.896 (0.619, 1.296)	0.885 (0.611, 1.281)
Q4	1.141 (0.800, 1.628)	1.064 (0.745, 1.519)	1.028 (0.718, 1.470)
ITGB2	1 (Reference)	1 (Reference)	1 (Reference)
Q2	0.883 (0.626, 1.245)	0.856 (0.607, 1.209)	0.859 (0.609, 1.213)
Q3	**0.484 (0.325, 0.720)**	**0.465 (0.313, 0.693)**	**0.469 (0.315, 0.698)**
Q4	**0.701 (0.491, 0.999)**	**0.636 (0.444, 0.911)**	**0.648 (0.452, 0.928)**
ITGB5	1 (Reference)	1 (Reference)	1 (Reference)
Q2	0.845 (0.584, 1.222)	0.886 (0.612, 1.283)	0.932 (0.643, 1.351)
Q3	**0.626 (0.424, 0.924)**	0.678 (0.459, 1.002)	0.727 (0.491, 1.077)
Q4	0.822 (0.572, 1.180)	0.890 (0.619, 1.280)	0.965 (0.669, 1.392)
ITGB6	1 (Reference)	1 (Reference)	1 (Reference)
Q2	1.114 (0.773, 1.607)	1.185 (0.821, 1.710)	1.194 (0.827, 1.724)
Q3	0.934 (0.638, 1.367)	1.038 (0.708, 1.522)	1.049 (0.715, 1.539)
Q4	1.133 (0.788, 1.627)	1.295 (0.898, 1.868)	1.342 (0.929, 1.938)
ITGB7	1 (Reference)	1 (Reference)	1 (Reference)
Q2	0.828 (0.537, 1.277)	0.830 (0.538, 1.281)	0.833 (0.540, 1.285)

Q3	**1.491 (1.023, 2.175)**	1.441 (0.988, 2.102)	1.443 (0.989, 2.105)
Q4	**1.546 (1.063, 2.248)**	**1.495 (1.027, 2.175)**	**1.504 (1.034, 2.189)**
ITGBL1	1 (Reference)	1 (Reference)	1 (Reference)
Q2	0.751 (0.494, 1.143)	0.761 (0.500, 1.159)	0.758 (0.498, 1.154)
Q3	0.905 (0.611, 1.341)	0.898 (0.605, 1.331)	0.892 (0.601, 1.324)
Q4	1.221 (0.845, 1.764)	1.115 (0.769, 1.617)	1.068 (0.735, 1.553)

Q1 (lowest, < 25th percentile), Q2 (25th to < 50th percentile), Q3 (50th to < 75th percentile), and Q4 (highest, ≥ 75th percentile); CI: confidence interval; ITG, Integrin.
